# Enhancing knowledge discovery from cancer genomics data with Galaxy

**DOI:** 10.1093/gigascience/gix015

**Published:** 2017-03-09

**Authors:** Marco A. Albuquerque, Bruno M. Grande, Elie J. Ritch, Prasath Pararajalingam, Selin Jessa, Martin Krzywinski, Jasleen K. Grewal, Sohrab P. Shah, Paul C. Boutros, Ryan D. Morin

**Affiliations:** 1 Department of Molecular Biology and Biochemistry, Simon Fraser University, Burnaby, BC, Canada; 2 Canada's Michael Smith Genome Sciences Center, BC Cancer Agency, Vancouver, BC, Canada; 3 Department of Pathology, University of British Columbia, Vancouver, BC, Canada; 4 Ontario Institute for Cancer Research, Toronto, ON, Canada

**Keywords:** Lymphoma, Driver, Cancer, Genome, Pipeline, Workflow, Tool, Cloud

## Abstract

The field of cancer genomics has demonstrated the power of massively parallel sequencing techniques to inform on the genes and specific alterations that drive tumor onset and progression. Although large comprehensive sequence data sets continue to be made increasingly available, data analysis remains an ongoing challenge, particularly for laboratories lacking dedicated resources and bioinformatics expertise. To address this, we have produced a collection of Galaxy tools that represent many popular algorithms for detecting somatic genetic alterations from cancer genome and exome data. We developed new methods for parallelization of these tools within Galaxy to accelerate runtime and have demonstrated their usability and summarized their runtimes on multiple cloud service providers. Some tools represent extensions or refinement of existing toolkits to yield visualizations suited to cohort-wide cancer genomic analysis. For example, we present Oncocircos and Oncoprintplus, which generate data-rich summaries of exome-derived somatic mutation. Workflows that integrate these to achieve data integration and visualizations are demonstrated on a cohort of 96 diffuse large B-cell lymphomas and enabled the discovery of multiple candidate lymphoma-related genes. Our toolkit is available from our GitHub repository as Galaxy tool and dependency definitions and has been deployed using virtualization on multiple platforms including Docker.

## Introduction

An inherent problem in the application of genomics to understand the molecular aetiology of cancer is the multi-disciplinary skillset required for researchers to draw meaningful inferences from high-throughput biological data. With the rise in popularity of high-throughput DNA sequencing, the bottleneck for novel discovery has shifted from data generation to data analysis and interpretation. Although myriad algorithms have been developed to efficiently analyze large datasets, these are often tailored for technically inclined users. Software for these analyses is typically run at the command line; operation requires the use of cryptic parameters; and installation is often burdensome. Achieving a flow of data between tools is also often nontrivial and, owing to a paucity of data standards, can involve error-prone data manipulation and reformatting steps often relying on a collection of custom scripts that are often not released with publications. Combined with a necessity for high-performance computational hardware to run many such tools efficiently, these issues produce a tremendous barrier for new users.

There exist a handful of options that address this predicament in genomics as a whole. Tools that automate pipeline development such as Kronos [1], Nextflow [2], and Snakemake [3] can satisfy the needs of more technically savvy users. Alternatively, graphical user interfaces (GUIs)—which are generally lacking in the field of bioinformatics—aid in users learning the utility of the associated with command-line interfaces but typically do not scale to large data sets. Examples of genomics tools offering web-accessible GUIs include BLAST [4], VAGUE [5], and limmaGUI [6]. However, beyond an inability to scale, web-based utilities pose several issues, including design inconsistency, redundant efforts in interface development, and the inability to automatically link individual tasks into pipelines or workflows. Ideally, any reduction in the barriers associated with running individual algorithms passing data between software tools should accelerate analytical tasks and reduce the risk of errors.

To overcome this, GUI-enabled software for automating pipeline development improves the reproducibility, accessibility, and transparency of running genomic analyses [7]. Examples include Galaxy [8], Taverna [9], Pegasus [10], and commercial software packages such as Geneious [11]. In particular, the Galaxy project offers many attractive features for this goal while remaining open-source. Namely, Galaxy boasts extensive documentation, support for automatic tool installation, and the ability to instantiate public or private “cloud clusters” by leveraging CloudMan [12] and is as a whole supported by a vibrant community that provides ongoing development to the software and dedication to increasing the availability of bioinformatics software. Although algorithms for handling high-throughput sequence data are steadily being added to Galaxy, there currently remains a lack of tools and workflows tailored to perform common tasks involved in analyzing cancer genome and exome sequence data. Here, we have begun to address this issue by adapting many of the popular tools for analyzing cancer genome and exome data for Galaxy and made these publicly available as the Galaxy Cancer Genomics Toolkit.

Diffuse large B-cell lymphoma (DLBCL) is a common aggressive non-Hodgkin lymphoma that demonstrates extensive genetic heterogeneity with some genetic features found more common in only one of the two molecular subgroups, namely the ABC and GCB subgroups. Primary mediastinal B-cell lymphoma is defined as a separate entity by the World Health Organization with distinct clinical and diagnostic features but shares some genetic features with DLBCL and other lymphomas. Herein, we demonstrate the utility of the Galaxy Cancer Genomics Toolkit by applying the included workflows to a large cohort of DLBCL patients (n = 96) and through a combination of analytical and exploratory approaches leveraging multiple visualization tools implemented within the Toolkit, we uncover new candidate lymphoma-related genes and putative genetic features associated with each molecular subgroup.

## Implementing cancer genomics tools in Galaxy

We produced a comprehensive toolkit comprising a suite of complementary tools and workflows that perform many of the routine analytical tasks in cancer genomics. These include several popular methods for detecting (“calling”) somatic single nucleotide variants (SNVs), copy number variations (CNVs) and structural variations (SVs) in tumor-normal pairs. We developed additional tools to perform the many auxiliary steps helper functions that allow tools to be linked and applied generically, such as bam and text file pre- and postprocessing, manipulating, and converting file formats; variant annotation; identification of significantly mutated genes; and visualizations for performing exploratory analysis and cohort-level data summarization. The tools and helper functions are briefly detailed in Table 1 and Table S1, respectively, and documented in our repository.

**Table 1:** Main tools currently comprising the cancer genomics toolkit

Tool	Category	Reference
MutationSeq^a^	SNV detection	[30]
Strelka^a^	SNV and indel detection	[31]
SomaticSniper^a^	SNV detection	[32]
RADIA^a^	SNV detection	[33]
VarDict (Java)^a^	SNV detection	[34]
DELLY^a^	SV detection	[35]
LUMPY^a^	SV detection	[36]
Pindel^a^	SV and indel detection	[37]
Manta^a^	SV detection	[38]
Sequenza^a^	CNV detection	[39]
TITAN^a^	CNV detection	[40]
Ensembl VEP^b^	SNV Annotation	[41]
PyClone^a^	Clonal structure	[42]
EXPANDS^a^	Clonal structure	[43]
MutSigCV^a^	Significantly mutated genes	[44]
Oncodrive-FM^a^	Significantly mutated genes	[45]
GISTIC^a^	Significantly mutated genes	[46]
Maftools (oncostrip, oncodriveclust, oncoplot,	Visualization, significantly	[20]
trinucleotide plot, genecloud, MAF summary, rainfall	mutated genes, mutation	
plot, lollipop plot)^a^	signatures	
Oncocircos^c^	Visualization	[20]
Oncoprintplus^c^	Visualization	[47]
igv_screenshot^b^	Visualization	

Tools representing existing or extended analysis approaches are shown above. For a current list of tools available, refer to the repository.

a New implementation of tool for existing software.

b Existing Galaxy tool modified or extended for this project.

c New tool or visualization method created for this project.

To integrate individual tools into Galaxy, we implemented XML-based configuration files, which dictate the available inputs and arguments and build the command based on user-specified parameters. We adhered to a consistent design across tools of similar types. Planemo, an integrated development environment for Galaxy tools, was used to assist with tool creation and ensure tool versions in a Github repository and Galaxy Toolshed were in sync [13]. All repositories are available on the public Galaxy test toolshed (https://testtoolshed.g2.bx.psu.edu/), which allows users to automatically install any tool [14]. Modular tool dependency repositories provide the step-by-step instructions for automatic download and installation of dependencies. Previously defined repositories were recycled if available. Though we could not successfully produce tools that automatically install on all platforms, many of our tools successfully install (with dependencies) on the standard Galaxy Amazon Web Services (AWS) image and in a custom Ubuntu installation (v16.04). We also note that the Galaxy community is migrating towards utilizing Conda for package management, which should ameliorate many of these issues moving forward. Synthetic alignment data containing artificial variants were generated and bundled with variant callers to enable automatic testing [15]. To handle reference data, we have developed tools to use Galaxy data tables and, for simplicity, allow the option of user-provided reference data [16].

**Figure 1:**
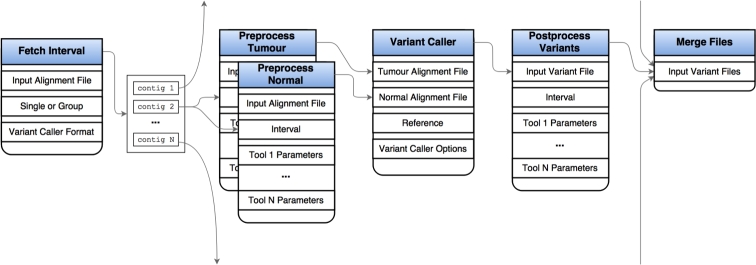
Parallelization in variant calling and other CPU-intensive processes. An alignment file flows through to fetch_interval, which fetches all contigs. If parallelization is requested, multiple files are generated for each interval, some of which may contain multiple intervals, otherwise a single file is created. Each dataset in the collection is treated as separate input to two instance of preprocess, which filters reads from both normal and tumor alignment files. These then pass to the variant caller. A postprocess tool filters and annotates variant calls based on tool-specific parameters and all final variants are merged and sorted in a single variant file. We perform automatic interval pairing to roughly balance the load on each variant-calling task. The algorithm combines regions (e.g., chromosomes) if their total length is less than the largest. In cloud-based settings, this reduces overhead associated with creating multiple unnecessary parallelized jobs as well as reducing the number of short-lived automatically added nodes. Importantly, we chose not to implement a subchromosomal interval selection algorithm to maintain intrachromosomal dependence required by some of the variant calling algorithms. Such an extension could be implemented for tools that lack this restriction.

Support within Galaxy for processing large data sets is still being established and one remaining restriction has been the lack of methods for splitting and parallelizing large analyses. We invested substantial effort to ensure that tools that perform analyses suitable for input-splitting are parallelizable on cluster environments wherever it was deemed desirable and possible. Following the addition of new data types in the Galaxy codebase, this was subsequently reimplemented using the more transparent and efficient method that exploits the more recent Galaxy feature known as “data collections.” Briefly, parallelization of a workflow is accomplished by a combination of tasks (Fig. 1), beginning with fetch_interval. This obtains chromosome size information from each input read alignment file and creates a collection of BED files defining all complete chromosome intervals available to each tool. To balance the load across all concurrently spawned jobs, we automatically pair large and small chromosomes if their sum is less than or equal to the length of the largest chromosome and we avoid splitting chromosomes into smaller intervals. This mimics the assumed longest lasting step for these algorithms while limiting the number of unnecessary concurrent jobs. The second, optional stage, is a preprocess tool that defines all necessary preprocessing steps in a single tool and all will be executed together to reduce the numerous outputs associated with running multiple separate preprocessing tools in Galaxy. This includes a samtools flag and mapping quality filter, samtools remove duplicates, and bamutils clipoverlap. The third stage launches the selected tool on each of the intervals, allowing Galaxy to spawn processes to available CPUs. The fourth stage is postprocess, which follows similar methodology to preprocess. Example usage includes further variant filtration and annotation steps. Finally all output files are merged, if necessary, so they may be supplied to subsequent tools and workflows. For tools that can be multithreaded, we instead leverage this capability rather than chromosomal splitting and note that certain tools, for example structural variant callers, cannot be generally parallelized by input splitting.

The Galaxy toolkit has been thoroughly tested on multiple hardware configurations. Many of the analyses and workflows shown in detail here were performed on a local Galaxy instance on a Dell PowerEdge R430 Server with 2x Intel Xeon Processors (32 threads total) and 384 Gb of RAM running Ubuntu linux, whereas benchmarking was performed separately on a Galaxy cluster on AWS Elastic Cloud Compute (Methods). For computationally demanding tasks, we launched a Galaxy instance on AWS Elastic Cloud Compute using CloudMan and installed the workflow and tool dependencies. A cluster consisting of one r3.8xlarge master node and five r3.2xlarge worker nodes was selected. From our experience, we advise using any nodes from the r3 family when running several jobs concurrently (cohort) as NFS issues may arise when using general-purpose nodes. If a few samples are to be run, general-purpose nodes are recommended. Separately, we produced a Dockerfile that will install our tools and additional dependencies using the galaxy-stable Docker image (https://hub.docker.com/r/bgruening/galaxy-stable/∼/dockerfile/). This is available in our GitHub repository, which also hosts the individual tools (https://github.com/morinlab/tools-morinlab). This Docker image was successfully built with automatic installation of tools and dependencies on our local Linux server and on the Google Cloud. We are working with this service to release an instance with reference genomes preinstalled that can be directly launched with minimal knowledge of Docker.

## Selecting high-value tools and developing workflows for routine analytical tasks

There are numerous algorithms available to perform standard analytical tasks such as variant calling and CNV detection, each offering different balances of usability, computational efficiency, and accuracy. As such, selection of ideal tools and parameters is nontrivial. We implemented tools representing some of the more commonly cited options and include many that performed favorably in ICGC-The Cancer Genome Atlas (TCGA) DREAM challenges [17]. As each tool can be configured with a number of parameters, which can be tuned for accuracy, we leverage results from the DREAM challenge to assist in selecting the more accurate algorithms and in setting sensible default parameters [18]. As ensemble approaches tend to provide increased robustness, we developed a tool to integrate variant calls from multiple algorithms using a simple voting scheme (Additional Items: Fig. S1). We have also released numerous workflows that run some of the more complicated pieces of software that rely on many dependencies and that perform some routine analytical and visualization tasks as detailed and illustrated with the real-world worked examples below. Example workflows that demonstrate our new approach to perform parallelization in Galaxy are also included (Fig. 1 and Additional items: Fig. S2).

## Benchmarking parallelized workflows running on Amazon Web Services

**Figure 2:**
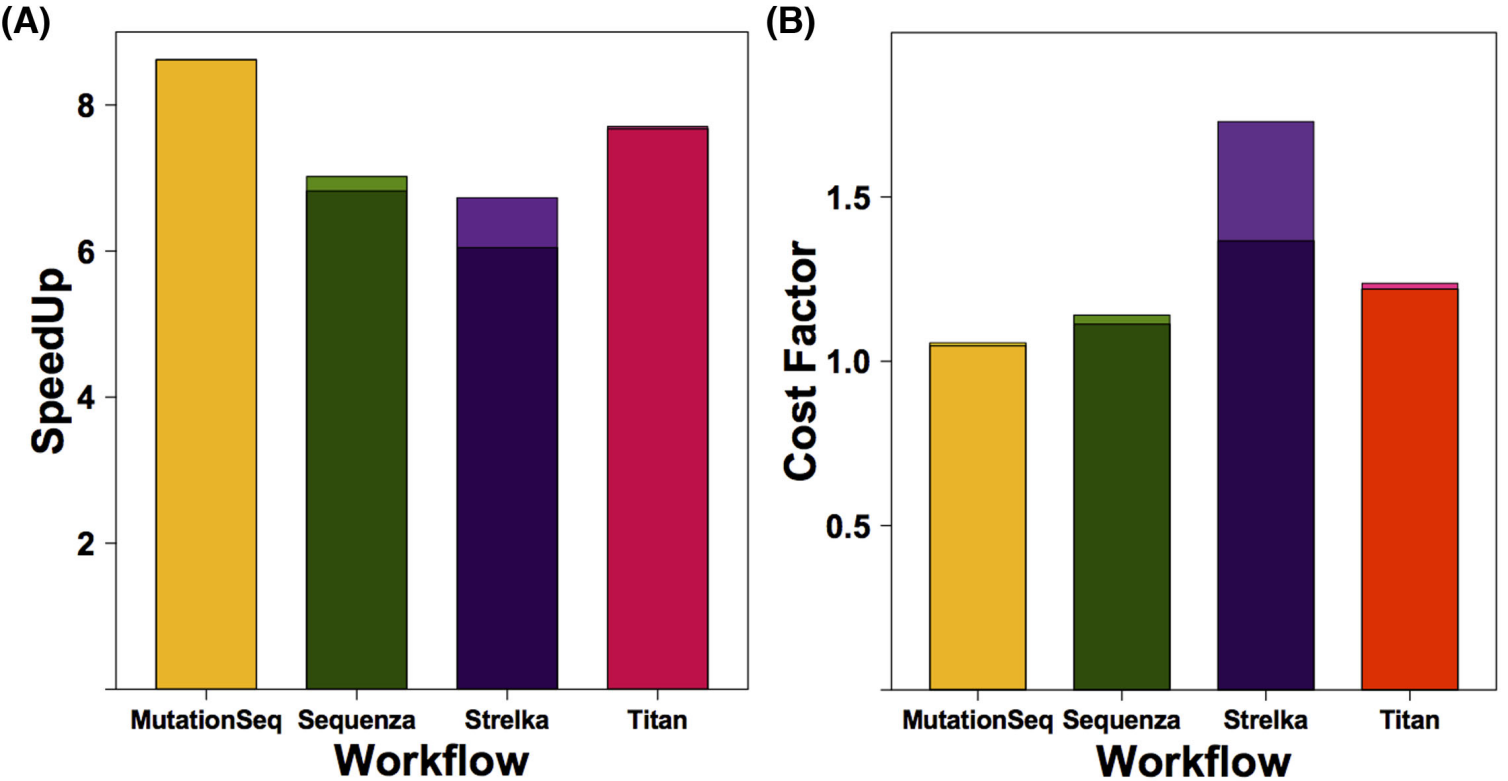
Execution time and cost differences associated with parallelization. (**A**) The average reduction in execution time was determined for all exome pairs analyzed on AWS by comparing the runtime with and without parallelization and is shown as the speedup. The preprocessing involved in setting up parallel tasks results in a reduced speedup and depends on the tool. (**B**) The actual CPU usage and dedicated machine type used by parallel implementations of the tool is shown as an estimate of the cost. Idle instances were not considered in this calculation. The difference in speedup and cost when preprocessing, the optional bam filtering step, is considered is reflected in the upper region of each bar. Note, lighter shades show runtime statistics on workflows lacking any preprocessing step, limiting that steps ability to abstract poor performance.

We uploaded 96 bam files representing the cohort of published DLBCL samples and ran all SNV and CNV workflows on this configuration for each tumor/normal pair and captured details on runtime and speedup associated with parallelization [19]. To assess the overhead potentially introduced when running tools in parallel, we ran the above cohort in both sequential and parallel modes across four different variant calling workflows. The runtime for each tool was collected from the local Galaxy database and summarized across the 48 pairs analyzed in the cloud. The average workflow runtime for an exome in parallel and sequential modes is shown in Table 2. The net change in estimated cost and speedup was averaged for each exome pair using the time taken to complete all stages of the workflow (Fig. 2). In general, the overhead associated with preprocessing adds marginally to the cost and yielded gains in speed as high as 8.6x. By comparing the cost gain and speedup in Fig. 2, it is evident that some tools, for example Strelka, do not benefit from this mode of parallelization and instead should be run using the native parallelization on a node with more threads available. In the case of Strelka, there is a substantial preprocessing that occurs on each task to prepare the directory structure and configuration files, which is repeated several times using our parallelization method. Based on the current cost models, the approximate costs for running a single TN exome pair on all four workflows on AWS is $4.27 using sequential and $5.15 using parallelization, not including the cost of uploading the files or storing these or the reference files.

**Table 2:** Average CPU usage in hours when applying variant calling workflows to exome pairs

Workflow	Sequential	Parallel
Preprocess (clipOverlap)	2.82	2.91
SNV: mutationSeq	5.68	5.99
SNV: Strelka	2.60	4.49
CNV: Titan	29.34	36.31
CNV: Sequenza	8.24	9.40

## Identifying novel candidate lymphoma-related genes from exome data

**Figure 3:**
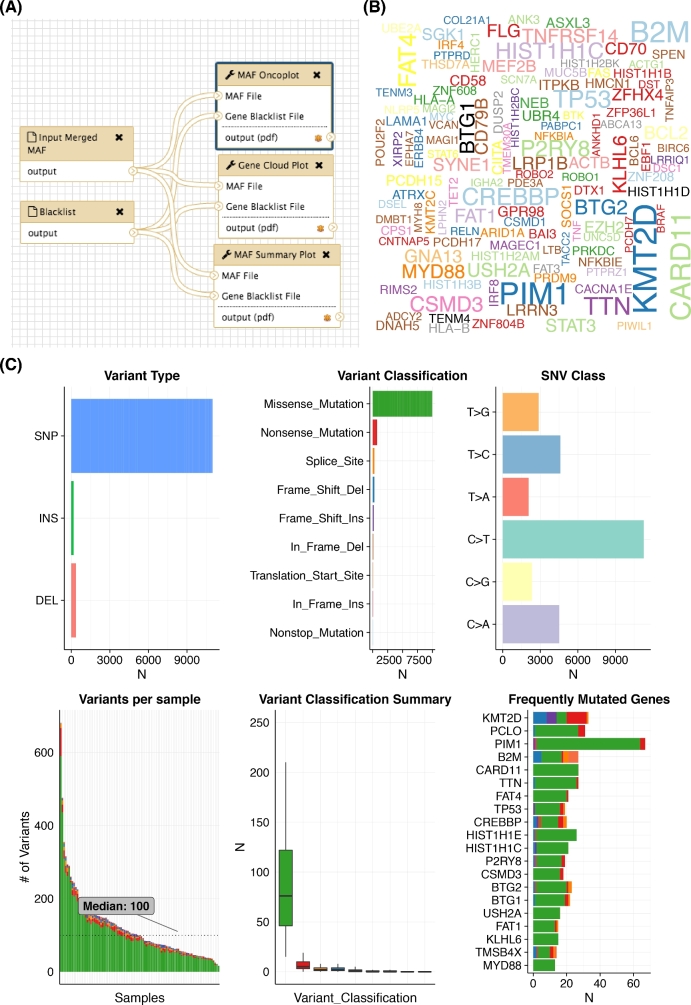
Producing cohort-wide summaries and visualizations. Following primary mutation detection across a large cohort and annotation (i.e., with VEP using vcf2maf), it is useful to produce various summaries of the overall mutation burden and the types and classifications of mutations detected. The maftools R package offers a multitude of visualizations, many of which we have adapted into Galaxy. (**A**) In this example workflow, a merged MAF file containing the variants for the entire cohort of DLBCLs is input alongside a black-list of genes to hide from the outputs. (**B**) This word cloud, generated by the genecloud tool, provides a visually appealing summary of the frequency of mutations in genes above a user-specified threshold. (**C**) A generic mafsummaryplot tool provided by maftools generates six plots that represent descriptive features of the mutations and their annotations. It is evident that C > T is the predominant mutation type detected. A separate tool to perform refined mutation signature analysis is also available. Among the most commonly mutated genes are those previously attributed to DLBCL along with *TTN*, which encodes the largest human protein. With respect to the predicted effect, missense mutations are by far the dominant class of mutations. Despite this, tumor suppressors such as *KMT2D, TP53*, and *B2M* show an elevation of inactivating mutation classes.

An ultimate goal in cancer genome/exome analysis involves the identification of loci recurrently affected by copy number gain or loss and genes recurrently targeted by somatic mutations. There exist myriad tools to detect somatic SNVs and a growing number of options to derive high-quality copy number estimates from genome and exome data. We implemented workflows that perform the required annotation and preprocessing of raw mutation and copy number outputs from tools such as Strelka and Sequenza, respectively. We ran these two workflows on 96 tumor/normal pairs representing DLBCL patients. The SNV and indel calls were annotated and converted to mutation annotation format (MAF) using vcf2maf. A variety of convenient visualization methods are available in the maftools R package [20] and Fig. 3 shows the output of a workflow that employs some of these.

**Figure 4:**
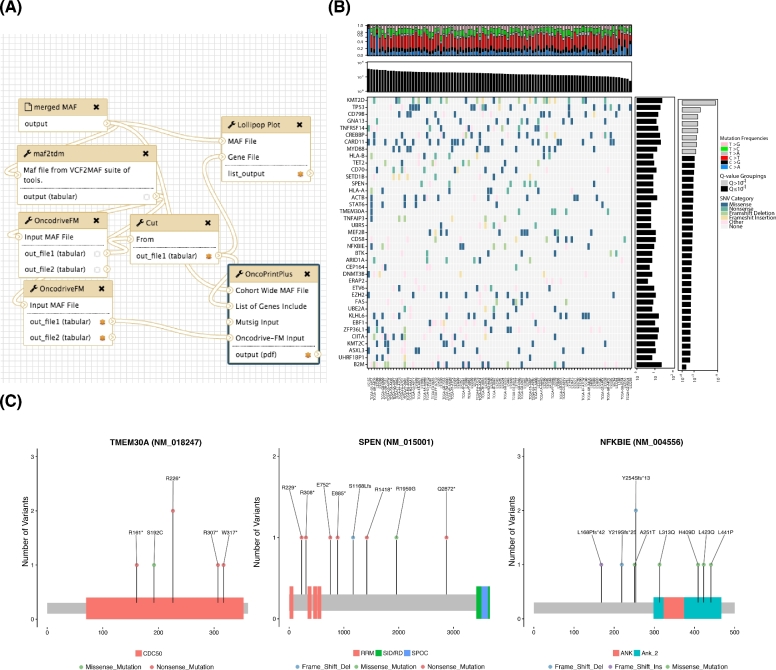
Significance analysis for mutation recurrence. (**A**) Tools have been implemented to screen mutation data for patterns of recurrence and identify significantly mutated genes. Shown above is an example workflow that utilizes the OncodriveFM algorithm and generates various visualizations for genes meeting a pre-specified Q-value cutoff. (**B**) A common approach to summarize mutation data is a two-dimensional matrix with covariates plotted along the side axes. We implemented a tool that leverages multiplot in our R package to generate such images for arbitrary gene lists using the outputs of variant calling workflows that have been annotated using the vcf2maf tool. Mutations are colored based on the severity of mutations assigned automatically by the Ensembl Variant Effect Predictor (VEP) [48]. Genes with more severe mutations are more likely to be tumor suppressor genes (e.g., *B2M* at the bottom and *TP53* and *KMT2D* at the top). Here, the total number of mutations detected in each patient is shown at the top and the *P* value reported by OncodriveFM is shown for each gene is shown on the right. The frequency of each of six possible mutation type can inform on mutational processes in individual samples. This is automatically determined from MAF files and is summarized at the top. (**C**) It is also often desirable to visualize the pattern of mutations within individual genes. The pattern is revealed using the lollipopplot tool that is run on each gene passing the threshold in this workflow.

We next analyzed the pooled mutation calls from the meta-cohort for recurrently mutated genes using OncodriveFM. Fig. 4 shows the workflow that performs these tasks and produces various visualizations of the resulting gene set. A batch tool built on maftools [20] was used to generate protein-centric lollipop plots, which can facilitate visual recognition of patterns indicative of tumor suppressor genes and can also reveal mutation clustering and hot spots (e.g., *TMEM30A* and *NFKBIE*). *TMEM30A* mutations have also been observed previously, although their role remains unclear [21], and based on these visualizations we note a pattern towards protein inactivation and a hot spot in *NFKBIE* that induces a frameshift. The latter was recently observed in a separate set of patients with relapsed DLBCL [22]. *SPEN*, in contrast, has not been reported as recurrent target of somatic mutation in DLBCL but has been found mutated in other lymphoma types. The pattern of mutations suggests it may also act as a tumor suppressor gene in this cancer. We further noted *TET2, SETD1B, ARID1A, UBR5, DNMT3B*, and *BTK* demonstrate similar mutation patterns (Fig. 4B). Although these have been identified as relevant genes in other cancers [23, 24], none of these have, to our knowledge, been previously ascribed to DLBCL.

We next attempted to integrate exome-derived copy number information with mutation calls. Circos is a popular approach to generate visualizations of genome-wide mutation data, although it is generally better suited for genome-wide data and the representation of structural alterations and CNVs relative to genomic coordinates [25]. We extended Circos to generate a gene-centric summarization of SNV and CNV data and produced the Oncocircos Galaxy tool. Rather than plotting on a genomic coordinate scale, gene-level summaries of point mutations are mapped to their relative order on each chromosome and intergenic space (and genes with mutations below the threshold) are eliminated. To accomplish this, we implemented a parser that tabulates the data from MAF and segmented copy number files, applying a threshold to restrict the display to genes with a greater number of mutations cohort-wide. Oncocircos also accepts user-provided gene lists and regions of recurrent CNV (e.g. from GISTIC) and highlights these in the resulting image (Additional Items: Fig. S3). Fig. 6 shows the result of a workflow that runs GISTIC on a merged set of segmented data (in this example, from the Sequenza workflow) and integrates annotated SNV and indel calls from Strelka. In this visualization, several known DLBCL-associated recurrent events are observed including amplifications affecting *REL, MYC*, and *BCL2*, respectively, on 2p, 8q, and 18q. Recurrent deletions affecting the loci containing known tumor suppressor genes are also observable. A complementary visualization of these data is a gene by patient Oncostrip in which annotated copy number and point mutations can be represented (Fig. 7 and Additional Items: Fig. S4).

## Enabling new insights into DLBCL biology

**Figure 5:**
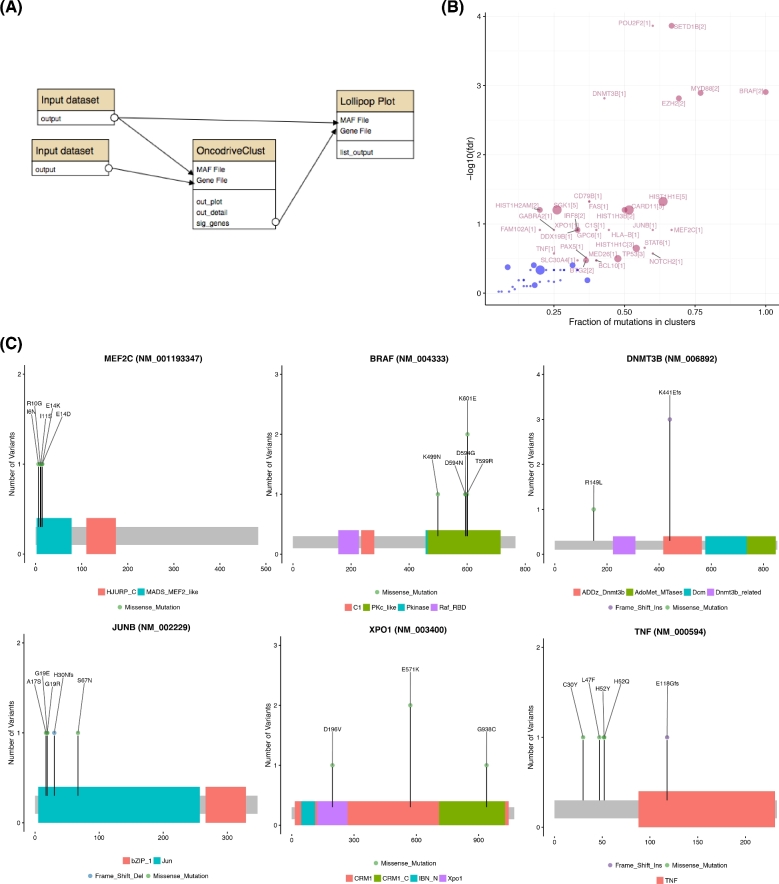
Identifying genes containing clustered mutations and hot spots. With sufficiently large cohorts, the pattern of nonsilent mutations within the protein can inform on genes under specific selective pressure. A clear pattern seen in many dominantly acting cancer genes are mutation hot spots. The OncodriveClust workflow searches for genes with significant clustering of mutations that may represent hot spots or regions/sites whose mutation may produce a dominant effect. Application of this workflow (**A**) detected many lymphoma-related genes known to harbor mutation clusters (**B**). The workflow automatically generates lollipop plots for all genes above a user-specified FDR (in this example, 0.3) (**C**). Clear patterns of hot spots or mutation clusters are visible in each of these genes with only *BRAF* and *MEF2C* having been previously attributed to some DLBCLs [21].

The combined workflows employed here leverage distinct aspects of mutational information that can be individually leveraged to identify candidate cancer drivers and further integrated to inform on disease biology. Using a combination of methods, we provide additional evidence for the importance of several loci that have been attributed to DLBCL with weak support to date and those whose role as an oncogene or tumor suppressor has not been elucidated (Fig. 5). By employing OncodriveClust, we identified genes with significant evidence for mutational recurrence. Mutations around the V600E hot spot in *BRAF* and within *MEF2C* have previously been reported to be present, albeit rare, in DLBCL [19]. Another gene we found to harbor a hot spot was *STAT6*, which, until recently, was thought to be mutated only in some less aggressive lymphomas such as FL and primary mediastinal B-cell lymphoma (PMBCL) [17]. A hot spot mutation in *XPO1* was also observed. This mutation has recently been suggested as a molecular marker of PMBCL distinguishes it from true DLBCLs [26]. One of the two cases bearing the canonical mutation (E571K) was among the few TCGA cases known to be PMBCLs and the other was from the second cohort for which clinical data was unavailable. These observations may further support the presence of mutations that will facilitate detection of PMBCL cases that can be difficult to distinguish from DLBCL by standard clinical criteria.

**Figure 6:**
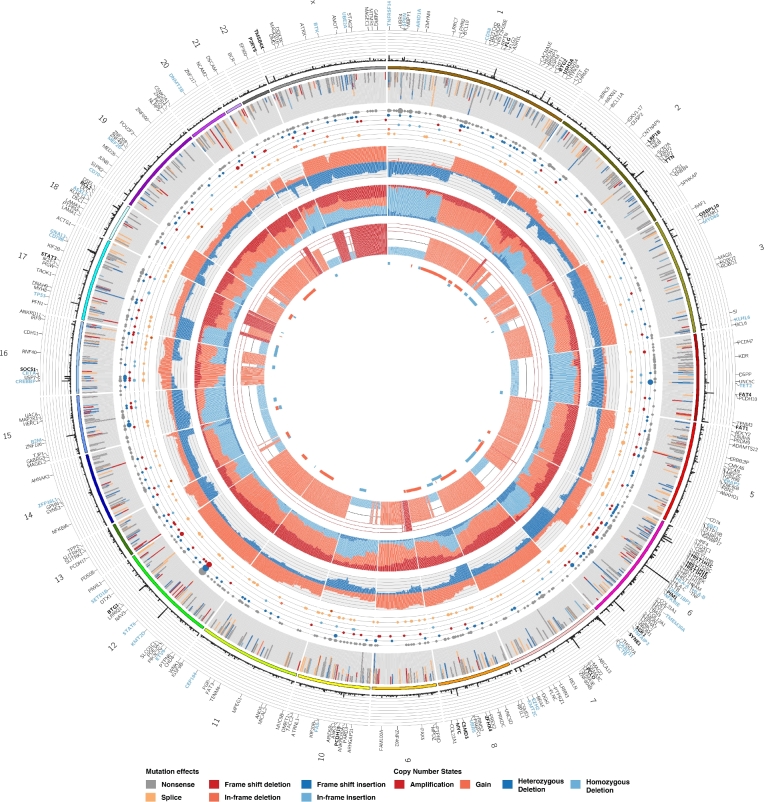
Visualization and data integration with Oncocircos. The new Oncocircos tool allows visualization of segment data derived from the Titan and Sequenza-based workflows we implemented. Genes exceeding a user-specified mutation frequency across the cohort are displayed and labels are automatically added for top genes. Those with at least twice the minimum mutation threshold are labeled in bold and those in an optional user-specified list can also be colored. A black-list file can be optionally provided to hide genes known to be enriched for artefacts. Stacked bar plots and circles provide summary of the annotated SNVs in each gene and a summary of the copy number state of each gene is provided in three inner tracks.

The integration of mutation with copy number data using our tools (Additional Items: Fig. S4) has further informed on the potential relevance of some candidate lymphoma-related genes. *TMEM30A* demonstrated a mutation pattern indicative of tumor suppressor function (Fig. 4C) and inspection of the Oncocircos image (Fig. 6) suggests it resides within the commonly deleted region on 6q. Similarly, *FAT1* appears to have a strong signature towards inactivation and resides in a substantially smaller region that is commonly lost. Such patterns can be more readily confirmed using a separate visualization tool, namely Oncostrip (Additional Items: Figure S4). In contrast, some of the significantly amplified regions of the genome do not appear to harbor genes with significant evidence for recurrent mutations. Amplifications that include *JAK2* are known to be relevant to PMBCL but are not typically considered a feature of DLBCL. Upon inspection of the clinical data available for TCGA cases, we note that each of the four PMBCLs in this cohort contain a mutation or deletion affecting FAT1 and a JAK2 amplification. *POU2AF1*, which resides on 11q23.1, is a candidate target for the amplification of this region despite a low number of nonsilent mutations and has been reported as commonly amplified in treatment-refractory DLBCLs [27]. Further studies that include larger cohorts and possibly whole genome sequence data should help confirm the relevance of these observations.

**Figure 7:**
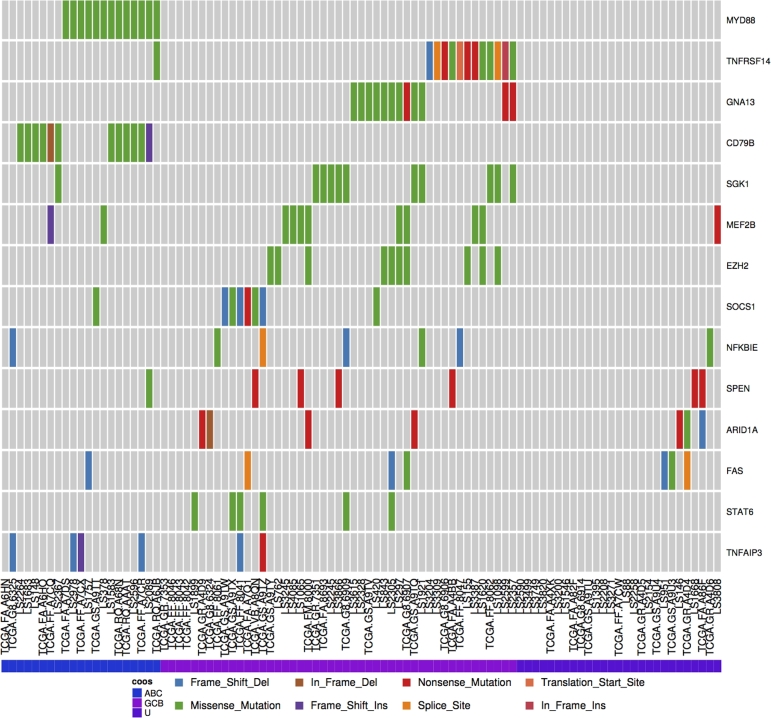
Discerning mutation patterns and identifying subtype-associated genes. DLBCL cases were assigned to either the ABC or GCB molecular subgroups using the presence of mutations known to be significantly restricted to either. Cases with no mutations unique to either molecular subgroup were designated unclassifiable (U).

Many of the genes known to be relevant to DLBCL biology are more commonly mutated in only one of the two molecular subgroups. Fig. 7 shows the mutation distribution across some of these genes in the meta-cohort analyzed here, which has been organized on the predicted subgroup of each patient. Using the Oncostrip tool to order patients on this designation uncovers additional genes in which mutations may be more common in the GCB subgroup such as *NFKBIE, ARID1A, FAS*, and *STAT6* (Additional Items: Fig. S4). *NFKBIE* mutations have recently been reported to be particularly common among PMBCLs and a marker of poor prognosis in that disease [28]. One of the *NFKBIE* mutations detected herein was in a PMBCL case whereas the remainder were in nodal DLBCL cases and was almost exclusively seen in cases with other mutations suggestive of the GCB subgroup. This indicates a potential unappreciated role of *NFKBIE* in DLBCL, or, taken together with our observation of mutations in *STAT6* and *XPO1*, may suggest that a significant subset of PMBCL cases may masquerade as GCB DLBCL. Further refinement of the mutation patterns of the two subgroups of DLBCL and PMBCL using larger cohorts is clearly warranted.

## Towards reproducible and distributable workflows for cancer genome analysis

Large-scale efforts to understand the diversity of cancer-associated somatic alterations across common cancer types are continually expanding in scope. Many such efforts release raw (or aligned) tumor and normal sequence data into controlled-access repositories such as Database of Genotypes and Phenotypes and the European Genome-Phenome Archive. Owing to the many options and variations available in analytical methods, the mutations and copy number results presented along with such data are not directly amenable to direct comparisons between studies or pooled meta-analyses. Instead, the raw data must be obtained and processed uniformly alongside any new data sets. In light of the limited computational resources available to many research labs interested in incorporating existing sequence data into their analyses, some consenting processes and major data repositories are beginning to facilitate storage and processing of patient data using cloud resources.

Our Galaxy Cancer Genomics Toolkit provides a growing list of standard methods for cancer genomic analysis and facilitates their deployment in a simplified, reproducible, and accessible manner using Galaxy, which is amenable to deploying on standalone servers or on a variety of cloud services. We have run our tools and workflows using AWS cloud computing (with CloudMan), which provides a cluster environment to any research lab and on Google Cloud, which facilitates cluster management of Docker-based instances using Kubernetes. We continue to provide new tools by extending functionality and releasing updates. We also note that many of our tools have been tested on whole genome sequence data and additional tools for performing analytical tasks better suited to that data type have been implemented but were not described in detail here. To facilitate scaling of our applications to whole genome and exome data to the extent currently possible in this framework, and to accelerate the analysis of exomes, we established new methods to accomplish parallelization in Galaxy. It is important to note that Galaxy is not particularly well suited to certain large-scale analyses due to how data transfer tasks are handled, the internalization of some processes (e.g., bam indexing) and centralization of intermediate files generated by tools. We hope that ongoing development of the Galaxy codebase will improve on these and suggest that command-line equivalents to our workflows such as those offered by the Kronos software are worthy of consideration in larger-scale projects. Ongoing development of the Galaxy application programming interface may also ameliorate some of these issues.

This toolkit and its ready-made workflows provide the methods essential to drive discovery and eliminate the bottleneck in cancer genomic analysis and templates for creating similar analyses that leverage comparable software. Availability and usability of analytical software are both critical factors in driving their adoption and the ultimate discovery of novel cancer drivers. Accordingly, we provide a series of solutions that should accelerate adoption of our toolkit. First, providing automatic installation for tools wherever possible allow seamless integration into custom Galaxy instances. Second, many of the tools and workflows included here can be optionally configured to efficiently parallelize tasks on a cluster environment. Third, we show that our toolkit can be readily deployed onto a cloud-based Galaxy instance thereby eliminating the need for permanent access to commodity computing hardware or dedicated systems administrators. Together, this offers the potential to enable reproducible cancer research by empowering researchers to perform their own cancer genome analyses with unprecedented accessibility and directly share their workflows such that other groups can reproduce these analyses on additional datasets. We have provided the data files resulting from running each of the workflows described in this manuscript on the DLBCL cohort as examples for users wishing to test these tools.

As ownership of these tools migrates to the Intergalactic Utilities Commission along with transfer to the Main ToolShed, we encourage ongoing testing and parameter optimization and community-driven refinement and expansion of this toolkit. With sufficient adoption and ongoing support, this toolkit could empower numerous groups to explore the many available cancer data sets and their own experimental data using cloud infrastructure thereby facilitating the broader scientific community to make use of the steadily growing genomic resources being produced within this field.

## Abbreviations

AWS Amazon Web Services

CNV copy number variation

DLBCL Diffuse large B-cell lymphoma

GUI graphical user interfaces

PMBCL Primary mediastinal B-cell lymphoma

SNV somatic single nucleotide variant

SV structural variation

TCGA The Cancer Genome Atlas

### Availability and requirements

- Project Name: Cancer Genomics Toolkit for Galaxy
- Project Homepage: https://github.com/morinlab/tools-morinlab
- Operation System: Linux
- Programming language: Python
- Other requirements: Please refer to the source code and the tools-iuc repository.
- License: GPLv3

All tools described herein are available in the Galaxy Test Toolshed (https://testtoolshed.g2.bx.psu.edu/) and under the GPLv3 license via the project GitHub repository. The Dockerfile to automatically install these tools and a prebuilt Docker image are also provided. The dependencies of each tool are documented in the associated tool dependency description and the Dockerfile and are too numerous to detail here. Instead, please refer to the source code: https://github.com/morinlab/tools-morinlab and the tools-iuc repository.

### Availability of supporting data

Archived snapshots of the code and test data are available from the *GigaScience* GigaDB repository [29].

### Competing interests

The authors declare they have no competing interests.

### Author contributions

MAA, SJ, PP, and ER were responsible for deploying tools in Galaxy. MAA and BMG tested workflows on AWS. MAA and MK created figures. MAA, BMG, and RDM wrote the manuscript, which was reviewed and approved by all authors. RDM, PCB, and SPS led the study.

### Additional files

**Additional file Fig. S1** An ensemble approach to detect somatic SNVs. The ensembl_vcf tool receives the output of variant callers and selects variants detected by a user-specified number of tools. This example workflow runs four variant callers (strelka, mutationSeq, RADIA and SomaticSniper) and runs vcf2maf to annotate the resulting list of variants with support from a sufficient number of tools.

**Additional file Fig. S2** Achieving parallelization in Galaxy. There are two ways to achieve parallelization in Galaxy. The first (**A**) employs the parallelism tag, which calls specific split and merge functions depending on the Galaxy input and output data type. These functions are predefined within galaxies codebase. The second (**B**) uses galaxy collections, which are essentially containers of input files. Inputs can be split into a collection of files and subsequently pipeline these through a series of tools. When complete, the individual outputs can be merged. Parallelizing using collections is far more transparent to the user and also limits that number of unnecessary split and merge functions.

**Additional file Fig. S3** A workflow to integrate SNV and CNV data and produce integrative visualizations. This workflow uses exome-derived CNV and SNV data to generate a list of recurrently gained/lost genomic regions (using GISTIC) and displays these along with gene-centric summaries of segmented copy number and SNV data using Oncocircos. To generate Fig. 5, we included a blacklist containing all immunoglobulin genes and Mucin genes whereas the genes identified as significantly mutated by oncodriveFM were provided separately to enforce highlighting.

**Additional file Fig. S4** Using the Oncostrip tool to integrate copy number and mutation data. It can be desirable to visualize the complete set of mutational information cohort-wide without losing the patient-mutation relationships and potential gene-gene interactions that are not retained in Oncocircos. For this application, the Oncostrip component of maftools can also accept raw outputs from GISTIC. Here, we have included the known gene targets of some recurrent amplifications and deletions detected in the cohort (*REL*, B2M, and *TNFRSF14*). Each of *FAT1* and *TMEM30A* reside in significantly deleted regions and bear a combined pattern of mutation and deletion consistent with tumor suppressor function.

**Additional file Table S1** Helper tools implemented to facilitate tool linkage and parallelization.

## Supplementary Material

GIGA-D-16-00157_Original_Submission.pdf

GIGA-D-16-00157_Revision_1.pdf

GIGA-D-16-00157_Revision_2.pdf

Response_to_reviewer_comments_Original_Submission.pdf

Response_to_reviewer_comments_Revision_1.pdf

Reviewer_1_Report_(Original_Submission).pdf

Reviewer_1_Report_(Revision_1).pdf

Reviewer_2_Report_(Original_Submission).pdf

Reviewer_2_Report_(Revision_1).pdf

Supplemental material **Additional file Fig. S1** An ensemble approach to detect somatic SNVs. The ensembl_vcf tool receives the output of variant callers and selects variants detected by a user-specified number of tools. This example workflow runs four variant callers (strelka, mutationSeq, RADIA and SomaticSniper) and runs vcf2maf to annotate the resulting list of variants with support from a sufficient number of tools.
**Additional file Fig. S2** Achieving parallelization in Galaxy. There are two ways to achieve parallelization in Galaxy. The first (**A**) employs the parallelism tag, which calls specific split and merge functions depending on the Galaxy input and output data type. These functions are predefined within galaxies codebase. The second (**B**) uses galaxy collections, which are essentially containers of input files. Inputs can be split into a collection of files and subsequently pipeline these through a series of tools. When complete, the individual outputs can be merged. Parallelizing using collections is far more transparent to the user and also limits that number of unnecessary split and merge functions.
**Additional file Fig. S3** A workflow to integrate SNV and CNV data and produce integrative visualizations. This workflow uses exome-derived CNV and SNV data to generate a list of recurrently gained/lost genomic regions (using GISTIC) and displays these along with gene-centric summaries of segmented copy number and SNV data using Oncocircos. To generate Fig. 5, we included a blacklist containing all immunoglobulin genes and Mucin genes whereas the genes identified as significantly mutated by oncodriveFM were provided separately to enforce highlighting.
**Additional file Fig. S4** Using the Oncostrip tool to integrate copy number and mutation data. It can be desirable to visualize the complete set of mutational information cohort-wide without losing the patient-mutation relationships and potential gene-gene interactions that are not retained in Oncocircos. For this application, the Oncostrip component of maftools can also accept raw outputs from GISTIC. Here, we have included the known gene targets of some recurrent amplifications and deletions detected in the cohort (*REL*, B2M, and *TNFRSF14*). Each of *FAT1* and *TMEM30A* reside in significantly deleted regions and bear a combined pattern of mutation and deletion consistent with tumor suppressor function.
**Additional file Table S1** Helper tools implemented to facilitate tool linkage and parallelization.
