# Supplementary material for: Enhancing knowledge discovery from cancer genomics data with Galaxy
Source: Gigascience. 2017 Mar 9;6(5):1–13. doi: 10.1093/gigascience/gix015 (PMC5437943; doi:10.1093/gigascience/gix015)
Supplement: GIGA-D-16-00157_Original_Submission.pdf [file gix015_GIGA-D-16-00157_Original_Submission.pdf]

Manuscript
Click here to download Manuscript EnhancingKnowledgeDiscoveryfromCancerGenomicsDatawithG

Enhancing Knowledge Discovery from Cancer Genomics Data with Galaxy

Marco A. Albuquerque[1], Bruno M. Grande[1], Elie J. Ritch[1], Prasath Pararajalingam[1], Selin Jessa[1], Martin Krzywinski[2], Jasleen K. Grewal[1], Sohrab P. Shah[3], Paul C. Boutros[4] and Ryan D. Morin[1,2,*]

Author Affiliations

[1]Department of Molecular Biology and Biochemistry, Simon Fraser University, Burnaby, BC, Canada.

[2]Canada's Michael Smith Genome Sciences Center, BC Cancer Agency, Vancouver, BC, Canada.

[3]Department of Pathology, University of British Columbia, Vancouver, BC, Canada.

[4]Ontario Institute for Cancer Research, Toronto, ON, Canada.

Abstract

The field of cancer genomics has demonstrated the power of massively parallel sequencing techniques to inform on the genes and specific alterations that drive tumor onset and progression. Although large comprehensive sequence data sets continue to be made increasingly available, data analysis remains an ongoing challenge, particularly for laboratories lacking dedicated resources and bioinformatics expertise. To address this, we have produced a collection of Galaxy tools that represent many popular algorithms for detecting somatic genetic alterations from cancer genome and exome data. We developed new methods for parallelization of these tools within Galaxy to accelerate runtime and have demonstrated their usability on cloud-based infrastructure and commodity hardware. Some tools represent extensions or refinement of existing toolkits to yield visualizations suited to cohort-wide cancer genomic analysis. For example, we present Oncocircos and Oncoprintplus, which generate data-rich summaries of exome-derived somatic mutation. Workflows that integrate these to achieve data

integration and visualizations are demonstrated on a cohort of 96 diffuse large B-cell lymphomas and enabled the discovery of multiple candidate lymphoma-related genes. Our toolkit is available from our GitHub repository as Galaxy tool and dependency definitions and has been deployed using virtualization on multiple platforms including Docker.

Keywords

Lymphoma; Driver; Cancer; Genome; Pipeline; Workflow; Tool; Cloud;

To whom correspondence should be directed

Dr. Ryan Morin; rdmorin@sfu.ca

Findings

Background

An inherent problem in the application of genomics to understand the molecular aetiology of cancer is the multi-disciplinary skillset required for researchers to draw meaningful inferences from high-throughput biological data. With the rise in popularity of high-throughput DNA sequencing, the bottleneck for discovery has shifted from data generation to data analysis and interpretation. Although myriad algorithms have been developed to efficiently analyze large datasets, these are often tailored for technically inclined users. Software for these analyses is typically run at the command line; operation requires the use of cryptic parameters; efficient parallelization can be difficult to accomplish; and installation is often burdensome. Achieving a flow of data between tools is also often non-trivial and, owing to a paucity of data standards, can involve error-prone data manipulation and re-formatting steps often relying on a collection of custom scripts that are often not released with publications. Combined with a necessity for high-performance computational hardware to run many such tools efficiently, these issues produce a tremendous barrier for novice users.

There exist a handful of options that address this predicament in genomics as a whole. Tools that automate pipeline development such as Kronos [1], Nextflow [2] and Snakemake [3] can satisfy the needs of more technically savvy users. Alternatively, graphical user interfaces (GUIs)—which are generally lacking in the field of bioinformatics—aid in users learning the utility of the associated with command-line interfaces but typically do not scale to large data sets. Examples of genomics tools offering web-accessible GUIs include `BLAST` [4], `VAGUE` [5] and `limmaGUI` [6]. However, beyond an inability to scale, web-based utilities pose several issues, including design inconsistency, redundant efforts in interface development and the inability to automatically link individual tasks into pipelines or workflows. Ideally, any reduction in the barriers associated with running individual algorithms passing data between software tools should accelerate analytical tasks and reduce the risk of errors.

To overcome this, GUI-enabled software for automating pipeline development improves the reproducibility, accessibility and transparency of running genomic analyses [7]. Examples of these include Galaxy [8], Taverna [9], Pegasus [10] and commercial software packages such as Geneious [11]. In particular, the Galaxy project offers many attractive features for this goal while remaining open-source. Namely, Galaxy boasts extensive documentation; support for automatic tool installation; the ability to instantiate public or private “cloud clusters” by leveraging CloudMan [12]; and is as a whole supported by a vibrant community that provides ongoing development to the software. Although algorithms for handling high-throughput sequence data are increasingly being added to Galaxy, there currently remains a lack of tools and workflows tailored to perform common tasks involved in analyzing cancer genome and exome sequence data. Here, we have begun to address this issue by adapting many popular tools for analyzing cancer genome and exome data and made these publicly available as the Galaxy Cancer Genomics Toolkit (GCGT).

Diffuse large B-cell lymphoma (DLBCL) is a common aggressive non-Hodgkin lymphoma that demonstrates extensive genetic heterogeneity with some genetic features found more

common in only one of the two molecular subgroups, namely the ABC and GCB subgroups[13]. Primary mediastinal B-cell lymphoma is defined as a separate entity by the World Health Organization with distinct clinical and diagnostic features but shares some genetic features with DLBCL and other lymphomas[14]. Herein, we demonstrate the utility of the GCGT by applying custom workflows to a large cohort of DLBCL patients (n=96) and through a combination of analytical and exploratory approaches leveraging multiple visualization tools implemented within the Toolkit, we uncover new candidate lymphoma-related genes and putative genetic features associated with each subgroup.

<u>Implementing cancer genomics tools in Galaxy</u>

We produced a comprehensive toolkit comprising a suite of complementary tools and workflows that perform many of the routine analytical tasks in cancer genomics. These include several methods for detecting (“calling”) somatic single nucleotide variants (SNVs), copy number variations (CNVs) and structural variations (SVs) in tumour-normal pairs. Additional tools were developed to perform the many auxiliary steps helper functions that allow these tools to be linked and applied generically, such as bam and text file pre- and post-processing, manipulating and converting file formats; variant annotation; identification of significantly mutated genes; and visualizations for performing exploratory analysis and cohort-level data summarization. The tools and helper functions are briefly detailed in Table 1 and Table S1, respectively with further documentation in our repository.

To integrate individual tools into Galaxy, we implemented XML-based configuration files, which dictate the available inputs and arguments and build the command based on user-specified parameters. Where possible, these contain a consistent design across tools and were developed using Planemo to ensure best practices [15]. All repositories are stored on the public Galaxy test toolshed, which allows users to automatically install any tool [16]. Modular tool dependency repositories provide the step-by-step instructions for Galaxy to automatically

compile necessary software dependencies for each tool. Previously defined repositories were recycled if available. Though we could not successfully produce tools that automatically install on all platforms, many of our tools install (with dependencies) on the standard Galaxy AWS image and in a custom Ubuntu installation (v16.04). Synthetic alignment data containing artificial variants were generated and bundled with variant callers to enable automatic testing [17]. To handle reference data, we implemented new Galaxy data managers and, for simplicity, allow the option of user-provided reference data [18].

Support within Galaxy for processing large data sets is still being established and one remaining restriction has been the lack of methods for splitting and parallelizing large analyses. We invested substantial effort to ensure that tools are parallelizable on cluster environments wherever it was deemed desirable and possible. Following the addition of new data types in the Galaxy codebase, this was subsequently re-implemented using the more transparent and efficient method that exploits the more recent Galaxy feature known as “data collections”. Briefly, parallelization of a workflow is accomplished by a combination of tasks (Figure 1), beginning with `fetch_interval`. This obtains chromosomal information from each input read alignment file and creates a collection of BED files defining all intervals available to each tool. To balance the load across all concurrently spawned jobs, we automatically pair large and small intervals. The second stage is a `preprocess` tool which defines all necessary preprocessing steps in a single tool and all will be executed together to reduce the numerous outputs associated with running multiple separate preprocessing tools in Galaxy. This includes a `samtools` flag and mapping quality filter, `samtools` remove duplicates and `bamutils clipoverlap`. The third stage involves running the selected tool on each of the intervals, allowing Galaxy to spawn processes to available CPUs. The fourth stage is `postprocess`, which follows similar methodology to `preprocess`. Example usage includes further variant filtration and annotation steps specific to an individual tool. Finally, all output files are merged, if

necessary, so they may be supplied to subsequent tools and workflows. For software that can be multithreaded, we instead leverage this capability rather than chromosomal splitting.

Many of the analyses and workflows shown in detail here were performed on a local Galaxy instance on a linux-based server. For computationally demanding tasks, we launched a Galaxy instance on AWS Elastic Cloud Compute (EC2) using CloudMan and installed the workflow and tool dependencies. A cluster configuration consisting of one r3.8xlarge master node and five r3.2xlarge worker nodes was selected. We uploaded 96 bam files representing the cohort of published DLBCL samples and ran all SNV and CNV workflows on this configuration for each tumour/normal pair and captured details on runtime and speedup associated with parallelization (not shown) [19]. Separately, we produced a Dockerfile that will install our tools and additional dependencies using the galaxy-stable Docker image (https://hub.docker.com/r/bgruening/galaxy-stable/~/dockerfile/). This is available in the GitHub repository that hosts the individual tools.

Selecting high-value tools and developing workflows for routine analytical tasks

There are numerous algorithms available to perform standard analytical tasks such as variant calling and CNV detection, each offering different balances of usability, computational efficiency and accuracy. As such, selection of ideal tools and parameters is non-trivial. We implemented tools representing some of the more commonly cited options and include many that performed favorably in ICGC-TCGA DREAM challenges [20]. As each can be configured with a number of parameters, which can be tuned for improved accuracy, we leverage results from the DREAM challenge to assist in selecting the more accurate algorithms and in setting sensible default parameters [21]. Because ensemble approaches tend to provide increased accuracy, we also created a tool to integrate variant calls from multiple algorithms using a simple voting scheme (Additional Items: Figure S1). We have also released numerous workflows that run some of the more complicated pieces of software that relies on many

dependencies and that perform some routine analytical and visualization tasks as detailed and illustrated with the real-world worked examples below. Example workflows that demonstrate our new approach to perform parallelization in Galaxy are included in Figure 1 and Additional items: Figure S2.

<u>Identifying novel candidate lymphoma-related genes from exome data</u>

An ultimate goal in cancer genome/exome analysis involves the identification of loci recurrently affected by copy number gain or loss and genes recurrently targeted by somatic mutations. There exist myriad tools to detect somatic SNVs and a growing number of options to derive high-quality copy number estimates from genome and exome data. We implemented workflows that perform the required annotation and pre-processing of raw mutation and copy number outputs from tools such as `Strelka` and `Sequenza`, respectively. We ran these two workflows on 96 tumor/normal pairs representing DLBCL patients. The SNV and indel calls were annotated and converted to mutation annotation format (MAF) using `vcf2maf`. A variety of convenient visualization methods are available in the `maftools` R package [22] and Figure 2 shows the output of a workflow that employs some of these.

We next analyzed the pooled mutation calls from the meta-cohort for recurrently mutated genes using `OncodriveFM`. Figure 3 shows the workflow that performs these tasks and produces various visualizations of the resulting gene set. A batch tool built on `maftools` [22] was used to generate protein-centric lollipop plots, which can facilitate visual recognition of patterns indicative of tumor suppressor genes and can also reveal mutation clustering and hot spots (e.g. *TMEM30A* and *NFKBIE*). *TMEM30A* mutations have also been observed previously although their role remains unclear [16] and based on these visualizations we note a pattern towards protein inactivation and we also see a clear hot spot in *NFKBIE* that induces a frameshift. The latter was recently observed in a separate set of patients with relapsed DLBCL

[23]. *SPEN*, in contrast, has not been reported as recurrent target of somatic mutation in DLBCL but has been found mutated in other lymphoma types. The pattern of mutations suggests it may also act as a tumor suppressor gene in this cancer. We further noted *TET2*, *SETD1B*, *ARID1A*, *UBR5*, *DNMT3B* and *BTK* demonstrate similar mutation patterns (Figure 3B). Although these have been identified as relevant genes in other cancers [24],[25], none of these have, to our knowledge, been previously ascribed to DLBCL.

We next attempted to integrate exome-derived copy number information with mutation calls. `Circos` is a popular approach to generate visualizations of genome-wide mutation data although it is generally better suited for genome-wide data and the representation of structural alterations and CNVs relative to genomic coordinates [26]. We extended `Circos` to generate a gene-centric summarization of SNV and CNV data and produced the `Oncocircos` Galaxy tool. Rather than plotting on a genomic coordinate scale, gene-level summaries of point mutations are mapped to their relative order on each chromosome and intergenic space (and genes with mutations below the threshold) are eliminated. To accomplish this, we implemented a parser that tabulates the data from MAF and segmented copy number files, applying a threshold to restrict the display to genes with a greater number of mutations cohort-wide. `Oncocircos` also accepts user-provided gene lists and regions of recurrent CNV (e.g. from `GISTIC`) and highlights these in the resulting image (Additional Items: Figure S3). Figure 5 shows the result of a workflow that runs `GISTIC` on a merged set of segmented data (in this example, from the `Sequenza` workflow) and integrates annotated SNV and indel calls from `Strelka`. In this visualization, several known DLBCL-associated recurrent events are observed including amplifications affecting *REL*, *MYC* and *BCL2* respectively on 2p, 8q and 18q. Recurrent deletions affecting the loci containing known tumor suppressor genes are also observable. A complementary visualization of these data is a gene by patient `Oncostrip` in which annotated

copy number and point mutations can be represented (Figure 6 and Additional Items: Figure S4).

Enabling new insights into DLBCL biology

The combined workflows employed here leverage distinct aspects of mutational information that can be individually leveraged to identify candidate cancer drivers and further integrated to inform on disease biology. Using a combination of methods, we provide additional evidence for the importance of several that have been attributed to DLBCL with weak support to date and those whose role as an oncogene or tumor suppressor has not been elucidated. By employing `OncodriveClust`, we identified several genes with significant evidence for mutational recurrence. Mutations around the V600E hot spot in *BRAF* and within *MEF2C* have previously been reported to be present, albeit rare, in DLBCL [19]. Another mutation we found to harbor a hot spot was STAT6 which, until recently, was thought to be mutated only in some less aggressive lymphomas such as FL and primary mediastinal B-cell lymphoma (PMBCL)[17]. A hot spot mutation in *XPO1* was also observed here. This mutation has recently been suggested as a molecular marker of PMBCL distinguishes it from true DLBCLs [27]. One of the two cases bearing the canonical mutation (E571K) was among the few TCGA cases known to be PMBCLs and the other was from the second cohort for which clinical data was unavailable. These observations may further support the presence of mutations that will facilitate detection of PMBCL cases that can be difficult to distinguish from DLBCL.

The integration of mutation with copy number data using our tools (Additional Item 4) has further informed on the potential relevance of some candidate lymphoma-related genes. *TMEM30A* demonstrated a mutation pattern indicative of tumor suppressor function (Figure 3C) and inspection of the `Oncocircos` image suggests it resides within the commonly deleted region on 6q. Similarly, *FAT1* appears to have a strong signature towards inactivation and resides in a substantially smaller region that is commonly lost. Such patterns can be more

readily confirmed using a separate visualization tool, namely `Oncostrip` (Additional Items: Figure S4). In contrast, some of the significantly amplified regions of the genome do not appear to harbor genes with significant evidence for recurrent mutations. Amplifications that include *JAK2* are known to be relevant to PMBCL but are not typically considered a feature of DLBCL. Upon inspection of the clinical data available for TCGA cases, we note that each of the four PMBCLs in this cohort contain a mutation or deletion affecting FAT1 and a JAK2 amplification. *POU2AF1*, which resides on 11q23.1, is a candidate target for the amplification of this region despite a low number of non-silent mutations and has been reported as commonly amplified in treatment-refractory DLBCLs [28]. Further studies that include larger cohorts and possibly whole genome sequence data should help confirm the relevance of these observations.

Many of the genes known to be relevant to DLBCL biology are more commonly mutated in only one of the two molecular subgroups. Figure 6 shows the mutation distribution across some of these genes in the meta-cohort analyzed here, which has been organized on the predicted subgroup of each patient. Using the `Oncostrip` tool to order patients on this designation uncovers additional genes in which mutations may be more common in the GCB subgroup such as *NFKBIE*, *ARID1A*, *FAS* and *STAT6*. *NFKBIE* mutations have recently been reported to be particularly common among PMBCLs and a marker of poor prognosis in that disease (Additional Items: Figure S4)[29]. One of the *NFKBIE* mutations detected herein was in a PMBCL case whereas the remainder were in nodal DLBCL cases and was almost exclusively seen in cases with other mutations suggestive of the GCB subgroup. This indicates a potential unappreciated role of *NFKBIE* in DLBCL or, taken together with our observation of mutations in *STAT6* and *XPO1*, may suggest that a significant subset of PMBCL cases may masquerade as GCB DLBCL. Further refinement of the mutation patterns of the two subgroups of DLBCL and PMBCL using larger cohorts is clearly warranted.

Towards reproducible and distributable workflows for cancer genome analysis

Large-scale efforts to understand the diversity of cancer-associated somatic alterations across common cancer types are continually expanding in scope. Many such efforts release raw (or aligned) tumor and normal sequence data into controlled-access repositories such as dbGAP and the European Genome-Phenome Archive (EGA). Owing to the many options and variations available in analytical methods, the mutations and copy number results presented along with these data are not directly amenable to direct comparisons between studies or pooled meta-analyses. Instead, the raw data must be obtained and processed uniformly alongside any new data sets. In light of the limited computational resources available to many research labs interested in incorporating existing sequence data into their analyses, some consenting processes and major data repositories are beginning to facilitate storage and processing of patient data using cloud resources.

Our Galaxy Cancer Genomics Toolkit provides a growing list of standard methods for cancer genomic analysis and facilitates their deployment in a simplified, reproducible and accessible manner using Galaxy, which is amenable to deploying on standalone servers or on a variety of cloud services. We have successfully run our tools and workflows using AWS cloud computing and CloudMan, which provides a cluster environment to any research lab. We continue to provide new tools by extending functionality and updating versions as algorithms are refined. We also note that many of our tools have also been tested on whole genome sequence data and additional tools for performing analytical tasks better suited to that data type have been implemented but were not described in detail here. To facilitate scaling of these applications to such applications and to accelerate the analysis of exomes, we established new methods to accomplish parallelization in Galaxy.

The release of this suite of tools provides the methods essential to drive discovery and eliminate the bottleneck in cancer genomic analysis. Availability and usability of analytical software are both critical factors in driving their adoption and the ultimate discovery of novel cancer drivers. Accordingly, we provide a series of solutions that should accelerate adoption of

our toolkit. First, providing automatic installation for tools wherever possible allow seamless integration into custom Galaxy instances. Second, many of the tools and workflows included here can be optionally configured to efficiently parallelize tasks on a cluster environment. Third, we show that our toolkit can be readily deployed onto a cloud-based Galaxy instance thereby eliminating the need for permanent access to commodity computing hardware. Together, this offers the potential to enable reproducible cancer research by empowering researchers to perform their own cancer genome analyses with unprecedented accessibility and directly share their workflows such that other groups can reproduce these analyses on additional datasets. As these migrate to the main Galaxy tool shed, we encourage ongoing testing and parameter optimization and community-driven refinement and expansion of this toolkit. With sufficient adoption and ongoing support, this could help empower numerous groups to explore the many available cancer data sets and their own experimental data using cloud infrastructure thereby fostering uptake of the steadily growing genomic resources being produced within this field to inform more broadly on cancer research.

Availability

All tools described herein are available in the Galaxy Test Toolshed and under the GPLv3 license via the project GitHub repository. The Dockerfile to automatically install these tools is also provided. The dependencies of each tool are documented in the associated tool dependency description and the Dockerfile and are too numerous to detail here.
https://github.com/morinlab/tools-morinlab

Competing Interests

The authors declare they have no competing interests.

Authors' Contributions

M.A.A., S.J., P.P, and E.R. were responsible for deploying tools in galaxy. M.A.A. and B.M.G. tested workflows on AWS. M.A.A. and M.K. created figures. M.A.A., B.M.G. and R.D.M. wrote the manuscript, which was reviewed and approved by all authors. R.D.M, P.C.B. and S.P.S. led the study.

Acknowledgements

The results published here are in whole or part based upon data generated by the TCGA Research Network: http://cancergenome.nih.gov/. We gratefully acknowledge TCGA and all providers of samples and resources for generating this valuable resource. The TCGA exome data was obtained through dbGAP (phs000178.v9.p8 and phs000450.v2.p1) and the latter has been described previously [[19]]. Said data was produced as part of the Slim Initiative for Genomic Medicine (SIGMA), a joint U.S.-Mexico project funded by the Carlos Slim Health Institute. This work was supported by a contract from Genome Canada and Genome British Columbia (173CIC), funding from Mitacs (awarded to Morin) and Amazon AWS research grant. Sequencing of the large DLBCL cohort was funded by an operating grant from CIHR (to RDM). RDM is supported by New Investigator Awards from the Canadian Institutes for Health Research and the Terry Fox Research Institute. We thank all members of the Boutros, Shah and Morin research groups for feedback on this work. We also thank the Galaxy community for their ongoing support. We are particularly grateful to Enis Afgan, John Chilton, Nitesh Turaga and Björn Gruening for their gracious assistance. We also thank Marija Jovanovic for assisting in tool deployment.

Table 1: Main tools currently comprising the cancer genomics toolkit.

Tool	Category	Reference
mutationSeq	SNV detection	[30]
Strelka	SNV and indel detection	[31]
SomaticSniper	SNV detection	[32]
RADIA	SNV detection	[33]
VarDict (Java)	SNV detection	[34]
DELLY	SV detection	[35]
LUMPY	SV detection	[36]
Pindel	SV and indel detection	[37]
Manta	SV detection	[38]
Sequenza	CNV detection	[39]
TITAN	CNV detection	[40]
Ensembl VEP	SNV Annotation	[41]
PyClone	Clonal structure	[42]
EXPANDS	Clonal structure	[43]
MutSigCV	Significantly Mutated Genes	[44]
Oncodrive-FM	Significantly Mutated Genes	[45]
GISTIC	Significantly Mutated Genes	[46]
Maftools (oncostrip, oncodriveclust,	Visualization, significantly	[22]
Oncocircos	Visualization	[26]
Oncoprintplus	Visualization	[47]

Tools representing existing or extended analysis approaches are shown above. For a current list of tools available, refer to the repository.

Figure 1. Parallelization in variant calling and other CPU-intensive processes.

(A) An alignment file flows through to `fetch_interval`, which obtains all contigs in an alignment file. If parallelization is requested, multiple interval files are generated for each interval, otherwise a single file is created. Each dataset in the collection is treated as separate input to two instance of `preprocess`, which filters reads from the sequence alignment file for normal and tumour alignment files. These then pass to the variant caller. A `postprocess` tool filters and annotates variant calls based on tool-specific parameters and all final variants are merged and sorted in a single variant file. (B) We perform automatic interval selection to roughly balance the load on each variant-calling task. The algorithm combines regions (e.g. chromosomes) if their total length is less than the largest. In cloud-based settings, this reduces overhead associated with creating multiple unnecessary parallelized jobs as well as reducing the number of short-lived automatically added nodes. Importantly, we chose not to implement a sub-chromosomal interval selection algorithm to maintain intrachromosomal dependence required by some of the variant calling algorithms. Such an extension could be implemented for tools that lack this restriction.

Figure 2. Producing cohort-wide summaries and visualizations.

Following primary mutation detection across a large cohort and annotation (i.e. with VEP using `vcf2maf`), it is useful to produce various summaries of the overall mutation burden and the types and classifications of mutations detected. The `maftools` R package offers a multitude of visualizations, many of which we have adapted into Galaxy. (A) In this example workflow, a merged MAF file containing the variants for the entire cohort of DLBCLs is input alongside a black-list of genes to hide from the outputs. (B) This word cloud, generated by the `genecloud` tool, provides a visually appealing summary of the frequency of mutations in genes above a user-specified threshold. (C) A generic `mafsummaryplot` tool provided by `maftools`

generates six plots that represent descriptive features of the mutations and their annotations. It is evident that C>T is the predominant mutation type detected. A separate tool to perform refined mutation signature analysis is also available. Among the most commonly mutated genes are those previously attributed to DLBCL along with *TTN*, which encodes the largest human protein. With respect to the predicted effect, missense mutations are by far the dominant class of mutations. Despite this, tumor suppressors such as *KMT2D*, *TP53* and *B2M* show an elevation of inactivating mutation classes.

Figure 3. Significance analysis for mutation recurrence.

(A) Tools have been implemented to screen mutation data for patterns of recurrence and identify significantly mutated genes. Shown above is an example workflow that utilizes the `OncodriveFM` algorithm and generates various visualizations for genes meeting a pre-specified Q-value cutoff. (B) A common approach to summarize mutation data is a two-dimensional matrix with covariates plotted along the side axes. We implemented a tool that leverages `multiplot` in our R package to generate such images for arbitrary gene lists using the outputs of variant calling workflows that have been annotated using the `vcf2maf` tool. Mutations are colored based on the severity of mutations assigned automatically by the Ensembl Variant Effect Predictor (VEP) [[48]]. Genes with more severe mutations are more likely to be tumor suppressor genes (e.g. *B2M* at the bottom and *TP53* and *KMT2D* at the top). Here, the total number of mutations detected in each patient is shown at the top and the P-value reported by `OncodriveFM` is shown for each gene is shown on the right. The frequency of each of six possible mutation type can inform on mutational processes in individual samples. This is automatically determined from MAF files and is summarized at the top. (C) It is also often desirable to visualize the pattern of mutations within individual genes. The pattern is revealed using the `lollipopplot` tool that is run on each gene passing the threshold in this workflow.

Figure 4. Identifying genes containing clustered mutations and hot spots.

With sufficiently large cohorts, the pattern of non-silent mutations within the protein can inform on genes under specific selective pressure. A clear pattern seen in many dominantly acting cancer genes are mutation hot spots. The `OncodriveClust` workflow searches for genes with significant clustering of mutations that may represent hot spots or regions/sites whose mutation may produce a dominant effect. Application of this workflow (A) detected many lymphoma-related genes known to harbor mutation clusters (B). The workflow automatically generates lollipop plots for all genes above a user-specified FDR (in this example, 0.3) (C). Clear patterns of hot spots or mutation clusters are visible in each of these genes with only *BRAF* and *MEF2C* having been previously attributed to some DLBCLs [[19]].

Figure 5. Visualization and data integration with `Oncocircos`.

The new `Oncocircos` tool allows visualization of segment data derived from the Titan and `Sequenza`-based workflows we implemented. Genes exceeding a user-specified mutation frequency across the cohort are displayed and labels are automatically added for top genes. Those with at least twice the minimum mutation threshold are labeled in bold and those in an optional user-specified list can also be colored. A black-list file can be optionally provided to hide genes known to be enriched for artefacts. Stacked bar plots and circles provide summary of the annotated SNVs in each gene and a summary of the copy number state of each gene is provided in three inner tracks.

Figure 6. Discerning mutation patterns and identifying subtype-associated genes.

DLBCL cases were assigned to either the ABC or GCB molecular subgroups using the presence of mutations known to be significantly restricted to either. Cases with no mutations unique to either molecular subgroup were designated unclassifiable (U).

References

1. Jafar Taghiyar M, Rosner J, Grewal D, Grande B, Aniba R, Grewal J, Boutros PC, Morin RD, Bashashati A, Shah SP: **Kronos: a workflow assembler for genome analytics and informatics**. *bioRxiv* 2016:040352.

2. Kurs JP, Simi M, Campagne F: *Nextflow Workbench Documentation Booklet*. Fabien Campagne; 2015.

3. Köster J, Rahmann S: **Snakemake--a scalable bioinformatics workflow engine**. *Bioinformatics* 2012, **28**:2520–2522.

4. Altschul SF, Madden TL, Schäffer AA, Zhang J, Zhang Z, Miller W, Lipman DJ: **Gapped BLAST and PSI-BLAST: a new generation of protein database search programs**. *Nucleic Acids Res* 1997, **25**:3389–3402.

5. Powell DR, Seemann T: **VAGUE: a graphical user interface for the Velvet assembler**. *Bioinformatics* 2013, **29**:264–265.

6. Wettenhall JM, Smyth GK: **limmaGUI: a graphical user interface for linear modeling of microarray data**. *Bioinformatics* 2004, **20**:3705–3706.

7. Goecks J, Nekrutenko A, Taylor J, Galaxy Team: **Galaxy: a comprehensive approach for supporting accessible, reproducible, and transparent computational research in the life sciences**. *Genome Biol* 2010, **11**:R86.

8. Goecks J, Nekrutenko A, Taylor J, Galaxy Team: **Galaxy: a comprehensive approach for supporting accessible, reproducible, and transparent computational research in the life sciences**. *Genome Biol* 2010, **11**:R86.

9. Zhang H, Hyde Z, Stian S-R, Carole G: **Taverna Mobile: Taverna workflows on Android**. *EMBnet.journal* 2013, **19**(B):43.

10. Deelman E, Ewa D, Karan V, Gideon J, Mats R, Scott C, Maechling PJ, Rajiv M, Weiwei C, da Silva RF, Miron L, Kent W: **Pegasus, a workflow management system for science automation**. *Future Gener Comput Syst* 2015, **46**:17–35.

11. Kearse M, Moir R, Wilson A, Stones-Havas S, Cheung M, Sturrock S, Buxton S, Cooper A, Markowitz S, Duran C, Thierer T, Ashton B, Meintjes P, Drummond A: **Geneious Basic: an integrated and extendable desktop software platform for the organization and analysis of sequence data**. *Bioinformatics* 2012, **28**:1647–1649.

12. Afgan E, Baker D, Coraor N, Chapman B, Nekrutenko A, Taylor J: **Galaxy CloudMan: delivering cloud compute clusters**. *BMC Bioinformatics* 2010, **11 Suppl 12**:S4.

13. Morin RD, Mendez-Lago M, Mungall AJ, Goya R, Mungall KL, Corbett RD, Johnson NA, Severson TM, Chiu R, Field M, Jackman S, Krzywinski M, Scott DW, Trinh DL, Tamura-Wells J, Li S, Firme MR, Rogic S, Griffith M, Chan S, Yakovenko O, Meyer IM, Zhao EY, Smailus D, Moksa M, Chittaranjan S, Rimsza L, Brooks-Wilson A, Spinelli JJ, Ben-Neriah S, et al.:

Frequent mutation of histone-modifying genes in non-Hodgkin lymphoma. *Nature* 2011, **476**:298–303.

14. Bea S, Zettl A, Wright G, Salaverria I, Jehn P, Moreno V, Burek C, Ott G, Puig X, Yang L, Lopez-Guillermo A, Chan WC, Greiner TC, Weisenburger DD, Armitage JO, Gascoyne RD, Connors JM, Grogan TM, Braziel R, Fisher RI, Smeland EB, Kvaloy S, Holte H, Delabie J, Simon R, Powell J, Wilson WH, Jaffe ES, Montserrat E, Muller-Hermelink H-K, et al.: **Diffuse large B-cell lymphoma subgroups have distinct genetic profiles that influence tumor biology and improve gene-expression-based survival prediction**. *Blood* 2005, **106**:3183–3190.

15. **galaxyproject/planemo** [https://github.com/galaxyproject/planemo]

16. Blankenberg D, Von Kuster G, Bouvier E, Baker D, Afgan E, Stoler N, Galaxy Team, Taylor J, Nekrutenko A: **Dissemination of scientific software with Galaxy ToolShed**. *Genome Biol* 2014, **15**:403.

17. Ewing AD, Houlahan KE, Hu Y, Ellrott K, Caloian C, Yamaguchi TN, Bare JC, P'ng C, Waggott D, Sabelnykova VY, ICGC-TCGA DREAM Somatic Mutation Calling Challenge participants, Kellen MR, Norman TC, Haussler D, Friend SH, Stolovitzky G, Margolin AA, Stuart JM, Boutros PC: **Combining tumor genome simulation with crowdsourcing to benchmark somatic single-nucleotide-variant detection**. *Nat Methods* 2015, **12**:623–630.

18. Blankenberg D, Johnson JE, Galaxy Team, Taylor J, Nekrutenko A: **Wrangling Galaxy's reference data**. *Bioinformatics* 2014, **30**:1917–1919.

19. Lohr JG, Stojanov P, Lawrence MS, Auclair D, Chapuy B, Sougnez C, Cruz-Gordillo P, Knoechel B, Asmann YW, Slager SL, Novak AJ, Dogan A, Ansell SM, Link BK, Zou L, Gould J, Saksena G, Stransky N, Rangel-Escareño C, Fernandez-Lopez JC, Hidalgo-Miranda A, Melendez-Zajgla J, Hernández-Lemus E, Schwarz-Cruz y Celis A, Imaz-Rosshandler I, Ojesina AI, Jung J, Pedamallu CS, Lander ES, Habermann TM, et al.: **Discovery and prioritization of somatic mutations in diffuse large B-cell lymphoma (DLBCL) by whole-exome sequencing**. *Proc Natl Acad Sci U S A* 2012, **109**:3879–3884.

20. Boutros PC, Ewing AD, Ellrott K, Norman TC, Dang KK, Hu Y, Kellen MR, Suver C, Bare JC, Stein LD, Spellman PT, Stolovitzky G, Friend SH, Margolin AA, Stuart JM: **Global optimization of somatic variant identification in cancer genomes with a global community challenge**. *Nat Genet* 2014, **46**:318–319.

21. Ewing AD, Houlahan KE, Hu Y, Ellrott K, Caloian C, Yamaguchi TN, Bare JC, P'ng C, Waggott D, Sabelnykova VY, ICGC-TCGA DREAM Somatic Mutation Calling Challenge participants, Kellen MR, Norman TC, Haussler D, Friend SH, Stolovitzky G, Margolin AA, Stuart JM, Boutros PC: **Combining tumor genome simulation with crowdsourcing to benchmark somatic single-nucleotide-variant detection**. *Nat Methods* 2015, **12**:623–630.

22. Mayakonda A, Phillip Koeffler H: **Maftools: Efficient analysis, visualization and summarization of MAF files from large-scale cohort based cancer studies**. *bioRxiv* 2016:052662.

23. Morin RD, Assouline S, Alcaide M, Mohajeri A, Johnston RL, Chong L, Grewal J, Yu S, Fornika D, Bushell K, Nielsen TH, Petrogiannis-Haliotis T, Crump M, Tosikyan A, Grande BM, MacDonald D, Rousseau C, Bayat M, Sesques P, Froment R, Albuquerque M, Monczak Y,

Oros KK, Greenwood C, Riazalhosseini Y, Arseneault M, Camlioglu E, Constantin A, Pan-Hammarstrom Q, Peng R, et al.: **Genetic Landscapes of Relapsed and Refractory Diffuse Large B-Cell Lymphomas**. *Clin Cancer Res* 2016, **22**:2290–2300.

24. Meissner B, Kridel R, Lim RS, Rogic S, Tse K, Scott DW, Moore R, Mungall AJ, Marra MA, Connors JM, Steidl C, Gascoyne RD: **The E3 ubiquitin ligase UBR5 is recurrently mutated in mantle cell lymphoma**. *Blood* 2013, **121**:3161–3164.

25. Wiegand KC, Shah SP, Al-Agha OM, Zhao Y, Tse K, Zeng T, Senz J, McConechy MK, Anglesio MS, Kalloger SE, Yang W, Heravi-Moussavi A, Giuliany R, Chow C, Fee J, Zayed A, Prentice L, Melnyk N, Turashvili G, Delaney AD, Madore J, Yip S, McPherson AW, Ha G, Bell L, Fereday S, Tam A, Galletta L, Tonin PN, Provencher D, et al.: **ARID1A mutations in endometriosis-associated ovarian carcinomas**. *N Engl J Med* 2010, **363**:1532–1543.

26. Krzywinski M, Schein J, Birol I, Connors J, Gascoyne R, Horsman D, Jones SJ, Marra MA: **Circos: an information aesthetic for comparative genomics**. *Genome Res* 2009, **19**:1639–1645.

27. Jardin F, Pujals A, Pelletier I, Others: **Whole exome sequencing of refractory aggressive B-cell lymphomas identified recurrent mutations of the exportin 1 gene (XPO1) in primary mediastinal B-cell lymphoma subtype, a LYSA study**. *Hematol Oncol* 2015, **33**(suppl 1):100–180.

28. Park HY, Lee S-B, Yoo H-Y, Kim S-J, Kim W-S, Kim J-I, Ko Y-H: **Whole-exome and transcriptome sequencing of refractory diffuse large B-cell lymphoma**. *Oncotarget* 2016.

29. Mansouri L, Noerenberg D, Young E, Mylonas E, Abdulla M, Frick M, Asmar F, Ljungström V, Schneider M, Yoshida K, Skaftason A, Pandzic T, Gonzalez B, Tasidou A, Waldhueter N, Rivas-Delgado A, Angelopoulou M, Ziepert M, Arends CM, Couronné L, Lenze D, Baldus CD, Bastard C, Okosun J, Fitzgibbon J, Dörken B, Drexler HG, Roos-Weil D, Schmitt CA, Munch-Petersen HD, et al.: **Frequent NFKBIE deletions are associated with poor outcome in primary mediastinal B-cell lymphoma**. *Blood* 2016.

30. Ding J, Bashashati A, Roth A, Oloumi A, Tse K, Zeng T, Haffari G, Hirst M, Marra MA, Condon A, Aparicio S, Shah SP: **Feature-based classifiers for somatic mutation detection in tumour-normal paired sequencing data**. *Bioinformatics* 2012, **28**:167–175.

31. Saunders CT, Wong WSW, Swamy S, Becq J, Murray LJ, Cheetham RK: **Strelka: accurate somatic small-variant calling from sequenced tumor-normal sample pairs**. *Bioinformatics* 2012, **28**:1811–1817.

32. Larson DE, Harris CC, Chen K, Koboldt DC, Abbott TE, Dooling DJ, Ley TJ, Mardis ER, Wilson RK, Ding L: **SomaticSniper: identification of somatic point mutations in whole genome sequencing data**. *Bioinformatics* 2012, **28**:311–317.

33. Radenbaugh AJ, Ma S, Ewing A, Stuart JM, Collisson EA, Zhu J, Haussler D: **RADIA: RNA and DNA integrated analysis for somatic mutation detection**. *PLoS One* 2014, **9**:e111516.

34. Lai Z, Markovets A, Ahdesmaki M, Chapman B, Hofmann O, McEwen R, Johnson J, Dougherty B, Barrett JC, Dry JR: **VarDict: a novel and versatile variant caller for next-generation sequencing in cancer research**. *Nucleic Acids Res* 2016, **44**:e108.

35. Rausch T, Zichner T, Schlattl A, Stütz AM, Benes V, Korbel JO: **DELLY: structural variant discovery by integrated paired-end and split-read analysis**. *Bioinformatics* 2012, **28**:i333–i339.

36. Layer RM, Chiang C, Quinlan AR, Hall IM: **LUMPY: A probabilistic framework for structural variant discovery**. *Genome Biol* 2014, **15**:R84.

37. Ye K, Schulz MH, Long Q, Apweiler R, Ning Z: **Pindel: a pattern growth approach to detect break points of large deletions and medium sized insertions from paired-end short reads**. *Bioinformatics* 2009, **25**:2865–2871.

38. Chen X, Schulz-Trieglaff O, Shaw R, Barnes B, Schlesinger F, Cox AJ, Kruglyak S, Saunders CT: **Manta: Rapid detection of structural variants and indels for clinical sequencing applications**. *bioRxiv* 2015:024232.

39. Favero F, Joshi T, Marquard AM, Birkbak NJ, Krzystanek M, Li Q, Szallasi Z, Eklund AC: **Sequenza: allele-specific copy number and mutation profiles from tumor sequencing data**. *Ann Oncol* 2015, **26**:64–70.

40. Ha G, Roth A, Khattra J, Ho J, Yap D, Prentice LM, Melnyk N, McPherson A, Bashashati A, Laks E, Biele J, Ding J, Le A, Rosner J, Shumansky K, Marra MA, Gilks CB, Huntsman DG, McAlpine JN, Aparicio S, Shah SP: **TITAN: inference of copy number architectures in clonal cell populations from tumor whole-genome sequence data**. *Genome Res* 2014, **24**:1881–1893.

41. McLaren W, Gil L, Hunt SE, Riat HS, Ritchie GRS, Thormann A, Flicek P, Cunningham F: **The Ensembl Variant Effect Predictor**. *Genome Biol* 2016, **17**:122.

42. Roth A, Khattra J, Yap D, Wan A, Laks E, Biele J, Ha G, Aparicio S, Bouchard-Côté A, Shah SP: **PyClone: statistical inference of clonal population structure in cancer**. *Nat Methods* 2014, **11**:396–398.

43. Andor N, Harness JV, Müller S, Mewes HW, Petritsch C: **EXPANDS: expanding ploidy and allele frequency on nested subpopulations**. *Bioinformatics* 2014, **30**:50–60.

44. Lawrence MS, Stojanov P, Polak P, Kryukov GV, Cibulskis K, Sivachenko A, Carter SL, Stewart C, Mermel CH, Roberts SA, Kiezun A, Hammerman PS, McKenna A, Drier Y, Zou L, Ramos AH, Pugh TJ, Stransky N, Helman E, Kim J, Sougnez C, Ambrogio L, Nickerson E, Shefler E, Cortés ML, Auclair D, Saksena G, Voet D, Noble M, DiCara D, et al.: **Mutational heterogeneity in cancer and the search for new cancer-associated genes**. *Nature* 2013, **499**:214–218.

45. Gonzalez-Perez A, Lopez-Bigas N: **Functional impact bias reveals cancer drivers**. *Nucleic Acids Res* 2012, **40**:e169.

46. Mermel CH, Schumacher SE, Hill B, Meyerson ML, Beroukhim R, Getz G: **GISTIC2.0 facilitates sensitive and confident localization of the targets of focal somatic copy-number alteration in human cancers**. *Genome Biol* 2011, **12**:R41.

47. P’ng C, Green J, Chong LC, Waggott D, Prokopec SD, Shamsi M, Nguyen F, Mak DYF, Lam F, Albuquerque MA, Wu Y, Jung EH, Starmans MHW, Chan-Seng-Yue MA, Yao CQ, Liang B, Lalonde E, Haider S, Simone NA, Sendorek D, Chu KC, Moon NC, Fox NS, Grzadkowski

MR, Harding NJ, Fung C, Murdoch AR, Houlahan KE, Wang J, Garcia DR, et al.: **BL.plotting.general: a package to visualize scientific data**. .

48. McLaren W, Pritchard B, Rios D, Chen Y, Flicek P, Cunningham F: **Deriving the consequences of genomic variants with the Ensembl API and SNP Effect Predictor**. *Bioinformatics* 2010, **26**:2069–2070.

Figure 1

Click here to download Figure fig1.png

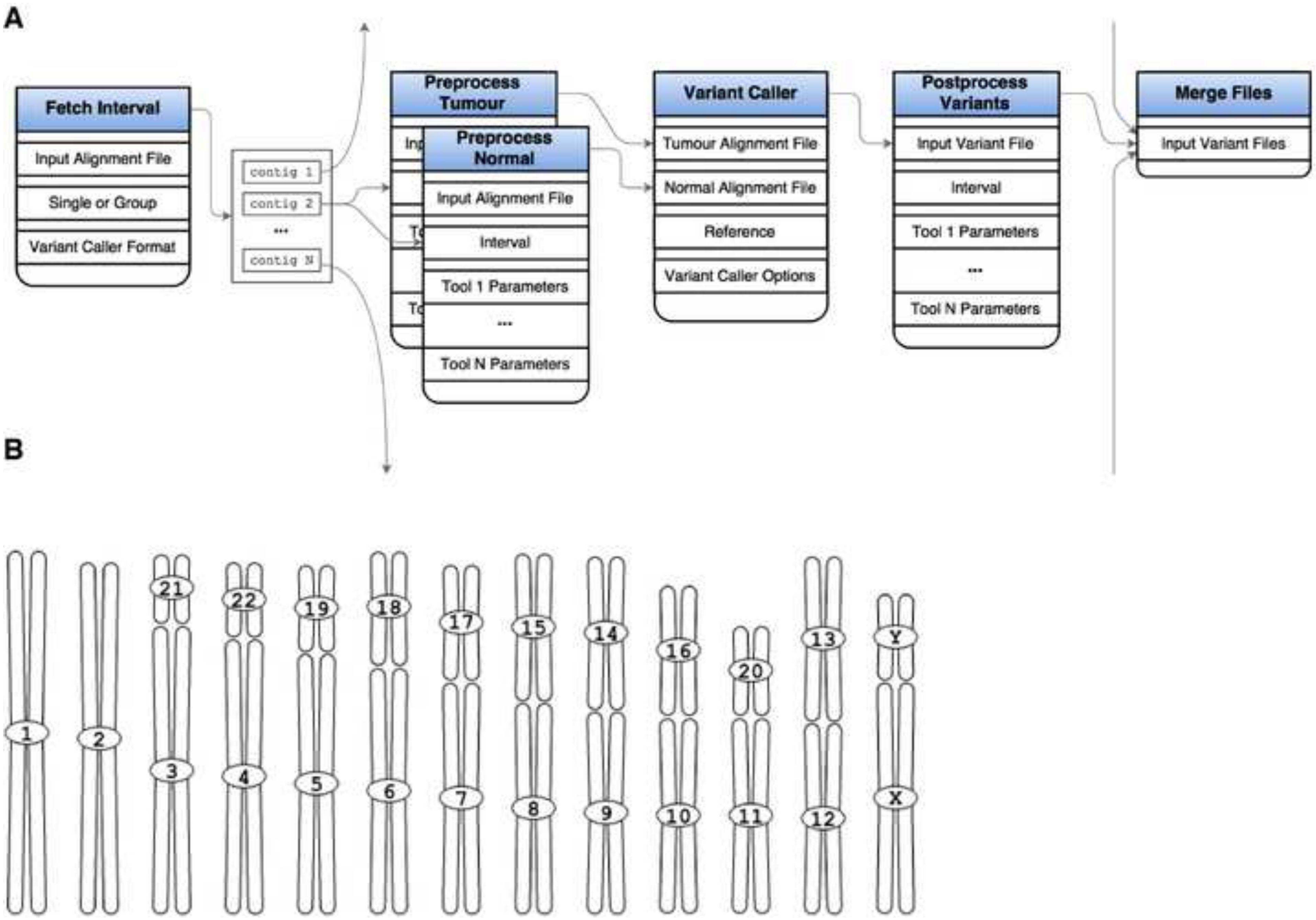

Figure 2

Click here to download Figure Fig2.png

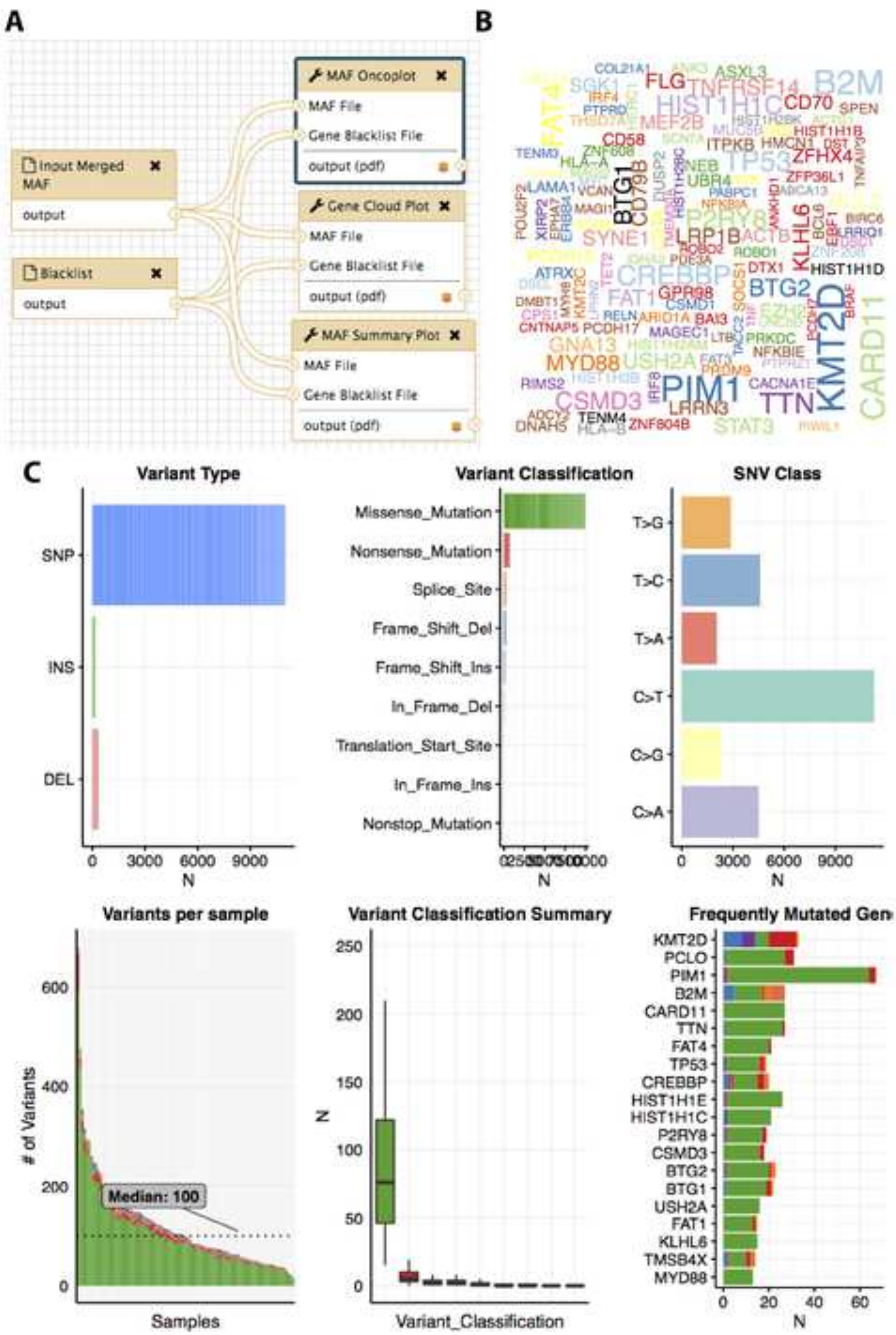

Figure 3

Click here to download Figure Fig3screenshot.png

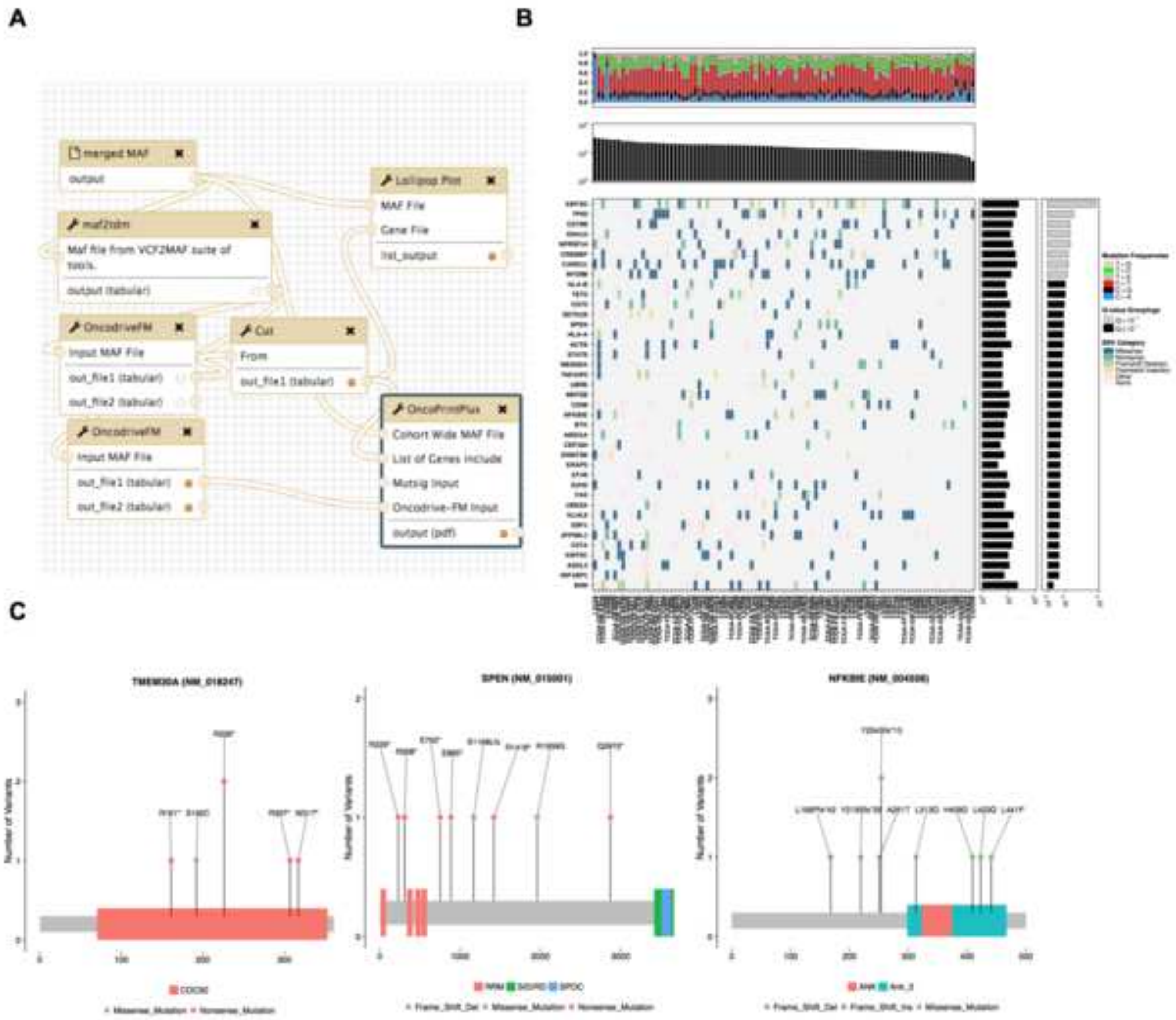

Figure 4

Click here to download Figure Fig4.png

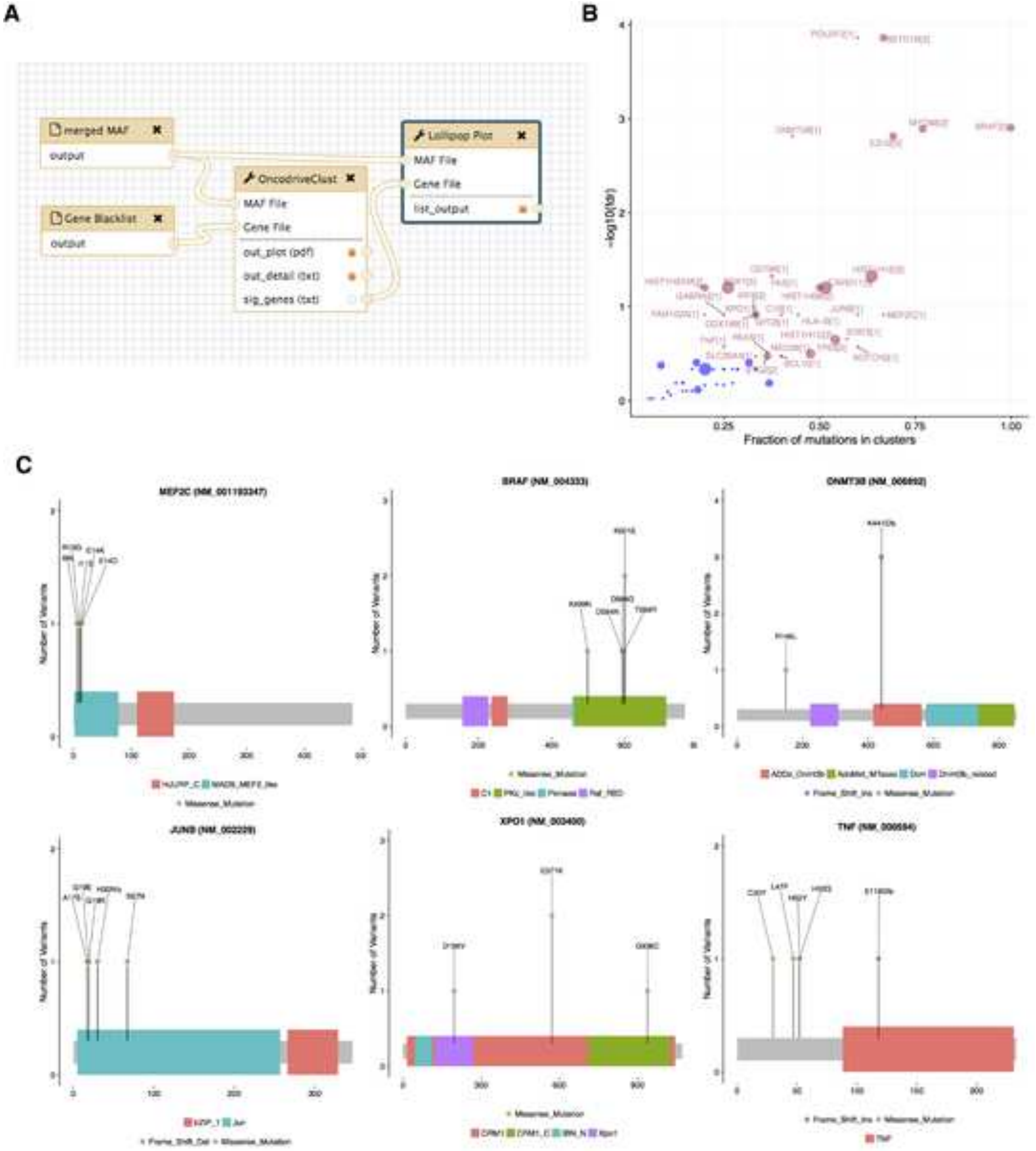

Figure 5

Click here to download Figure Fig5LegendNew.tif

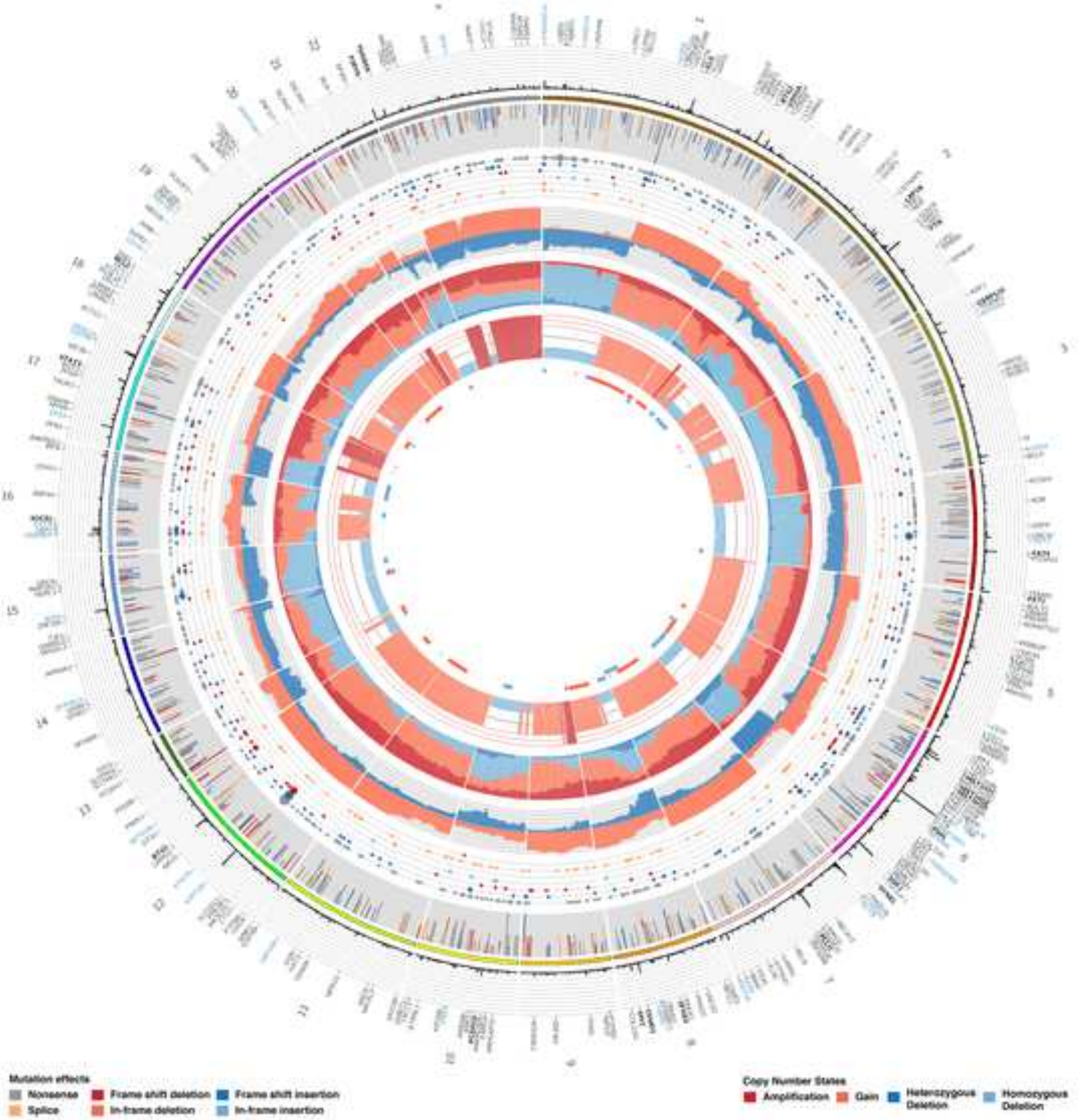

Figure 6

Click here to download Figure Fig6.png

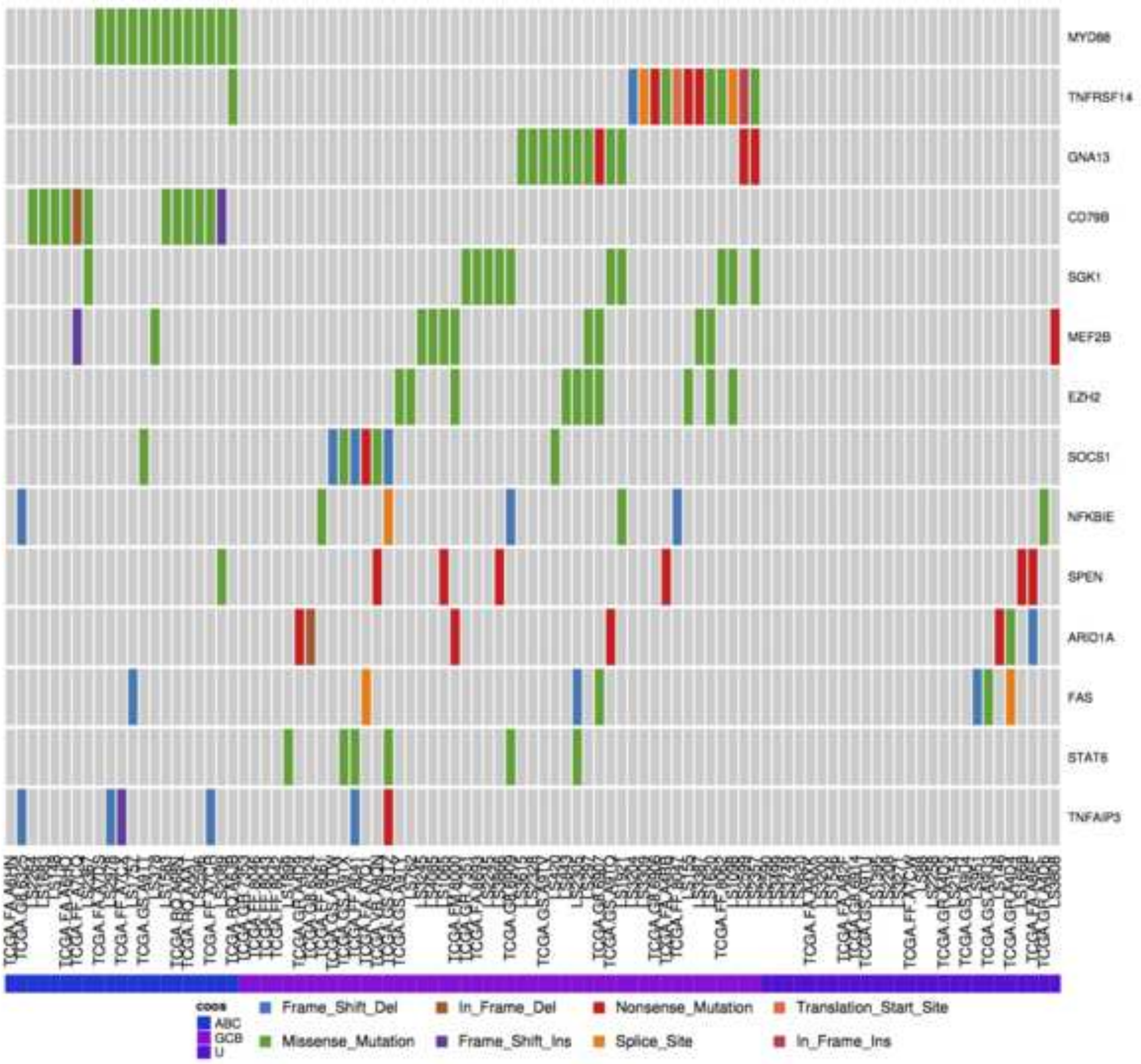

Click here to access/download
Supplementary Material
Additional Item 1 - Supplementary Tables Figures.pdf
