# Supplementary material for: Enhancing knowledge discovery from cancer genomics data with Galaxy
Source: Gigascience. 2017 Mar 9;6(5):1–13. doi: 10.1093/gigascience/gix015 (PMC5437943; doi:10.1093/gigascience/gix015)
Supplement: GIGA-D-16-00157_Revision_1.pdf [file gix015_GIGA-D-16-00157_Revision_1.pdf]

Manuscript
Click here to download Manuscript GalaxyDLBCL_for_gigascience_nofigs.pdf

Enhancing Knowledge Discovery from Cancer Genomics Data with Galaxy

Marco A. Albuquerque[1], Bruno M. Grande[1], Elie J. Ritch[1], Prasath Pararajalingam[1], Selin Jessa[1], Martin Krzywinski[2], Jasleen K. Grewal[1], Sohrab P. Shah[3], Paul C. Boutros[4] and Ryan D. Morin[1,2,*]

Author Affiliations

[1]Department of Molecular Biology and Biochemistry, Simon Fraser University, Burnaby, BC, Canada.

[2]Canada's Michael Smith Genome Sciences Center, BC Cancer Agency, Vancouver, BC, Canada.

[3]Department of Pathology, University of British Columbia, Vancouver, BC, Canada.

[4]Ontario Institute for Cancer Research, Toronto, ON, Canada.

Abstract

The field of cancer genomics has demonstrated the power of massively parallel sequencing techniques to inform on the genes and specific alterations that drive tumor onset and progression. Although large comprehensive sequence data sets continue to be made increasingly available, data analysis remains an ongoing challenge, particularly for laboratories lacking dedicated resources and bioinformatics expertise. To address this, we have produced a collection of Galaxy tools that represent many popular algorithms for detecting somatic genetic alterations from cancer genome and exome data. We developed new methods for parallelization of these tools within Galaxy to accelerate runtime and have demonstrated their usability and summarized their runtimes on multiple cloud service providers. Some tools represent extensions or refinement of existing toolkits to yield visualizations suited to cohort-wide cancer genomic analysis. For example, we present Oncocircos and Oncoprintplus, which generate data-rich

summaries of exome-derived somatic mutation. Workflows that integrate these to achieve data integration and visualizations are demonstrated on a cohort of 96 diffuse large B-cell lymphomas and enabled the discovery of multiple candidate lymphoma-related genes. Our toolkit is available from our GitHub repository as Galaxy tool and dependency definitions and has been deployed using virtualization on multiple platforms including Docker.

Keywords

Lymphoma; Driver; Cancer; Genome; Pipeline; Workflow; Tool; Cloud;

**To whom correspondence should be directed*

Dr. Ryan Morin; rdmorin@sfu.ca

Findings

Background

An inherent problem in the application of genomics to understand the molecular aetiology of cancer is the multi-disciplinary skillset required for researchers to draw meaningful inferences from high-throughput biological data. With the rise in popularity of high-throughput DNA sequencing, the bottleneck for novel discovery has shifted from data generation to data analysis and interpretation. Although myriad algorithms have been developed to efficiently analyze large datasets, these are often tailored for technically inclined users. Software for these analyses is typically run at the command line; operation requires the use of cryptic parameters; and installation is often burdensome. Achieving a flow of data between tools is also often non-trivial and, owing to a paucity of data standards, can involve error-prone data manipulation and re-formatting steps often relying on a collection of custom scripts that are often not released with publications. Combined with a necessity for high-performance computational hardware to run many such tools efficiently, these issues produce a tremendous barrier for new users.

There exist a handful of options that address this predicament in genomics as a whole. Tools that automate pipeline development such as Kronos [1], Nextflow [2] and Snakemake [3] can satisfy the needs of more technically savvy users. Alternatively, graphical user interfaces (GUIs)—which are generally lacking in the field of bioinformatics—aid in users learning the utility of the associated with command-line interfaces but typically do not scale to large data sets. Examples of genomics tools offering web-accessible GUIs include `BLAST` [4], `VAGUE` [5] and `limmaGUI` [6]. However, beyond an inability to scale, web-based utilities pose several issues, including design inconsistency, redundant efforts in interface development and the inability to automatically link individual tasks into pipelines or workflows. Ideally, any reduction in the barriers associated with running individual algorithms passing data between software tools should accelerate analytical tasks and reduce the risk of errors.

To overcome this, GUI-enabled software for automating pipeline development improves the reproducibility, accessibility and transparency of running genomic analyses [7]. Examples include Galaxy [8], Taverna [9], Pegasus [10] and commercial software packages such as Geneious [11]. In particular, the Galaxy project offers many attractive features for this goal while remaining open-source. Namely, Galaxy boasts extensive documentation; support for automatic tool installation; the ability to instantiate public or private “cloud clusters” by leveraging CloudMan [12]; and is as a whole supported by a vibrant community that provides ongoing development to the software and dedication to increasing the availability of bioinformatics software. Although algorithms for handling high-throughput sequence data are steadily being added to Galaxy, there currently remains a lack of tools and workflows tailored to perform common tasks involved in analyzing cancer genome and exome sequence data. Here, we have begun to address this issue by adapting many of the popular tools for analyzing cancer genome and exome data for Galaxy and made these publicly available as the Galaxy Cancer Genomics Toolkit.

Diffuse large B-cell lymphoma (DLBCL) is a common aggressive non-Hodgkin lymphoma that demonstrates extensive genetic heterogeneity with some genetic features found more common in only one of the two molecular subgroups, namely the ABC and GCB subgroups. Primary mediastinal B-cell lymphoma is defined as a separate entity by the World Health Organization with distinct clinical and diagnostic features but shares some genetic features with DLBCL and other lymphomas. Herein, we demonstrate the utility of the Galaxy Cancer Genomics Toolkit by applying the included workflows to a large cohort of DLBCL patients (n=96) and through a combination of analytical and exploratory approaches leveraging multiple visualization tools implemented within the Toolkit, we uncover new candidate lymphoma-related genes and putative genetic features associated with each molecular subgroup.

Implementing cancer genomics tools in Galaxy

We produced a comprehensive toolkit comprising a suite of complementary tools and workflows that perform many of the routine analytical tasks in cancer genomics. These include several popular methods for detecting ("calling") somatic single nucleotide variants (SNVs), copy number variations (CNVs) and structural variations (SVs) in tumour-normal pairs. We developed additional tools to perform the many auxiliary steps helper functions that allow tools to be linked and applied generically, such as bam and text file pre- and post-processing, manipulating and converting file formats; variant annotation; identification of significantly mutated genes; and visualizations for performing exploratory analysis and cohort-level data summarization. The tools and helper functions are briefly detailed in Table 1 and Table S1, respectively and documented in our repository.

To integrate individual tools into Galaxy, we implemented XML-based configuration files, which dictate the available inputs and arguments and build the command based on user-specified parameters. We adhered to a consistent design across tools of similar types.

Planemo, an integrated development environment for Galaxy tools, was used to assist with tool creation and ensure tool versions in a Github repository and Galaxy Toolshed were in sync [25]. All repositories are available on the public Galaxy test toolshed (https://testtoolshed.g2.bx.psu.edu/), which allows users to automatically install any tool [26]. Modular tool dependency repositories provide the step-by-step instructions for automatic download and installation of dependencies. Previously defined repositories were recycled if available. Though we could not successfully produce tools that automatically install on all platforms, many of our tools successfully install (with dependencies) on the standard Galaxy AWS image and in a custom Ubuntu installation (v16.04). We also note that the Galaxy community is migrating towards utilizing Conda for package management, which should ameliorate many of these issues moving forward. Synthetic alignment data containing artificial variants were generated and bundled with variant callers to enable automatic testing [27]. To handle reference data, we have developed tools to use Galaxy data tables and, for simplicity, allow the option of user-provided reference data [28].

Support within Galaxy for processing large data sets is still being established and one remaining restriction has been the lack of methods for splitting and parallelizing large analyses. We invested substantial effort to ensure that tools that perform analyses suitable for input-splitting are parallelizable on cluster environments wherever it was deemed desirable and possible. Following the addition of new data types in the Galaxy codebase, this was subsequently re-implemented using the more transparent and efficient method that exploits the more recent Galaxy feature known as “data collections”. Briefly, parallelization of a workflow is accomplished by a combination of tasks (Figure 1), beginning with `fetch_interval`. This obtains chromosome size information from each input read alignment file and creates a collection of BED files defining all complete chromosome intervals available to each tool. To balance the load across all concurrently spawned jobs, we automatically pair large and small

chromosomes if their sum is less than or equal to the length of the largest chromosome and we avoid splitting chromosomes into smaller intervals. This mimics the assumed longest lasting step for these algorithms while limiting the number of unnecessary concurrent jobs. The second, optional stage, is a `preprocess` tool which defines all necessary preprocessing steps in a single tool and all will be executed together to reduce the numerous outputs associated with running multiple separate preprocessing tools in Galaxy. This includes a `samtools` flag and mapping quality filter, `samtools` remove duplicates and `bamutils clipoverlap`. The third stage launches the selected tool on each of the intervals, allowing Galaxy to spawn processes to available CPUs. The fourth stage is `postprocess`, which follows similar methodology to `preprocess`. Example usage includes further variant filtration and annotation steps. Finally all output files are merged, if necessary, so they may be supplied to subsequent tools and workflows. For tools that can be multithreaded, we instead leverage this capability rather than chromosomal splitting and note that certain tools, for example structural variant callers, cannot be generally parallelized by input splitting.

The Galaxy toolkit has been thoroughly tested on multiple hardware configurations. Many of the analyses and workflows shown in detail here were performed on a local Galaxy instance on a Dell PowerEdge R430 Server with 2x Intel Xeon Processors (32 threads total) and 384 Gb of RAM running Ubuntu linux whereas benchmarking was performed separately on a Galaxy cluster on AWS Elastic Cloud Compute (EC2) (Methods). For computationally demanding tasks, we launched a Galaxy instance on AWS Elastic Cloud Compute (EC2) using CloudMan and installed the workflow and tool dependencies. A cluster consisting of one r3.8xlarge master node and five r3.2xlarge worker nodes was selected. From our experience, we advise using any nodes from the r3 family when running several jobs concurrently (cohort) as NFS issues may arise when using general-purpose nodes. If a few samples are to be run, general-purpose nodes are recommended. Separately, we produced a Dockerfile that will install our tools and

additional dependencies using the galaxy-stable Docker image (https://hub.docker.com/r/bgruening/galaxy-stable/~/dockerfile/). This is available in our GitHub repository, which also hosts the individual tools (https://github.com/morinlab/tools-morinlab). This Docker image was successfully built with automatic installation of tools and dependencies on our local Linux server and on the Google Cloud. We are working with this service to release an instance with reference genomes pre-installed that can be directly launched with minimal knowledge of Docker.

Selecting high-value tools and developing workflows for routine analytical tasks

There are numerous algorithms available to perform standard analytical tasks such as variant calling and CNV detection, each offering different balances of usability, computational efficiency and accuracy. As such, selection of ideal tools and parameters is non-trivial. We implemented tools representing some of the more commonly cited options and include many that performed favorably in ICGC-TCGA DREAM challenges [13]. As each tool can be configured with a number of parameters, which can be tuned for accuracy, we leverage results from the DREAM challenge to assist in selecting the more accurate algorithms and in setting sensible default parameters [14]. As ensemble approaches tend to provide increased robustness, we developed a tool to integrate variant calls from multiple algorithms using a simple voting scheme (Additional Items: Figure S1). We have also released numerous workflows that run some of the more complicated pieces of software that relies on many dependencies and that perform some routine analytical and visualization tasks as detailed and illustrated with the real-world worked examples below. Example workflows that demonstrate our new approach to perform parallelization in Galaxy are also included (Figure 1 and Additional items: Figure S2).

Benchmarking parallelized workflows running on Amazon Web Services

We uploaded 96 bam files representing the cohort of published DLBCL samples and ran all SNV and CNV workflows on this configuration for each tumour/normal pair and captured details on runtime and speedup associated with parallelization [21]. To assess the overhead potentially introduced when running tools in parallel, we ran the above cohort in both sequential and parallel modes across four different variant calling workflows. The runtime for each tool was collected from the local Galaxy database and summarised across the 48 pairs analyzed in the cloud. The average workflow runtime for an exome in parallel and sequential modes is shown in Table 2. The net change in estimated cost and speedup was averaged for each exome pair across using the time taken to complete all stages of the workflow (Figure 2). In general, the overhead associated with preprocessing adds marginally to the cost and yielded gains in speed as high as 8.6x. By comparing the cost gain and speedup in Figure 2, it is evident that some tools, for example Strelka, do not benefit from this mode of parallelization and instead should be run using the native parallelization on a node with more threads available. In the case of Strelka, there is a substantial pre-processing that occurs on each task to prepare the directory structure and configuration files, which is repeated several times using our parallelization method. Based on the current cost models, the approximate costs for running a single TN exome pair on all four workflows on AWS is $4.27 using sequential and $5.15 using parallelization, not including the cost of uploading the files or storing these or the reference files.

Identifying novel candidate lymphoma-related genes from exome data

An ultimate goal in cancer genome/exome analysis involves the identification of loci recurrently affected by copy number gain or loss and genes recurrently targeted by somatic mutations. There exist myriad tools to detect somatic SNVs and a growing number of options to derive high-quality copy number estimates from genome and exome data. We implemented

workflows that perform the required annotation and pre-processing of raw mutation and copy number outputs from tools such as `Strelka` and `Sequenza`, respectively. We ran these two workflows on 96 tumor/normal pairs representing diffuse large B-cell lymphoma (DLBCL) patients. The SNV and indel calls were annotated and converted to mutation annotation format (MAF) using `vcf2maf`. A variety of convenient visualization methods are available in the `maftools` R package [15] and Figure 3 shows the output of a workflow that employs some of these.

We next analyzed the pooled mutation calls from the meta-cohort for recurrently mutated genes using `OncodriveFM`. Figure 4 shows the workflow that performs these tasks and produces various visualizations of the resulting gene set. A batch tool built on `maftools` [15] was used to generate protein-centric lollipop plots, which can facilitate visual recognition of patterns indicative of tumor suppressor genes and can also reveal mutation clustering and hot spots (e.g. *TMEM30A* and *NFKBIE*). *TMEM30A* mutations have also been observed previously although their role remains unclear [16] and based on these visualizations we note a pattern towards protein inactivation and a hot spot in *NFKBIE* that induces a frameshift. The latter was recently observed in a separate set of patients with relapsed DLBCL [17]. *SPEN*, in contrast, has not been reported as recurrent target of somatic mutation in DLBCL but has been found mutated in other lymphoma types. The pattern of mutations suggests it may also act as a tumor suppressor gene in this cancer. We further noted *TET2*, *SETD1B*, *ARID1A*, *UBR5*, *DNMT3B* and *BTK* demonstrate similar mutation patterns (Figure 4B). Although these have been identified as relevant genes in other cancers [18,19], none of these have, to our knowledge, been previously ascribed to DLBCL.

We next attempted to integrate exome-derived copy number information with mutation calls. `Circos` is a popular approach to generate visualizations of genome-wide mutation data although it is generally better suited for genome-wide data and the representation of structural

alterations and CNVs relative to genomic coordinates [20]. We extended `Circos` to generate a gene-centric summarization of SNV and CNV data and produced the `Oncocircos` Galaxy tool. Rather than plotting on a genomic coordinate scale, gene-level summaries of point mutations are mapped to their relative order on each chromosome and intergenic space (and genes with mutations below the threshold) are eliminated. To accomplish this, we implemented a parser that tabulates the data from MAF and segmented copy number files, applying a threshold to restrict the display to genes with a greater number of mutations cohort-wide. `Oncocircos` also accepts user-provided gene lists and regions of recurrent CNV (e.g. from `GISTIC`) and highlights these in the resulting image (Additional Items: Figure S3). Figure 6 shows the result of a workflow that runs `GISTIC` on a merged set of segmented data (in this example, from the `Sequenza` workflow) and integrates annotated SNV and indel calls from `Strelka`. In this visualization, several known DLBCL-associated recurrent events are observed including amplifications affecting *REL*, *MYC* and *BCL2* respectively on 2p, 8q and 18q. Recurrent deletions affecting the loci containing known tumor suppressor genes are also observable. A complementary visualization of these data is a gene by patient `Oncostrip` in which annotated copy number and point mutations can be represented (Figure 7 and Additional Items: Figure S4).

Enabling new insights into DLBCL biology

The combined workflows employed here leverage distinct aspects of mutational information that can be individually leveraged to identify candidate cancer drivers and further integrated to inform on disease biology. Using a combination of methods, we provide additional evidence for the importance of several loci that have been attributed to DLBCL with weak support to date and those whose role as an oncogene or tumor suppressor has not been elucidated. By employing `OncodriveClust`, we identified genes with significant evidence for mutational recurrence.

Mutations around the V600E hot spot in *BRAF* and within *MEF2C* have previously been reported to be present, albeit rare, in DLBCL [21]. Another gene we found to harbor a hot spot was *STAT6* which, until recently, was thought to be mutated only in some less aggressive lymphomas such as FL and primary mediastinal B-cell lymphoma (PMBCL)[17]. A hot spot mutation in *XPO1* was also observed. This mutation has recently been suggested as a molecular marker of PMBCL distinguishes it from true DLBCLs [22]. One of the two cases bearing the canonical mutation (E571K) was among the few TCGA cases known to be PMBCLs and the other was from the second cohort for which clinical data was unavailable. These observations may further support the presence of mutations that will facilitate detection of PMBCL cases that can be difficult to distinguish from DLBCL by standard clinical criteria.

The integration of mutation with copy number data using our tools (Additional Items: Figure S4) has further informed on the potential relevance of some candidate lymphoma-related genes. *TMEM30A* demonstrated a mutation pattern indicative of tumor suppressor function (Figure 4C) and inspection of the `Oncocircos` image (Figure 6) suggests it resides within the commonly deleted region on 6q. Similarly, *FAT1* appears to have a strong signature towards inactivation and resides in a substantially smaller region that is commonly lost. Such patterns can be more readily confirmed using a separate visualization tool, namely `Oncostrip` (Additional Items: Figure S4). In contrast, some of the significantly amplified regions of the genome do not appear to harbor genes with significant evidence for recurrent mutations. Amplifications that include *JAK2* are known to be relevant to PMBCL but are not typically considered a feature of DLBCL. Upon inspection of the clinical data available for TCGA cases, we note that each of the four PMBCLs in this cohort contain a mutation or deletion affecting FAT1 and a JAK2 amplification. *POU2AF1*, which resides on 11q23.1, is a candidate target for the amplification of this region despite a low number of non-silent mutations and has been reported as commonly amplified in treatment-refractory DLBCLs [23]. Further studies that include larger cohorts and possibly whole

genome sequence data should help confirm the relevance of these observations.

Many of the genes known to be relevant to DLBCL biology are more commonly mutated in only one of the two molecular subgroups. Figure 7 shows the mutation distribution across some of these genes in the meta-cohort analyzed here, which has been organized on the predicted subgroup of each patient. Using the `Oncostrip` tool to order patients on this designation uncovers additional genes in which mutations may be more common in the GCB subgroup such as *NFKBIE*, *ARID1A*, *FAS* and *STAT6* (Additional Items: Figure S4). *NFKBIE* mutations have recently been reported to be particularly common among PMBCLs and a marker of poor prognosis in that disease [24]. One of the *NFKBIE* mutations detected herein was in a PMBCL case whereas the remainder were in nodal DLBCL cases and was almost exclusively seen in cases with other mutations suggestive of the GCB subgroup. This indicates a potential unappreciated role of *NFKBIE* in DLBCL or, taken together with our observation of mutations in *STAT6* and *XPO1*, may suggest that a significant subset of PMBCL cases may masquerade as GCB DLBCL. Further refinement of the mutation patterns of the two subgroups of DLBCL and PMBCL using larger cohorts is clearly warranted.

<u>Towards reproducible and distributable workflows for cancer genome analysis</u>

Large-scale efforts to understand the diversity of cancer-associated somatic alterations across common cancer types are continually expanding in scope. Many such efforts release raw (or aligned) tumor and normal sequence data into controlled-access repositories such as dbGAP and the European Genome-Phenome Archive (EGA). Owing to the many options and variations available in analytical methods, the mutations and copy number results presented along with such data are not directly amenable to direct comparisons between studies or pooled meta-analyses. Instead, the raw data must be obtained and processed uniformly alongside any new data sets. In light of the limited computational resources available to many research labs

interested in incorporating existing sequence data into their analyses, some consenting processes and major data repositories are beginning to facilitate storage and processing of patient data using cloud resources.

Our Galaxy Cancer Genomics Toolkit provides a growing list of standard methods for cancer genomic analysis and facilitates their deployment in a simplified, reproducible and accessible manner using Galaxy, which is amenable to deploying on standalone servers or on a variety of cloud services. We have run our tools and workflows using AWS cloud computing (with CloudMan), which provides a cluster environment to any research lab and on Google Cloud, which facilitates cluster management of Docker-based instances using Kubernetes. We continue to provide new tools by extending functionality and releasing updates. We also note that many of our tools have been tested on whole genome sequence data and additional tools for performing analytical tasks better suited to that data type have been implemented but were not described in detail here. To facilitate scaling of our applications to whole genome and exome data to the extent currently possible in this framework, and to accelerate the analysis of exomes, we established new methods to accomplish parallelization in Galaxy. It is important to note that Galaxy is not particularly well suited to certain large-scale analyses due to how data transfer tasks are handled, the internalization of some processes (e.g. bam indexing) and centralization of intermediate files generated by tools. We hope that ongoing development of the Galaxy codebase will improve on these and suggest that command-line equivalents to our workflows such as those offered by the Kronos software are worthy of consideration in larger-scale projects. Ongoing development of the Galaxy API may also ameliorate some of these issues.

This toolkit and its ready-made workflows provide the methods essential to drive discovery and eliminate the bottleneck in cancer genomic analysis and templates for creating similar analyses that leverage comparable software. Availability and usability of analytical software are

both critical factors in driving their adoption and the ultimate discovery of novel cancer drivers. Accordingly, we provide a series of solutions that should accelerate adoption of our toolkit. First, providing automatic installation for tools wherever possible allow seamless integration into custom Galaxy instances. Second, many of the tools and workflows included here can be optionally configured to efficiently parallelize tasks on a cluster environment. Third, we show that our toolkit can be readily deployed onto a cloud-based Galaxy instance thereby eliminating the need for permanent access to commodity computing hardware or dedicated systems administrators. Together, this offers the potential to enable reproducible cancer research by empowering researchers to perform their own cancer genome analyses with unprecedented accessibility and directly share their workflows such that other groups can reproduce these analyses on additional datasets.

As ownership of these tools migrates to the Intergalactic Utilities Commission along with transfer to the Main ToolShed, we encourage ongoing testing and parameter optimization and community-driven refinement and expansion of this toolkit. With sufficient adoption and ongoing support, this toolkit could empower numerous groups to explore the many available cancer data sets and their own experimental data using cloud infrastructure thereby facilitating the broader scientific community to make use of the steadily growing genomic resources being produced within this field.

Availability

All tools described herein are available in the Galaxy Test Toolshed and under the GPLv3 license via the project GitHub repository. The Dockerfile to automatically install these tools and a pre-built Docker image are also provided. The dependencies of each tool are documented in the associated tool dependency description and the Dockerfile and are too numerous to detail here. Instead, please refer to the source code: https://github.com/morinlab/tools-morinlab and

the tools-iuc repository.

Competing Interests

The authors declare they have no competing interests.

Authors' Contributions

M.A.A., S.J., P.P, and E.R. were responsible for deploying tools in galaxy. M.A.A. and B.M.G. tested workflows on AWS. M.A.A. and M.K. created figures. M.A.A., B.M.G. and R.D.M. wrote the manuscript, which was reviewed and approved by all authors. R.D.M, P.C.B. and S.P.S. led the study.

Acknowledgements

The results published here are in whole or part based upon data generated by the TCGA Research Network: http://cancergenome.nih.gov/. We gratefully acknowledge TCGA and all providers of samples and resources for generating this valuable resource. The TCGA exome data was obtained through dbGAP (phs000178.v9.p8 and phs000450.v2.p1) and the latter has been described previously [21]. Said data was produced as part of the Slim Initiative for Genomic Medicine (SIGMA), a joint U.S.-Mexico project funded by the Carlos Slim Health Institute. This work was supported by a contract from Genome Canada and Genome British Columbia (173CIC), funding from Mitacs (awarded to Morin) and Amazon AWS research grant. Sequencing of the large DLBCL cohort was funded by an operating grant from CIHR (to RDM). RDM is supported by New Investigator Awards from the Canadian Institutes for Health Research and the Terry Fox Research Institute. We thank all members of the Boutros, Shah and Morin research groups for feedback on this work. We also thank the Galaxy community for their ongoing support. We are particularly grateful to Enis Afgan, John Chilton, Nitesh Turaga

and Björn Gruening for their gracious assistance. We also thank Marija Jovanovic for assisting in tool deployment.

Table 1: Main tools currently comprising the cancer genomics toolkit.

Tool	Category	Reference
mutationSeq§	SNV detection	[29]
Strelka§	SNV and indel detection	[30]
SomaticSniper§	SNV detection	[31]
RADIA§	SNV detection	[32]
VarDict (Java)§	SNV detection	[33]
DELLY§	SV detection	[34]
LUMPY§	SV detection	[35]
Pindel§	SV and indel detection	[36]
Manta§	SV detection	[37]
Sequenza§	CNV detection	[38]
TITAN§	CNV detection	[39]
Ensembl VEP‡	SNV Annotation	[40]
PyClone§	Clonal structure	[41]
EXPANDS§	Clonal structure	[42]
MutSigCV§	Significantly Mutated Genes	[43]
Oncodrive-FM§	Significantly Mutated Genes	[44]
GISTIC§	Significantly Mutated Genes	[45]

Maftools (oncostrip, oncodriveclust, oncoplot, trinucleotide plot, genecloud, MAF summary, rainfall plot, lollipop plot)§	Visualization, significantly mutated genes, mutation signatures	[15]
Oncocircos∞	Visualization	[20]
Oncoprintplus∞	Visualization	[46]
igv_screenshot‡	Visualization	

Tools representing existing or extended analysis approaches are shown above. For a current list of tools available, refer to the repository.

∞New tool or visualization method created for this project. §New implementation of tool for existing software. ‡Existing Galaxy tool modified or extended for this project.

Table 2. Average cpu usage in hours when applying variant calling workflows to exome pairs.

Workflow	Sequential	Parallel
Preprocess (clipOverlap)	2.82	2.91
SNV : mutationSeq	5.68	5.99
SNV : Strelka	2.60	4.49
CNV : Titan	29.34	36.31
CNV : Sequenza	8.24	9.40

Figure 1. Parallelization in variant calling and other CPU-intensive processes.

An alignment file flows through to `fetch_interval`, which obtains all contigs in an alignment

file. If parallelization is requested, multiple interval files are generated for each interval, otherwise a single file is created. Each dataset in the collection is treated as separate input to two instance of `preprocess`, which filters reads from the sequence alignment file for normal and tumour alignment files. These then pass to the variant caller. A `postprocess` tool filters and annotates variant calls based on tool-specific parameters and all final variants are merged and sorted in a single variant file. We perform automatic interval selection to roughly balance the load on each variant-calling task. The algorithm combines regions (e.g. chromosomes) if their total length is less than the largest. In cloud-based settings, this reduces overhead associated with creating multiple unnecessary parallelized jobs as well as reducing the number of short-lived automatically added nodes. Importantly, we chose not to implement a sub-chromosomal interval selection algorithm to maintain intrachromosomal dependence required by some of the variant calling algorithms. Such an extension could be implemented for tools that lack this restriction.

Figure 2. Execution time and cost differences associated with parallelization.

(A) The average reduction in execution time was determined for all exome pairs analyzed on AWS by comparing the runtime with and without parallelization and is shown as the speedup. The preprocessing involved in setting up parallel tasks results in a reduced speedup and depends on the tool. (B) The actual CPU usage and dedicated machine type used by parallel implementations of the tool is shown as an estimate of the cost. Idle instances were not considered in this calculation. The difference in speedup and cost when preprocessing, the optional bam filtering step, is considered is reflected in the upper region of each bar.

Figure 3. Producing cohort-wide summaries and visualizations.

Following primary mutation detection across a large cohort and annotation (i.e. with VEP using

vcf2maf), it is useful to produce various summaries of the overall mutation burden and the types and classifications of mutations detected. The maftools R package offers a multitude of visualizations, many of which we have adapted into Galaxy. (A) In this example workflow, a merged MAF file containing the variants for the entire cohort of DLBCLs is input alongside a black-list of genes to hide from the outputs. (B) This word cloud, generated by the genecloud tool, provides a visually appealing summary of the frequency of mutations in genes above a user-specified threshold. (C) A generic mafsummaryplot tool provided by maftools generates six plots that represent descriptive features of the mutations and their annotations. It is evident that C>T is the predominant mutation type detected. A separate tool to perform refined mutation signature analysis is also available. Among the most commonly mutated genes are those previously attributed to DLBCL along with *TTN*, which encodes the largest human protein. With respect to the predicted effect, missense mutations are by far the dominant class of mutations. Despite this, tumor suppressors such as *KMT2D*, *TP53* and *B2M* show an elevation of inactivating mutation classes.

Figure 4. Significance analysis for mutation recurrence.

(A) Tools have been implemented to screen mutation data for patterns of recurrence and identify significantly mutated genes. Shown above is an example workflow that utilizes the OncodriveFM algorithm and generates various visualizations for genes meeting a pre-specified Q-value cutoff. (B) A common approach to summarize mutation data is a two-dimensional matrix with covariates plotted along the side axes. We implemented a tool that leverages multiplot in our R package to generate such images for arbitrary gene lists using the outputs of variant calling workflows that have been annotated using the vcf2maf tool. Mutations are colored based on the severity of mutations assigned automatically by the Ensembl Variant Effect Predictor (VEP) [47]. Genes with more severe mutations are more likely to be tumor

suppressor genes (e.g. *B2M* at the bottom and *TP53* and *KMT2D* at the top). Here, the total number of mutations detected in each patient is shown at the top and the P-value reported by `OncodriveFM` is shown for each gene is shown on the right. The frequency of each of six possible mutation type can inform on mutational processes in individual samples. This is automatically determined from MAF files and is summarized at the top. (C) It is also often desirable to visualize the pattern of mutations within individual genes. The pattern is revealed using the `lollipopplot` tool that is run on each gene passing the threshold in this workflow.

Figure 5. Identifying genes containing clustered mutations and hot spots.

With sufficiently large cohorts, the pattern of non-silent mutations within the protein can inform on genes under specific selective pressure. A clear pattern seen in many dominantly acting cancer genes are mutation hot spots. The `OncodriveClust` workflow searches for genes with significant clustering of mutations that may represent hot spots or regions/sites whose mutation may produce a dominant effect. Application of this workflow (A) detected many lymphoma-related genes known to harbor mutation clusters (B). The workflow automatically generates lollipop plots for all genes above a user-specified FDR (in this example, 0.3) (C). Clear patterns of hot spots or mutation clusters are visible in each of these genes with only *BRAF* and *MEF2C* having been previously attributed to some DLBCLs [21].

Figure 6. Visualization and data integration with `Oncocircos`.

The new `Oncocircos` tool allows visualization of segment data derived from the Titan and `Sequenza`-based workflows we implemented. Genes exceeding a user-specified mutation frequency across the cohort are displayed and labels are automatically added for top genes. Those with at least twice the minimum mutation threshold are labeled in bold and those in an optional user-specified list can also be colored. A black-list file can be optionally provided to hide genes known to be enriched for artefacts. Stacked bar plots and circles provide summary

of the annotated SNVs in each gene and a summary of the copy number state of each gene is provided in three inner tracks.

Figure 7. Discerning mutation patterns and identifying subtype-associated genes. DLBCL cases were assigned to either the ABC or GCB molecular subgroups using the presence of mutations known to be significantly restricted to either. Cases with no mutations unique to either molecular subgroup were designated unclassifiable (U).

References

1. Jafar Taghiyar, M. *et al.* Kronos: a workflow assembler for genome analytics and informatics. *bioRxiv* 040352 (2016). doi:10.1101/040352
2. Kurs, J. P., Simi, M. & Campagne, F. *Nextflow Workbench Documentation Booklet*. (Fabien Campagne, 2015).
3. Köster, J. & Rahmann, S. Snakemake--a scalable bioinformatics workflow engine. *Bioinformatics* **28,** 2520–2522 (2012).
4. Altschul, S. F. *et al.* Gapped BLAST and PSI-BLAST: a new generation of protein database search programs. *Nucleic Acids Res.* **25,** 3389–3402 (1997).
5. Powell, D. R. & Seemann, T. VAGUE: a graphical user interface for the Velvet assembler. *Bioinformatics* **29,** 264–265 (2013).
6. Wettenhall, J. M. & Smyth, G. K. limmaGUI: a graphical user interface for linear modeling of microarray data. *Bioinformatics* **20,** 3705–3706 (2004).
7. Goecks, J., Nekrutenko, A., Taylor, J. & Galaxy Team. Galaxy: a comprehensive approach for supporting accessible, reproducible, and transparent computational research in the life sciences. *Genome Biol.* **11,** R86 (2010).
8. Goecks, J., Nekrutenko, A., Taylor, J. & Galaxy Team. Galaxy: a comprehensive approach

for supporting accessible, reproducible, and transparent computational research in the life sciences. *Genome Biol.* **11,** R86 (2010).

9. Zhang, H., Hyde, Z., Stian, S.-R. & Carole, G. Taverna Mobile: Taverna workflows on Android. *EMBnet.journal* **19,** 43 (2013).

10. Deelman, E. *et al.* Pegasus, a workflow management system for science automation. *Future Gener. Comput. Syst.* **46,** 17–35 (2015).

11. Kearse, M. *et al.* Geneious Basic: an integrated and extendable desktop software platform for the organization and analysis of sequence data. *Bioinformatics* **28,** 1647–1649 (2012).

12. Afgan, E. *et al.* Galaxy CloudMan: delivering cloud compute clusters. *BMC Bioinformatics* **11 Suppl 12,** S4 (2010).

13. Boutros, P. C. *et al.* Global optimization of somatic variant identification in cancer genomes with a global community challenge. *Nat. Genet.* **46,** 318–319 (2014).

14. Ewing, A. D. *et al.* Combining tumor genome simulation with crowdsourcing to benchmark somatic single-nucleotide-variant detection. *Nat. Methods* **12,** 623–630 (2015).

15. Mayakonda, A. & Phillip Koeffler, H. Maftools: Efficient analysis, visualization and summarization of MAF files from large-scale cohort based cancer studies. *bioRxiv* 052662 (2016). doi:10.1101/052662

16. Morin, R. D. *et al.* Frequent mutation of histone-modifying genes in non-Hodgkin lymphoma. *Nature* **476,** 298–303 (2011).

17. Morin, R. D. *et al.* Genetic Landscapes of Relapsed and Refractory Diffuse Large B-Cell Lymphomas. *Clin. Cancer Res.* **22,** 2290–2300 (2016).

18. Meissner, B. *et al.* The E3 ubiquitin ligase UBR5 is recurrently mutated in mantle cell lymphoma. *Blood* **121,** 3161–3164 (2013).

19. Wiegand, K. C. *et al.* ARID1A mutations in endometriosis-associated ovarian carcinomas. *N. Engl. J. Med.* **363,** 1532–1543 (2010).

20. Krzywinski, M. *et al.* Circos: an information aesthetic for comparative genomics. *Genome Res.* **19,** 1639–1645 (2009).

21. Lohr, J. G. *et al.* Discovery and prioritization of somatic mutations in diffuse large B-cell lymphoma (DLBCL) by whole-exome sequencing. *Proc. Natl. Acad. Sci. U. S. A.* **109,** 3879–3884 (2012).

22. Jardin, F., Pujals, A., Pelletier, I. & Others. Whole exome sequencing of refractory aggressive B-cell lymphomas identified recurrent mutations of the exportin 1 gene (XPO1) in primary mediastinal B-cell lymphoma subtype, a LYSA study. *Hematol. Oncol.* **33,** 100–180 (2015).

23. Park, H. Y. *et al.* Whole-exome and transcriptome sequencing of refractory diffuse large B-cell lymphoma. *Oncotarget* (2016). doi:10.18632/oncotarget.13239

24. Mansouri, L. *et al.* Frequent NFKBIE deletions are associated with poor outcome in primary mediastinal B-cell lymphoma. *Blood* (2016). doi:10.1182/blood-2016-03-704528

25. galaxyproject. galaxyproject/planemo. *GitHub* Available at: https://github.com/galaxyproject/planemo. (Accessed: 23rd November 2015)

26. Blankenberg, D. *et al.* Dissemination of scientific software with Galaxy ToolShed. *Genome Biol.* **15,** 403 (2014).

27. Ewing, A. D. *et al.* Combining tumor genome simulation with crowdsourcing to benchmark somatic single-nucleotide-variant detection. *Nat. Methods* **12,** 623–630 (2015).

28. Blankenberg, D., Johnson, J. E., Galaxy Team, Taylor, J. & Nekrutenko, A. Wrangling Galaxy's reference data. *Bioinformatics* **30,** 1917–1919 (2014).

29. Ding, J. *et al.* Feature-based classifiers for somatic mutation detection in tumour-normal paired sequencing data. *Bioinformatics* **28,** 167–175 (2012).

30. Saunders, C. T. *et al.* Strelka: accurate somatic small-variant calling from sequenced tumor-normal sample pairs. *Bioinformatics* **28,** 1811–1817 (2012).

31. Larson, D. E. *et al.* SomaticSniper: identification of somatic point mutations in whole genome sequencing data. *Bioinformatics* **28,** 311–317 (2012).

32. Radenbaugh, A. J. *et al.* RADIA: RNA and DNA integrated analysis for somatic mutation detection. *PLoS One* **9,** e111516 (2014).

33. Lai, Z. *et al.* VarDict: a novel and versatile variant caller for next-generation sequencing in cancer research. *Nucleic Acids Res.* **44,** e108 (2016).

34. Rausch, T. *et al.* DELLY: structural variant discovery by integrated paired-end and split-read analysis. *Bioinformatics* **28,** i333–i339 (2012).

35. Layer, R. M., Chiang, C., Quinlan, A. R. & Hall, I. M. LUMPY: A probabilistic framework for structural variant discovery. *Genome Biol.* **15,** R84 (2014).

36. Ye, K., Schulz, M. H., Long, Q., Apweiler, R. & Ning, Z. Pindel: a pattern growth approach to detect break points of large deletions and medium sized insertions from paired-end short reads. *Bioinformatics* **25,** 2865–2871 (2009).

37. Chen, X. *et al.* Manta: Rapid detection of structural variants and indels for clinical sequencing applications. *bioRxiv* 024232 (2015). doi:10.1101/024232

38. Favero, F. *et al.* Sequenza: allele-specific copy number and mutation profiles from tumor sequencing data. *Ann. Oncol.* **26,** 64–70 (2015).

39. Ha, G. *et al.* TITAN: inference of copy number architectures in clonal cell populations from tumor whole-genome sequence data. *Genome Res.* **24,** 1881–1893 (2014).

40. McLaren, W. *et al.* The Ensembl Variant Effect Predictor. *Genome Biol.* **17,** 122 (2016).

41. Roth, A. *et al.* PyClone: statistical inference of clonal population structure in cancer. *Nat. Methods* **11,** 396–398 (2014).

42. Andor, N., Harness, J. V., Müller, S., Mewes, H. W. & Petritsch, C. EXPANDS: expanding ploidy and allele frequency on nested subpopulations. *Bioinformatics* **30,** 50–60 (2014).

43. Lawrence, M. S. *et al.* Mutational heterogeneity in cancer and the search for new cancer-

associated genes. *Nature* **499,** 214–218 (2013).

44. Gonzalez-Perez, A. & Lopez-Bigas, N. Functional impact bias reveals cancer drivers. *Nucleic Acids Res.* **40,** e169 (2012).

45. Mermel, C. H. *et al.* GISTIC2.0 facilitates sensitive and confident localization of the targets of focal somatic copy-number alteration in human cancers. *Genome Biol.* **12,** R41 (2011).

46. P’ng, C. *et al.* BL.plotting.general: a package to visualize scientific data.

47. McLaren, W. *et al.* Deriving the consequences of genomic variants with the Ensembl API and SNP Effect Predictor. *Bioinformatics* **26,** 2069–2070 (2010).

Figure 1 Click here to download Figure Fig1.png

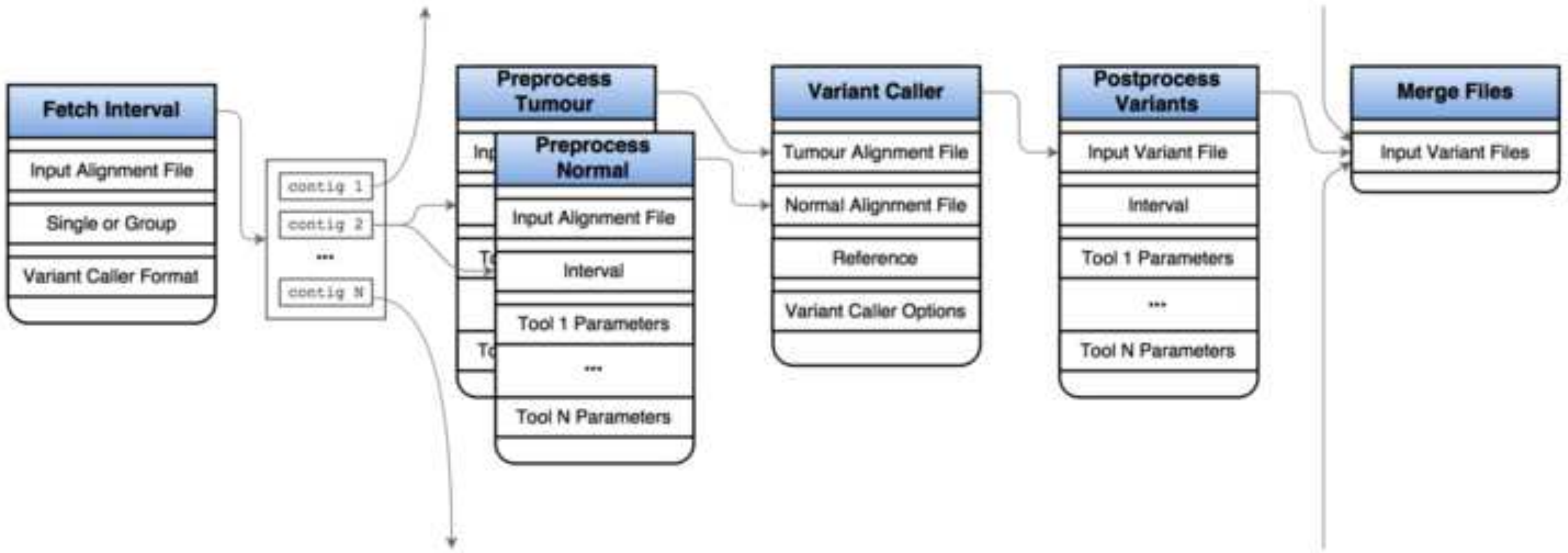

Figure 2

Click here to download Figure Figure_2.png

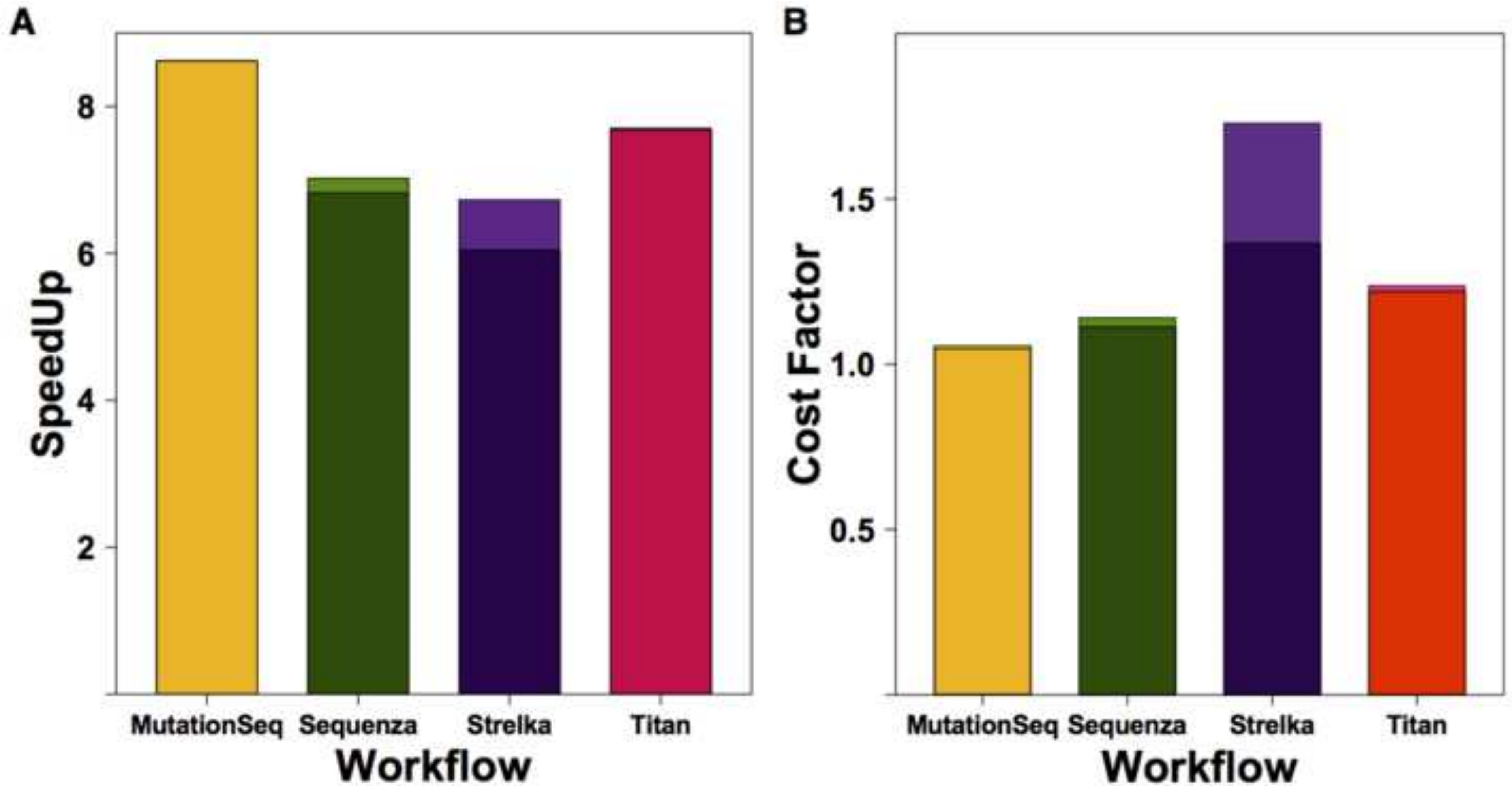

Figure 3 Click here to download Figure Figure3.png

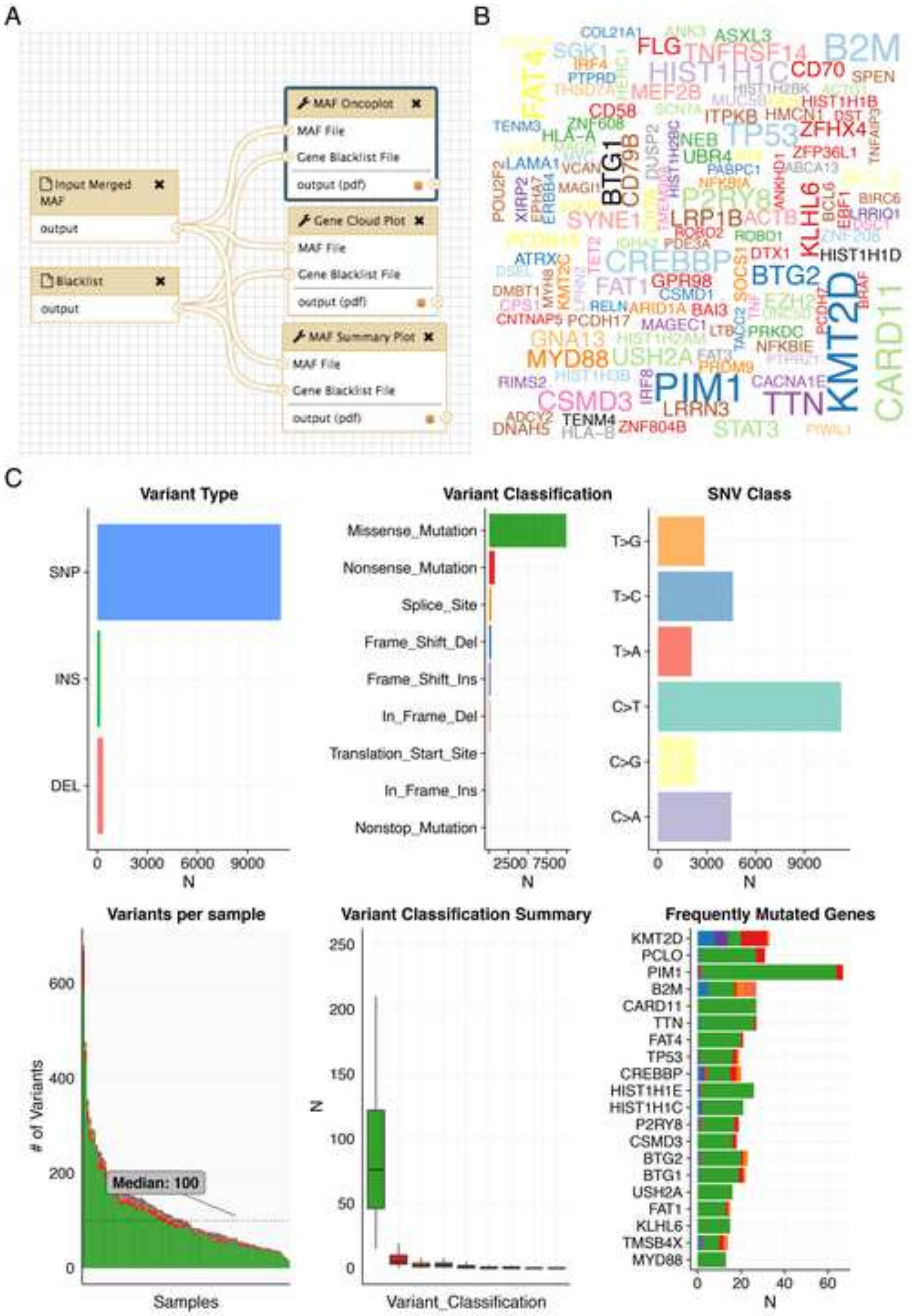

Figure 4

Click here to download Figure Figure4.png

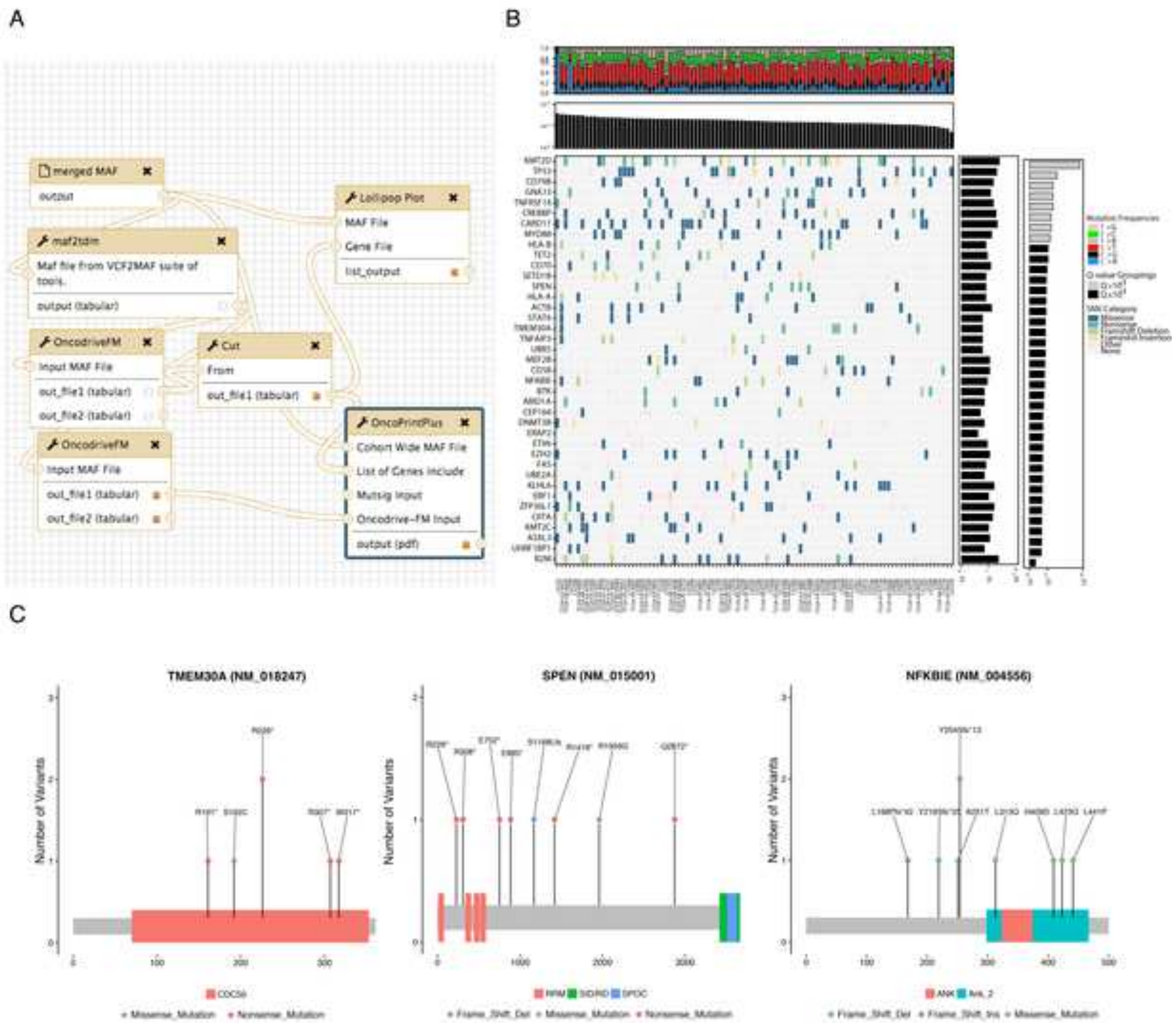

Figure 5 Click here to download Figure Figure5.png

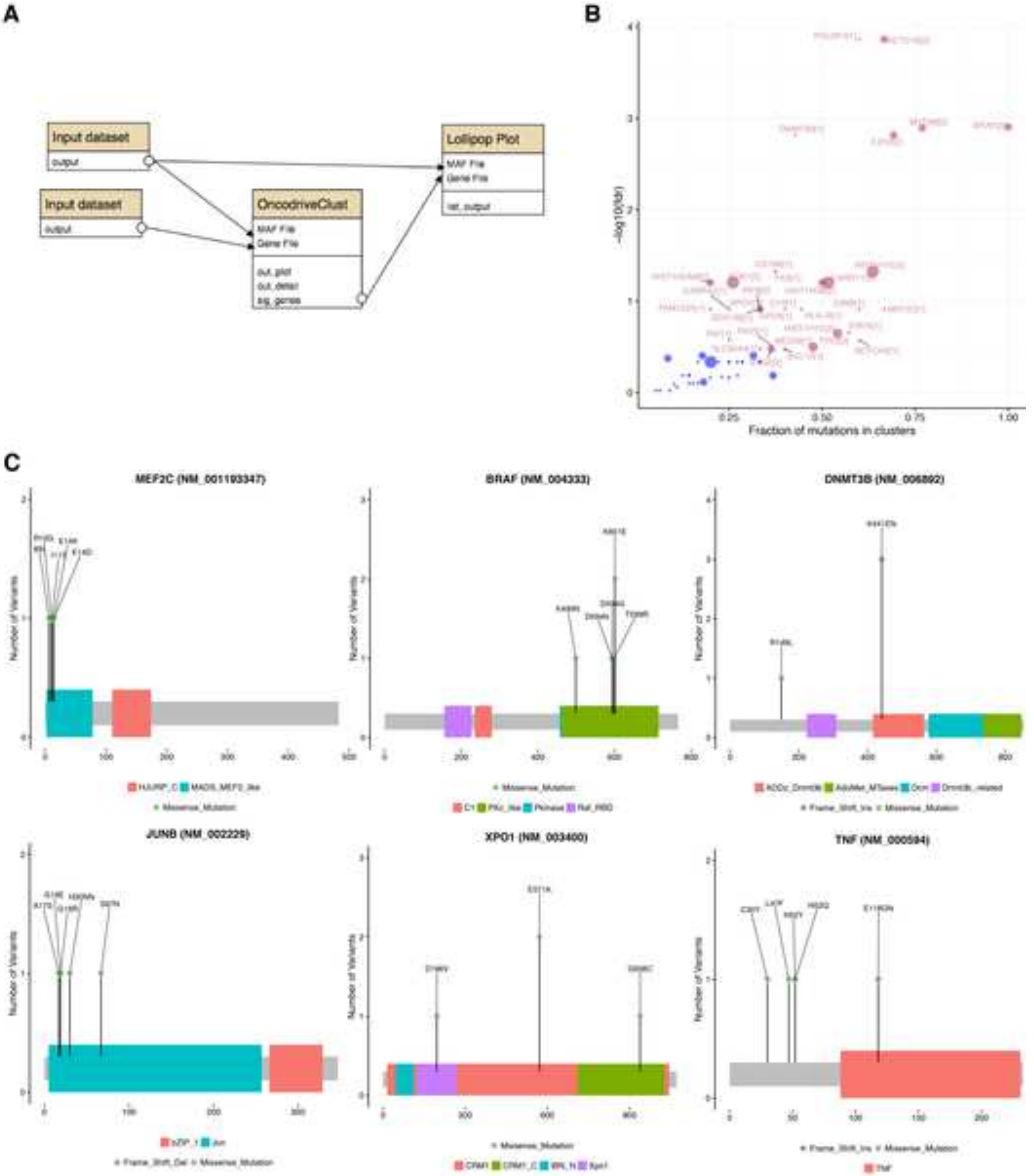

Figure 6

Click here to download Figure Fig6.png

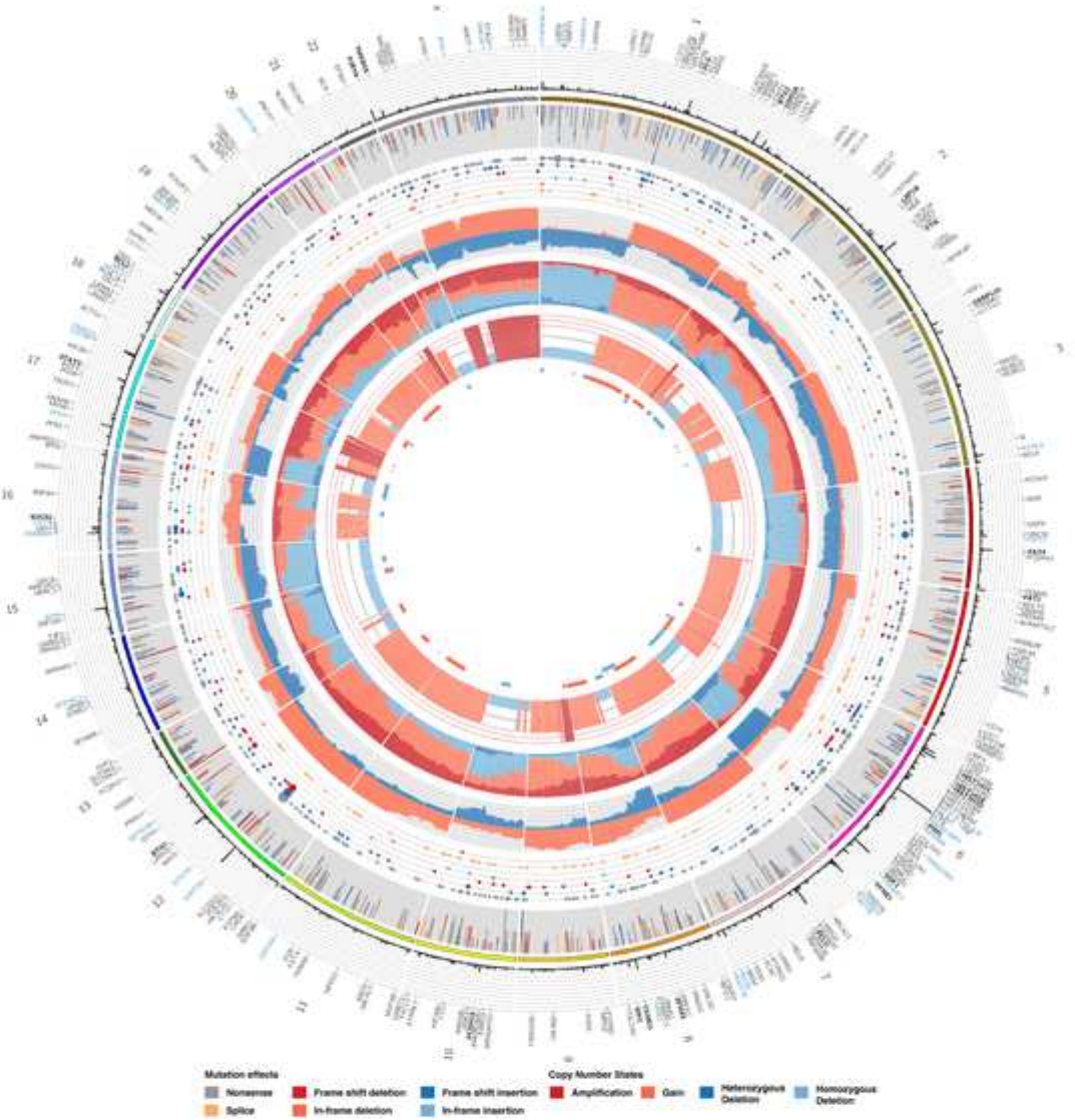

Figure 7

Click here to download Figure Fig7.png

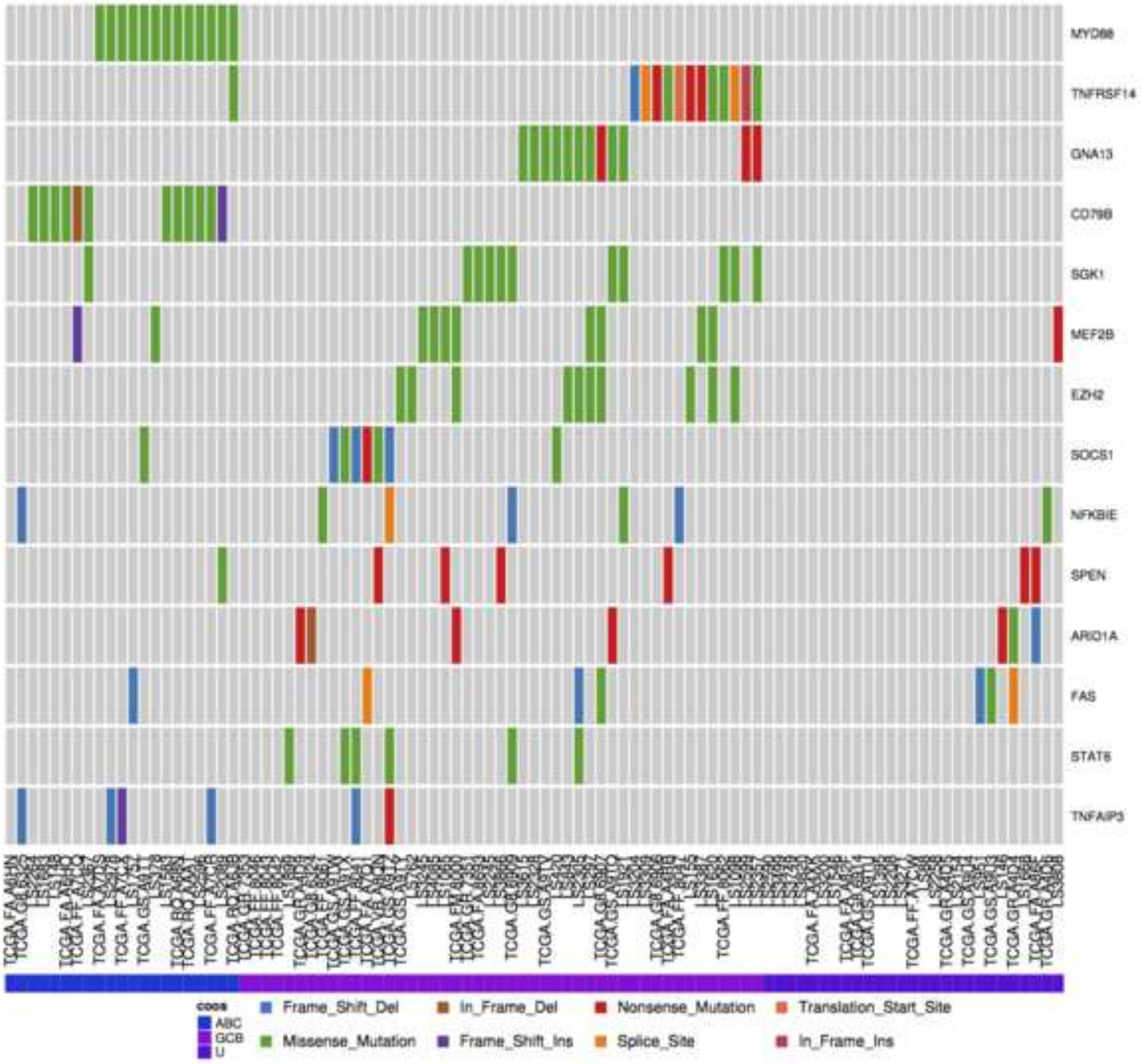

Click here to access/download
Supplementary Material
Additional Item 1 - Supplementary Tables Figures.pdf

Personal Cover
Click here to download Personal Cover
Gigasciencecoverletter.pdf

Scott Edmunds, PhD
Gigascience

Dear Dr. Edmunds.

. Please find attached our substantially revised and improved manuscript entitled **“Enhancing Knowledge Discovery from Cancer Genomics Data using Galaxy**”. I thank you and Nicole for sending it out for rapid review and hope you will agree that each reviewer found our work important and interesting and worthy of publication in Gigascience. As you will see in the attached response to reviewers document, we have addressed all the concerns of the two reviewers. In parallel, we have made numerous other improvements to the code and have performed more testing and benchmarking in additional cloud providers such as Google. As you probably noted in Melbourne, the Galaxy community is engaging with this project and I am working with Bjorn Gruening and others to bring our tools into the main tool shed to enhance their adoption.

I look forward to your decision on this submission.

Best,

Ryan D. Morin

Department of Molecular Biology and Biochemistry,

Simon Fraser University,

BC, Canada
