# Supplementary material for: Enhancing knowledge discovery from cancer genomics data with Galaxy
Source: Gigascience. 2017 Mar 9;6(5):1–13. doi: 10.1093/gigascience/gix015 (PMC5437943; doi:10.1093/gigascience/gix015)
Supplement: Supplemental material — Additional file Fig. S1 An ensemble approach to detect somatic SNVs. The ensembl_vcf tool receives the output of variant callers and selects variants detected by a user-specified number of tools. This example workflow runs four variant callers (strelka, mutationSeq, RADIA and SomaticSniper) and runs vcf2maf to annotate the resulting list of variants with support from a sufficient number of tools. Additional file Fig. S2 Achieving parallelization in Galaxy. There are two ways to achieve parallelization in Galaxy. The first (A) employs the parallelism tag, which calls specific split and merge functions depending on the Galaxy input and output data type. These functions are predefined within galaxies codebase. The second (B) uses galaxy collections, which are essentially containers of input files. Inputs can be split into a collection of files and subsequently pipeline these through a series of tools. When complete, the individual outputs can be merged. Parallelizing using collections is far more transparent to the user and also limits that number of unnecessary split and merge functions. Additional file Fig. S3 A workflow to integrate SNV and CNV data and produce integrative visualizations. This workflow uses exome-derived CNV and SNV data to generate a list of recurrently gained/lost genomic regions (using GISTIC) and displays these along with gene-centric summaries of segmented copy number and SNV data using Oncocircos. To generate Fig. 5, we included a blacklist containing all immunoglobulin genes and Mucin genes whereas the genes identified as significantly mutated by oncodriveFM were provided separately to enforce highlighting. Additional file Fig. S4 Using the Oncostrip tool to integrate copy number and mutation data. It can be desirable to visualize the complete set of mutational information cohort-wide without losing the patient-mutation relationships and potential gene-gene interactions that are not retained in Oncocircos. For this application, the Oncostrip [file gix015_Supp.pdf]

Additional Item 1:

Supplementary Tables and Figure Legends for Albuquerque *et al*, 2016

Table S1. Helper tools implemented to facilitate tool linkage and parallelization.

Tool name	Description	Application
ensemble_vcf	An ensemble variant caller that applies a voting scheme	Select high-confidence SNV calls from the outputs of multiple variant callers
augment_maf	Add allele counts for each variant from tumour and normal bam files and integrate mutations from multiple samples.	A key step in the process in preparing input for PyClone
ensembl_vep	Annotate effect of SNVs and indels using the Ensembl build	Used by vcf2maf to produce annotated MAF files from raw mutation calls in VCF format
fetch_interval	Reads a Binary Sequence Alignment File (BAM) and writes out intervals in tab-delimited format	Preprocessing in parallelization
merge, merge_gzip	Merge while maintaining order of text files in a data collection	Postprocessing in parallelization
merge_maf_collection	Merge a collection of MAF files and handle headers	Creating cohort-wide MAF file from individual samples
preprocess	Split and process inputs	Required to allow parallelization
cnv2igv	Reformat outputs from CNV callers into standard IGV-friendly input	Produce inputs for GISTIC and Oncocircos tools
igv2gistic	Incorporate exon annotations to produce segmented data and marker file	Preprocessing necessary to run GISTIC2.0 on exome-derived CNV data
select_optimal_cluster	Nominate Titan run from a sample with the best fit	Postprocess Titan output to yield best result for further analysis

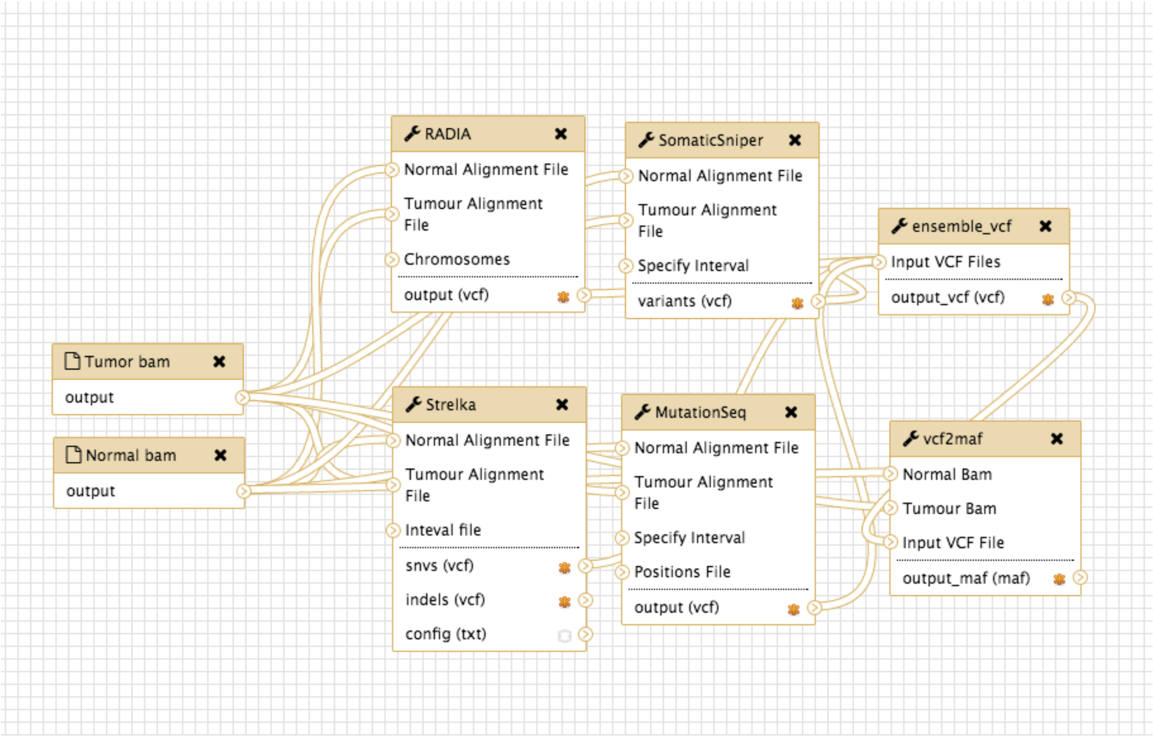

Figure S1. An ensemble approach to detect somatic SNVs.

The `ensembl_vcf` tool receives the output of variant callers and selects variants detected by a user-specified number of tools. This example workflow runs four variant callers (`strelka`, `mutationSeq`, `RADIA` and `SomaticSniper`) and runs `vcf2maf` to annotate the resulting list of variants with support from a sufficient number of tools.

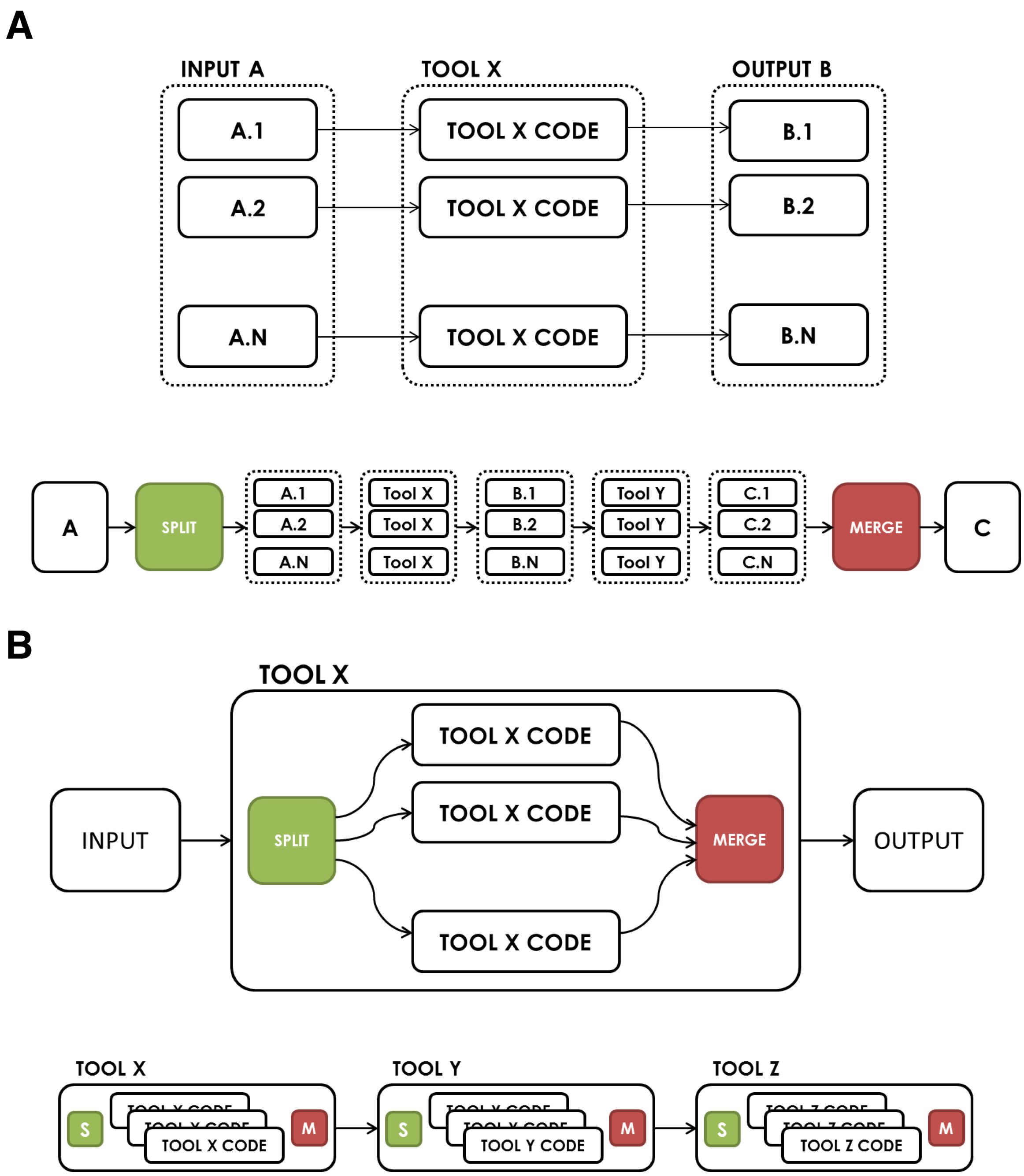

Figure S2. Achieving parallelization in Galaxy.

There are two ways to achieve parallelization in Galaxy. The first (A) employs the parallelism tag, which calls specific split and merge functions depending on the galaxy input and output data type. These functions are predefined within galaxies codebase. The second (B) uses galaxy collections, which are essentially containers of input files.

Inputs can be split into a collection of files and subsequently pipeline these through a series of tools. When complete, the individual outputs can be merged. Parallelizing using collections is far more transparent to the user and also limits that number of unnecessary split and merge functions.

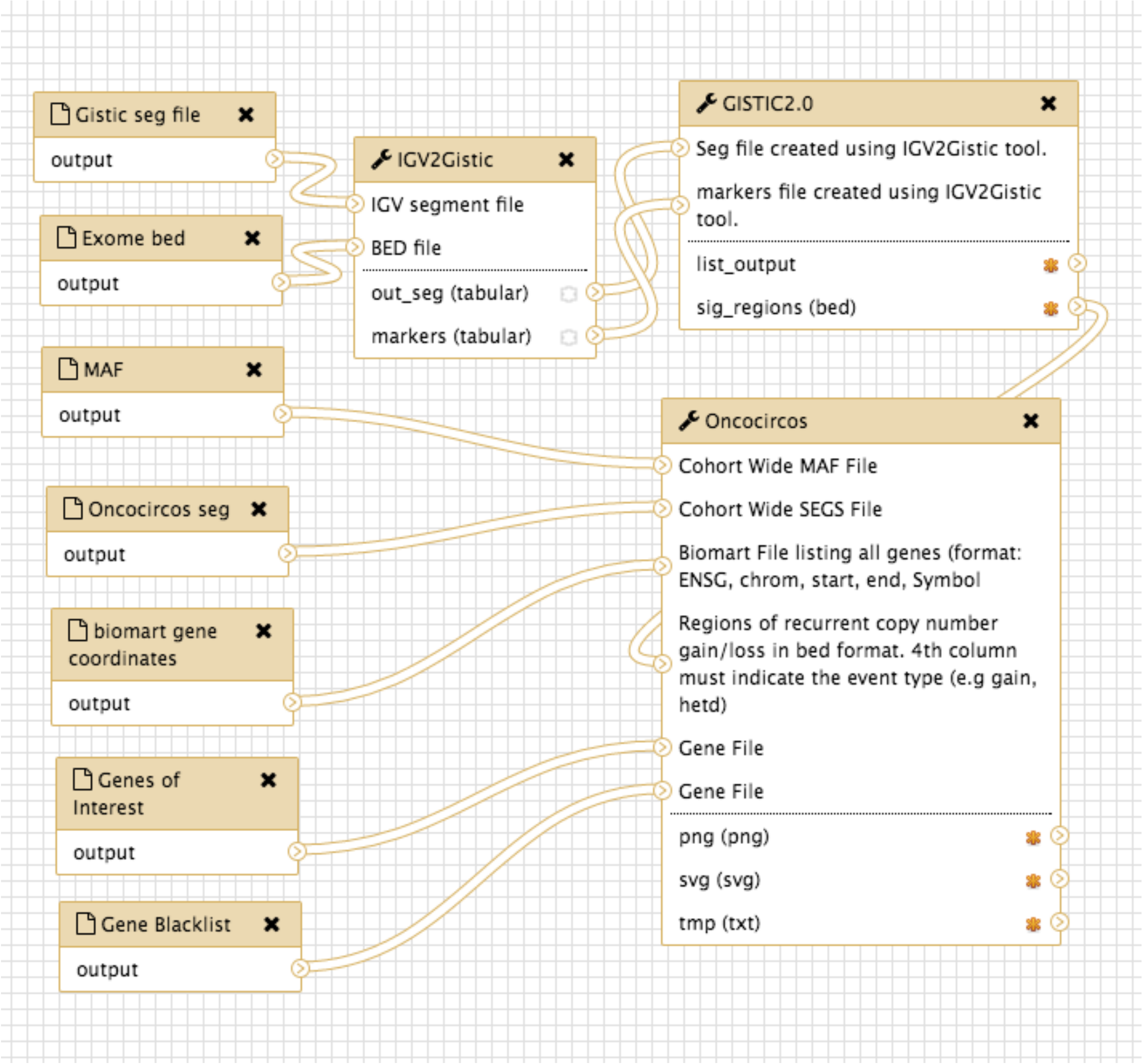

Figure S3. A workflow to integrate SNV and CNV data and produce integrative visualizations.

This workflow uses exome-derived CNV and SNV data to generate a list of recurrently gained/lost genomic regions (using `GISTIC`) and displays these along with gene-centric summaries of segmented copy number and SNV data using `Oncocircos`. To generate

Figure 5, we included a blacklist containing all immunoglobulin genes and Mucin genes whereas the genes identified as significantly mutated by `oncodriveFM` were provided separately to enforce highlighting.

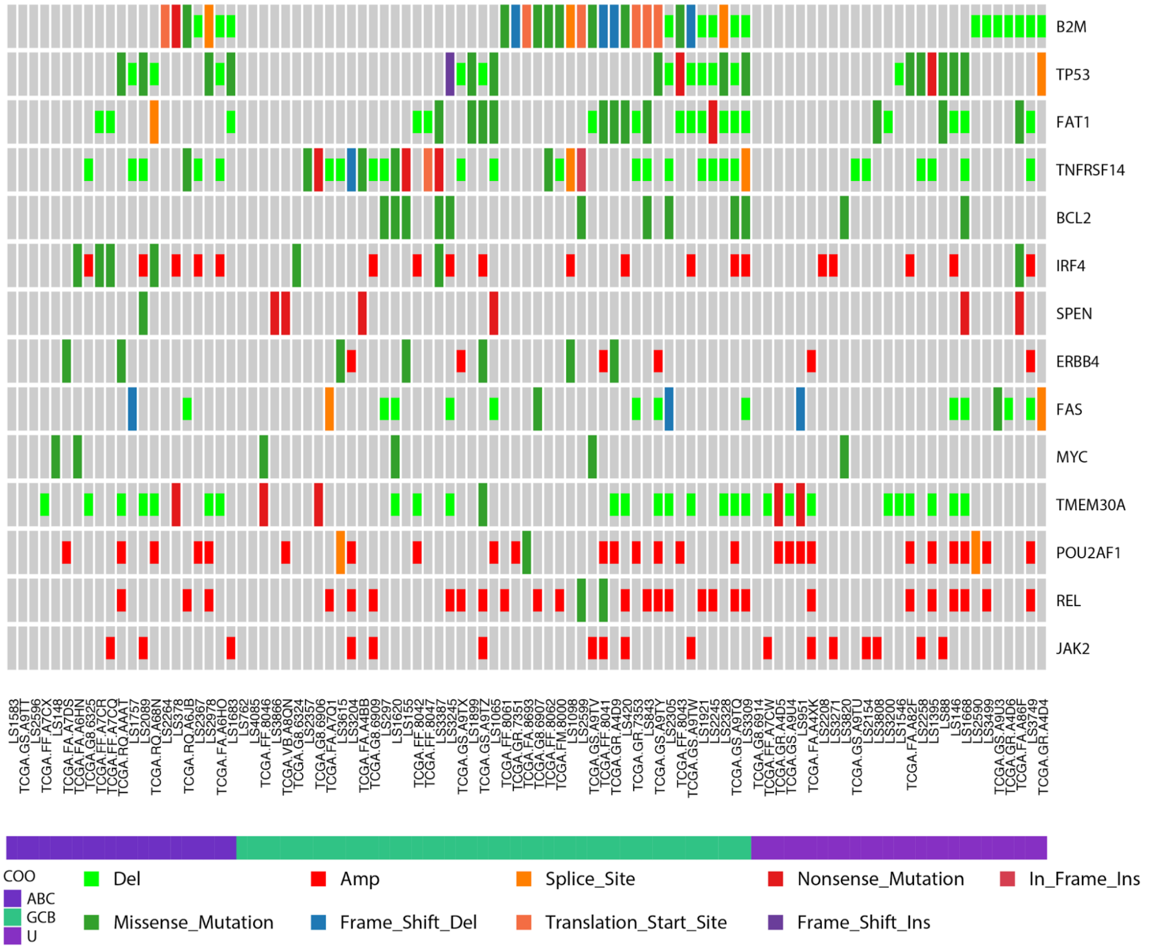

Figure S4. Using the `Oncostrip` tool to integrate copy number and mutation data.

It can be desirable to visualize the complete set of mutational information cohort-wide without losing the patient-mutation relationships and potential gene-gene interactions that are not retained in `Oncocircos`. For this application, the `Oncostrip` component of `maftools` can also accept raw outputs from `GISTIC`. Here, we have included the known gene targets of some recurrent amplifications and deletions detected in the cohort (*REL*, B2M and *TNFRSF14*). Each of *FAT1* and *TMEM30A* reside in significantly

deleted regions and bear a combined pattern of mutation and deletion consistent with tumor suppressor function.
