# Supplementary material for: Enhancing knowledge discovery from cancer genomics data with Galaxy
Source: Gigascience. 2017 Mar 9;6(5):1–13. doi: 10.1093/gigascience/gix015 (PMC5437943; doi:10.1093/gigascience/gix015)
Supplement: Response_to_reviewer_comments_Original_Submission.pdf [file gix015_Response_to_reviewer_comments_Original_Submission.pdf]

We thank the reviewers for their thorough evaluation of our manuscript and for the thoughtful and very helpful comments. We address each of these below and in the attached revision.

Reviewer reports:

Reviewer #1: The authors present a collection of Galaxy enabled tools for the detection of somatic mutations in cancer samples at the whole genome or exome level. The authors also report the improvement via parallelization (parallel execution of computationally expensive tasks on distributed computing) of exiting bioinformatics tools within Galaxy. The authors demonstrated the usability of their toolkit on a cohort of 96 large B-cell lymphomas. The work presented is in my opinion original, novel and relevant for the cancer research community. The following items should be addressed prior publication:

The authors compare the use of Galaxy with other open source and commercial workflow and pipeline development tools, highlighting the advantages of the use of Galaxy. Authors should also discuss potential disadvantages (data transfer consideration in and out of Galaxy, latency, less flexibility than the command line, scalability, etc.)

Response:
We feel that much of this has already been discussed in other relevant literature. Nonetheless, we have added the following text to clarify that we do not feel Galaxy is appropriate in all situations:

“To facilitate scaling of our applications to whole genome and exome data to the extent currently possible in this framework, and to accelerate the analysis of exomes, we established new methods to accomplish parallelization in Galaxy. It is important to note that Galaxy is not particularly well suited to certain large-scale analyses due to how data transfer tasks are handled, the internalization of some processes (e.g. bam indexing) and centralization of intermediate files generated by tools. We hope that ongoing development of the Galaxy codebase will improve on these and suggest that command-line equivalents to our workflows such as those offered by the Kronos software are worthy of consideration in larger-scale projects. Ongoing development of the Galaxy API may also address some of these issues.”

For testing multithreaded applications, users mention that they used a linux server; no hardware configuration is provided for this test environment, authors need to describe the required minimal configuration for such environment. Performance of multithreaded applications was not tested by this reviewer.

Response:
We have provided the following details in the manuscript in response to this comment. “Local testing of tools was performed on a Dell PowerEdge R430 Server with 2x Intel Xeon Processors (32 threads total) and 384 Gb of RAM” on line 131 of the revised manuscript.

The authors mentioned they used one r3.8xlarge AWS node as master and five r3.8xlarge nodes as workers using CloudMan, how CloudMan compares with other virtual HPC cluster management tools on the cloud? Why did they decided to use CloudMan instead of say AWS native CfnCluster or elasticHPC package?

Response:
CloudMan was used because of the convenience features it offers for handling Galaxy-based clusters. With better integration of Docker throughout the text and testing on Google Cloud in this revision, we now acknowledge that alternative configurations and methods for deploying clusters exist and that CloudMan is only one such option. For example, we have added the following sentence:
“We have successfully run our tools and workflows using AWS cloud computing (with CloudMan), which provides a cluster environment to any research lab and on Google cloud, which facilitates cluster management of Docker-based instances using Kubernetes.”.

Ultimately, testing our system with many available configurations and cloud providers could provide sufficient work to form a separate study and we feel this exploration would be valuable but beyond the scope of this work.

Authors should clarify that the split-the-input-data technique they used could only apply when there is no task-interdependency (i.e. nodes do not need to communicate with each other in order to produce results).

Response:
This has been clarified in the text.

Authors briefly mention that they "Implemented workflows that performed the required annotation and pre-processing of raw mutation outputs", however authors should briefly describe how:

* After SNVs and CNVs were detected with different tools (like Strelka and Sequenza) on individual tumor-normal pairs, how a consensus calls was selected at the sample level, and how multiple-sample calls were integrated?
* How did authors handle disagreement/inconsistences in the SNV/CNV calls?
* What were the filtering steps performed to go from raw mutation to selected/final list of candidates to be annotated?

Response:
We agree that these are important considerations. The thresholds used by each tool are set to defaults that, where possible, adhere to guidelines set by the authors or were demonstrated in our hands or elsewhere to offer a good balance of sensitivity and specificity (e.g. in the ICGC Dream SNV calling challenge). This is detailed in the manuscript around line 145:

“There are numerous algorithms available to perform standard analytical tasks such as variant calling and CNV detection, each offering different balances of usability, computational efficiency and accuracy. As such, selection of ideal tools and parameters is non-trivial. We implemented tools representing some of the more commonly cited options and include many that performed favorably in ICGC-TCGA DREAM challenges. As each tool can be configured with a number of parameters, which can be tuned for improved accuracy, we leverage results from the DREAM challenge to assist in selecting the more accurate algorithms and in setting sensible default parameters. ”

We follow with a description of our ensemble approach to find variants that are consistent among different tools:

“As ensemble approaches tend to provide increased accuracy, we developed a tool to integrate variant calls from multiple algorithms using a simple voting scheme (Additional Items: Figure S1). ”
We note that the legend for that supplemental figure was rather brief, so we have provided further details in this version. The legend now reads as follows:

“Figure S1. An ensemble approach to detect somatic SNVs.
The ensembl_vcf tool was developed to implement a simple voting-based approach to select high-confidence SNV calls. The tool is given the output of multiple variant callers and selects variants detected by a user-specified minimum proportion of tools. This example workflow runs four variant callers (strelka, mutationSeq, RADIA and SomaticSniper) and runs vcf2maf to annotate the resulting list of variants with support from the majority of the tools.”

Authors do not report any runtime metrics; I think this is very important since the whole point of "improvement via parallelization" is to reduce runtime. Authors should at least give the reader an idea of the performance increase. For instances, for whole genome sequence data from human samples, strelka requires approximately 1 core-hour per 2x of combined sample reference coverage e.g. analyzing a human tumor-normal sample pair sequenced to 60x and 40x respectively should take approximately 50 core-hours,

how many folds this time was reduced by parallelization?

Response:
This is an important point. We collected a large amount of runtime metrics during the testing of some of our core sets of tools and workflows including Strelka, Sequenza and Titan. We have included a summary of these results (new Fig. 2 and Table 2). These are also discussed in the new version of the manuscript, with relevant text that has been included as a new section (~lines 160-175).

Installation:

Local installation following provided instructions was strait forward, when installing on a Mac or Linux environment, build-in genomes are not provided, forcing users to select reference genomes from the user's history, this requires the "Get Data/Upload File' feature on Galaxy, however, the default configuration o available space for user's datasets is really small, error 28 (No space left on device) was reported by Galaxy after uploading files 'test.tumor.bam' + 'test.normal.bam' + hg19 reference genome (or provided test file) (all together about 45 MB in size). Authors should provide clear instructions on how to extend the storage space to be used by this Galaxy installation. See error logs bellow:

==> /home/galaxy/logs/slurmctld.log <==
[2016-12-08T20:05:35.160] error: Error writing file /tmp/slurm/assoc_mgr_state.new, No space left on device
[2016-12-08T20:05:35.160] error: Error writing file /tmp/slurm/assoc_usage.new, No space left on device
[2016-12-08T20:05:35.160] error: Error writing file /tmp/slurm/qos_usage.new, No space left on device
[2016-12-08T20:05:35.161] error: The modification time of /tmp/slurm/job_state moved backwards by 6701 seconds
[2016-12-08T20:05:35.161] error: The clock of the file system and this computer appear to not be synchronized
[2016-12-08T20:05:35.161] error: Error writing file /tmp/slurm/job_state.new, No space left on device
[2016-12-08T20:05:35.162] error: Error writing file /tmp/slurm/node_state.new, No space left on device
[2016-12-08T20:05:35.162] error: Error writing file /tmp/slurm/part_state.new, No space left on device
[2016-12-08T20:05:35.162] error: Error writing file /tmp/slurm/resv_state.new, No space left on device
[2016-12-08T20:05:35.163] error: Error writing file /tmp/slurm/trigger_state.new, No space left on device
[2016-12-08T20:10:35.680] error: Error writing file /tmp/slurm/assoc_mgr_state.new, No space left on device
[2016-12-08T20:10:35.681] error: Error writing file /tmp/slurm/assoc_usage.new, No space left on device
[2016-12-08T20:10:35.681] error: Error writing file /tmp/slurm/qos_usage.new, No space left on device
[2016-12-08T20:10:35.682] error: The modification time of /tmp/slurm/job_state moved backwards by 7001 seconds
[2016-12-08T20:10:35.682] error: The clock of the file system and this computer appear to not be synchronized
[2016-12-08T20:10:35.682] error: Error writing file /tmp/slurm/job_state.new, No space left on device
[2016-12-08T20:10:35.683] error: Error writing file /tmp/slurm/node_state.new, No space left on device
[2016-12-08T20:10:35.683] error: Error writing file /tmp/slurm/part_state.new, No space left on device
[2016-12-08T20:10:35.683] error: Error writing file /tmp/slurm/resv_state.new, No space left on device
[2016-12-08T20:10:35.683] error: Error writing file /tmp/slurm/trigger_state.new, No space left on device

SomaticSniper SNV test
Reported: Tool error, Unable to run this job due to a cluster error, please retry it later

RADIA SNV test
Reported: Tool error, Unable to run this job due to a cluster error, please retry it later

AWS installation using Docker:

Installation was easy (although time consuming) on AWS. Standard Galaxy tools inherited from main distribution were not tested.

RADIA tool reported error:

python: can't open file '/radia_src/scripts/radia.py': [Errno 2] No such file or directory

MutationSeq tool reported error:
/tool_deps/mutationseq_python_environment/4.3.6/morinlab/package_mutationseq_python_environment_4_3_6/2ed80b397b53/bin/python2.7: can't open file '/classify.py': [Errno 2] No such file or directory

Pindel tool reported error:
Fatal error: Exit code 2 (Failure)
Can't open perl script "/vcf2maf.pl": No such file or directory

Response:
Without details on which installation method (Docker?) was used here, it is impossible to be certain what is causing this issue. This error seems to relate to the disk storage allocated to the VM or Docker container being used. If using Docker, the container should be launched as described in Bjorn Gruening's documentation such that the folders written to by Galaxy are all exported to a separate local directory that is under the direct control of the user. If using Galaxy on Amazon, the problem is less clear but may relate to the local disk space selected by the reviewer when choosing an instance.

With regards to the specific errors not relating to disk space, we have made numerous fixes to installation errors and environment issues since the original submission. Although we cannot be certain that every issue encountered by this user is fixed, we have squashed many bugs and intend to continue this process as more users test the software. We are also working directly with members of the Galaxy community including Bjorn Gruening to push all of these tools into the main toolshed. We fully expect more bugs to be found and fixed in that process. We also refer this reviewer to our response to reviewer two regarding the ongoing effort to provide a more complete Docker image on Google Cloud that would contain the common reference files needed for running the tools.

The figures included in the integrated manuscript are not publication ready; authors should produce high quality, high-resolution images in accordance with the journal requirements.

Response:
We have prepared high resolution image files for each of the original and new figures in this revision.

Reviewer #2: Data generated from massively parallel sequencing techniques is very large (in the range of several hundred GB), and requires large resources and expertise in processing. This remains a challenge for labs and academic/research groups that do not dedicated resources or bioinformaticians. The authors have produced a collection of Galaxy tools for detecting somatic genetic alterations from cancer genome and exome data. They developed new methods for parallelization to accelerate runtime and demonstrated their usability on cloud-based infrastructure and commodity hardware.

My comments are below:

- Big data processing and storage is a real challenge in bioinformatics. Not many scientists have realized this, so I'm happy to see the authors trying to solve issues in this realm.

In view of the challenges in Big data processing and storage, new initiatives like the Cancer Cloud Pilot have

recently been introduced. Such programs offer cloud based platform with numerous tools for analysis and processing of large scale raw data. For popular datasets like TCGA, they provide pre-processed data, so that user does not have to spend time and money processing the raw data. Currently, there are 3 Cancer Cloud Pilot projects: one offered by Seven Bridges which is a Galaxy like GUI system, and other solutions offered by Broad and ISB (which are more command line based). How does the solution put forward by the authors compare to (or better than) initiatives like cancer cloud pilot ?

Response:
Our group was part of the initiative that conceptualized and established the Cancer Genome Collaboratory (https://www.cancercollaboratory.org/), which is based on OpenStack. We are working towards deploying a Galaxy system in this environment that will host our toolkit to all users of the Collaboratory. We are certainly interested in extending this concept to the Cancer Cloud projects and note that the Google Cloud is used by those cited above. As we have now succeeded in deploying our Galaxy toolkit in a Docker container on Google Cloud, we are certainly hoping that users of these systems (if not our group) will make use of Galaxy on these systems.

- The solutions proposed by the authors seems to involve use of Docker and launching Galaxy on AWS Elastic Cloud Compute (EC2) using CloudMan. So that means, implementation of these tools requires technical expertise and knowledge of Docker. I think the authors need to explain what the level of knowledge and expertise is expected from a user.
- How much expertise in Docker/Amazon cloud Ubuntu instance is required from a user to implement this solution ?

Response:

Making this more straightforward is ongoing process. As pointed out by Reviewer 1, users also must install (or upload) their own reference files if using the Dockerfile to build a fresh instance. We hope this will improve soon but require support from cloud providers who must host our larger images that contain reference data files. We have added the following text: “This Docker image was successfully built with automatic installation of tools and dependencies on our local Linux server and on the Google Cloud. We are working with this service to release an instance with reference genomes pre-installed that can be directly launched with minimal knowledge of Docker.”

Can this solution be used on public Galaxy servers (which are free to use) ?

Response:
Since the submission of this manuscript, we have become engaged with the Galaxy community to bring all of the tools presented here into the Main Toolshed. This is the first step in these becoming available in the public servers. We strive to make this happen but cannot make any guarantees.

The authors mention they have used "custom Ubuntu installation", "custom workflows and scripts". Is there detailed documentation online on how to set up this customized Galaxy infrastructure ?

Response:
The mention of custom installation was in reference to the possibility of users installing the tools on their own instance without using Docker or the cloud services. The installation of Galaxy on different Linux flavours is fairly well documented already. Any dependencies beyond those expected on a basic Galaxy system are detailed in the Dockerfile but are specific to one custom Ubuntu Linux instance we created. Hence, the apt-get commands in that file could be used to reproduce that environment without using Docker.

For laboratories lacking dedicated technology resources, the solution put forward by the authors is to perform the analysis on the cloud. Any analysis or computing done on the cloud costs money. One challenge of working with the cloud is the difficulty in estimating set-up and run-time costs. If not estimated and set

up correctly, the costs often become overwhelmingly large. The authors need to discuss/estimate how much one analysis will cost on the cloud infrastructure they set up.

Response:
This is an important point. We collected a large amount of runtime metrics during the testing of some of our core sets of tools and workflows including Strelka, Sequenza and Titan. We have included a summary of these results (new Fig. 2 and Table 2). These are also discussed in the new version of the manuscript, with relevant text that has been included as a new section (~lines 160-175).

- Its not clear intervals were used for variant calling (manuscript says "automatic interval selection"). Typically Variant calling algorithms are run per chromosome. Variant calling algorithm using interval smaller than a chromosome typically will not perform well because it will not have enough information about other regions of the genome for predicting insertions, deletions, etc.

Response:
We agree with this issue and have specifically avoided using intervals smaller than the chromosome length. This is clarified now on line 124.

Other minor comments:
- Planemo - It probably deserves to be defined in the paper ?

Response:
This is now defined briefly when referenced on line 95

- "All repositories are stored on the public Galaxy test toolshed, which allows users to automatically install any tool" -- add link to the tool shed here.

Response:
Link has been added where suggested.

- The authors should clearly list which tools they have created new (and what the tool does), and which tools they customized or parallelized. It was not clear from the paper. Table 1 only lists "Main tools currently comprising the cancer genomics toolkit."

Response:
This has been clarified in Table one by adding the following legend:

∞New tool or visualization method created for this project. §New implementation of tool for existing software. ‡Existing Galaxy tool modified or extended for this project.
