# Supplementary material for: Enhancing knowledge discovery from cancer genomics data with Galaxy
Source: Gigascience. 2017 Mar 9;6(5):1–13. doi: 10.1093/gigascience/gix015 (PMC5437943; doi:10.1093/gigascience/gix015)
Supplement: Response_to_reviewer_comments_Revision_1.pdf [file gix015_Response_to_reviewer_comments_Revision_1.pdf]

Minor comments:
- I visited https://testtoolshed.g2.bx.psu.edu/, and wanted to try out a test run of your package to see if I could get those pretty pictures as shown in Figure 3. But it looks like its not possible to run any tools in the test tool shed.
Would be nice to be able to test and run this workflow on a small test sample on Galaxy.

Response: We cannot compel the Galaxy community to host our tools in their main (public) instance so the tools, unfortunately, can only be run on instances for which the user has admin privileges. We hope that our Docker release will allow most users to overcome this hurdle. Test data is now provided for all display items in the paper that were generated by our tools as detailed below and now referred to in the manuscript.

- These days there is a lot of focus on reproducibility of results. I know the authors have a section "Towards reproducible and distributable workflows for cancer genome analysis" which talks about reproducible and distributable workflows. The authors have also demonstrated the utility of their Galaxy Cancer Genomics Toolkit by applying the included workflows to a large cohort of DLBCL patients. It would be nice if the output of these workflows applied to this dataset (a few samples from this dataset or any test dataset) could be make public either through Galaxy, or just shared through an FTP server , google drive, or something similar. This will help any user who wants to reproduce this workflow, can use those output files to determine whether they are on the right track or not. Even of the input BAM or BED files may be large, the output files are typically small.

Response - we have now included inputs, reference files and results for each of the following analyses in the github repository along with documentation: GISTIC, oncocircos, oncodriveclust, oncodriveFM (with oncoprintplus and lollipop plots) and the other maftools-based plots.

- Include Link to the Galaxy tool shed in the "Availability" section

Response: Done
