# Supplementary material for: Enhancing knowledge discovery from cancer genomics data with Galaxy
Source: Gigascience. 2017 Mar 9;6(5):1–13. doi: 10.1093/gigascience/gix015 (PMC5437943; doi:10.1093/gigascience/gix015)
Supplement: Reviewer_1_Report_(Original_Submission).pdf [file gix015_Reviewer_1_Report_(Original_Submission).pdf]

Reviewer Report

Title: "Enhancing Knowledge Discovery from Cancer Genomics Data with Galaxy"

Version: Original Submission **Date:** 12/12/2016

Reviewer name: Jorge Andrade, Ph.D.

Reviewer Comments to Author:

The authors present a collection of Galaxy enabled tools for the detection of somatic mutations in cancer samples at the whole genome or exome level. The authors also report the improvement via parallelization (parallel execution of computationally expensive tasks on distributed computing) of exiting bioinformatics tools within Galaxy. The authors demonstrated the usability of their toolkit on a cohort of 96 large B-cell lymphomas. The work presented is in my opinion original, novel and relevant for the cancer research community. The following items should be addressed prior publication:

The authors compare the use of Galaxy with other open source and commercial workflow and pipeline development tools, highlighting the advantages of the use of Galaxy. Authors should also discuss potential disadvantages (data transfer consideration in and out of Galaxy, latency, less flexibility than the command line, scalability, etc.)

For testing multithreaded applications, users mention that they used a linux server; no hardware configuration is provided for this test environment, authors need to describe the required minimal configuration for such environment. Performance of multithreaded applications was not tested by this reviewer.

The authors mentioned they used one r3.8xlarge AWS node as master and five r3.8xlarge nodes as workers using CloudMan, how CloudMan compares with other virtual HPC cluster management tools on the cloud? Why did they decided to use CloudMan instead of say AWS native CfnCluster or elasticHPC package?

Authors should clarify that the split-the-input-data technique they used could only apply when there is no task-interdependency (i.e. nodes do not need to communicate with each other in order to produce results).

Authors briefly mention that they "Implemented workflows that performed the required annotation and pre-processing of raw mutation outputs", however authors should briefly describe how:

* After SNVs and CNVs were detected with different tools (like Strelka and Sequenza) on individual tumor-normal pairs, how a consensus calls was selected at the sample level, and how multiple-sample calls were integrated?
* How did authors handle disagreement/inconsistences in the SNV/CNV calls?
* What were the filtering steps performed to go from raw mutation to selected/final list of candidates to be annotated?

Authors do not report any runtime metrics; I think this is very important since the whole point of "improvement via parallelization" is to reduce runtime. Authors should at least give the reader an idea of the performance increase. For instances, for whole genome sequence data from human samples, strelka requires approximately 1 core-hour per 2x of combined sample reference coverage e.g. analyzing a human tumor-normal sample pair sequenced to 60x and 40x respectively should take approximately 50 core-hours, how many folds this time was reduced by parallelization?

Installation:

Local installation following provided instructions was strait forward, when installing on a Mac or Linux environment, build-in genomes are not provided, forcing users to select reference genomes from the user's history, this requires the "Get Data/Upload File' feature on Galaxy, however, the default configuration o available space for user's datasets is really small, error 28 (No space left on device) was reported by Galaxy after uploading files 'test.tumor.bam' + 'test.normal.bam' + hg19 reference genome (or provided test file) (all together about 45 MB in size). Authors should provide clear instructions on how to extend the storage space to be used by this Galaxy installation. See error logs bellow:

==> /home/galaxy/logs/slurmctld.log <==

[2016-12-08T20:05:35.160] error: Error writing file /tmp/slurm/assoc_mgr_state.new, No space left on device

[2016-12-08T20:05:35.160] error: Error writing file /tmp/slurm/assoc_usage.new, No space left on device

[2016-12-08T20:05:35.160] error: Error writing file /tmp/slurm/qos_usage.new, No space left on device

[2016-12-08T20:05:35.161] error: The modification time of /tmp/slurm/job_state moved backwards by 6701 seconds

[2016-12-08T20:05:35.161] error: The clock of the file system and this computer appear to not be synchronized

[2016-12-08T20:05:35.161] error: Error writing file /tmp/slurm/job_state.new, No space left on device

[2016-12-08T20:05:35.162] error: Error writing file /tmp/slurm/node_state.new, No space left on device

[2016-12-08T20:05:35.162] error: Error writing file /tmp/slurm/part_state.new, No space left on device

[2016-12-08T20:05:35.162] error: Error writing file /tmp/slurm/resv_state.new, No space left on device

[2016-12-08T20:05:35.163] error: Error writing file /tmp/slurm/trigger_state.new, No space left on device

[2016-12-08T20:10:35.680] error: Error writing file /tmp/slurm/assoc_mgr_state.new, No space left on device

[2016-12-08T20:10:35.681] error: Error writing file /tmp/slurm/assoc_usage.new, No space left on device

[2016-12-08T20:10:35.681] error: Error writing file /tmp/slurm/qos_usage.new, No space left on device

[2016-12-08T20:10:35.682] error: The modification time of /tmp/slurm/job_state moved backwards by 7001 seconds

[2016-12-08T20:10:35.682] error: The clock of the file system and this computer appear to not be synchronized

[2016-12-08T20:10:35.682] error: Error writing file /tmp/slurm/job_state.new, No space left on device

[2016-12-08T20:10:35.683] error: Error writing file /tmp/slurm/node_state.new, No space left on device

[2016-12-08T20:10:35.683] error: Error writing file /tmp/slurm/part_state.new, No space left on device

[2016-12-08T20:10:35.683] error: Error writing file /tmp/slurm/resv_state.new, No space left on device

[2016-12-08T20:10:35.683] error: Error writing file /tmp/slurm/trigger_state.new, No space left on device

SomaticSniper SNV test

Reported: Tool error, Unable to run this job due to a cluster error, please retry it later

RADIA SNV test

Reported: Tool error, Unable to run this job due to a cluster error, please retry it later

AWS installation using Docker:

Installation was easy (although time consuming) on AWS. Standard Galaxy tools inherited from main distribution were not tested.

RADIA tool reported error:

python: can't open file '/radia_src/scripts/radia.py': [Errno 2] No such file or directory

MutationSeq tool reported error:

/tool_deps/mutationseq_python_environment/4.3.6/morinlab/package_mutationseq_python_environment_4_3_6/2ed80b397b53/bin/python2.7: can't open file '/classify.py': [Errno 2] No such file or directory

Pindel tool reported error:

Fatal error: Exit code 2 (Failure)

Can't open perl script "/vcf2maf.pl": No such file or directory

The figures included in the integrated manuscript are not publication ready; authors should produce high quality, high-resolution images in accordance with the journal requirements

Level of Interest

Please indicate how interesting you found the manuscript: An article of importance in its field

Quality of Written English

Please indicate the quality of language in the manuscript: Acceptable

Declaration of Competing Interests

Please complete a declaration of competing interests, considering the following questions:

- Have you in the past five years received reimbursements, fees, funding, or salary from an organisation that may in any way gain or lose financially from the publication of this manuscript, either now or in the future?
- Do you hold any stocks or shares in an organisation that may in any way gain or lose financially from the publication of this manuscript, either now or in the future?
- Do you hold or are you currently applying for any patents relating to the content of the manuscript?
- Have you received reimbursements, fees, funding, or salary from an organization that holds or has applied for patents relating to the content of the manuscript?
- Do you have any other financial competing interests?
- Do you have any non-financial competing interests in relation to this paper?

If you can answer no to all of the above, write 'I declare that I have no competing interests' below. If your reply is yes to any, please give details below.

'I declare that I have no competing interests'

I agree to the open peer review policy of the journal. I understand that my name will be included on my report to the authors and, if the manuscript is accepted for publication, my named report including any attachments I upload will be posted on the website along with the authors' responses. I agree for my report to be made available under an Open Access Creative Commons CC-BY license (http://creativecommons.org/licenses/by/4.0/). I understand that any comments which I do not wish to be included in my named report can be included as confidential comments to the editors, which will not be published.

I agree to the open peer review policy of the journal
