# Supplementary material for: Enhancing knowledge discovery from cancer genomics data with Galaxy
Source: Gigascience. 2017 Mar 9;6(5):1–13. doi: 10.1093/gigascience/gix015 (PMC5437943; doi:10.1093/gigascience/gix015)
Supplement: Reviewer_2_Report_(Original_Submission).pdf [file gix015_Reviewer_2_Report_(Original_Submission).pdf]

Reviewer Report

Title: "Enhancing Knowledge Discovery from Cancer Genomics Data with Galaxy"

Version: Original Submission **Date:** 12/22/2016

Reviewer name: Krithika Bhuvaneshwar

Reviewer Comments to Author:

Data generated from massively parallel sequencing techniques is very large (in the range of several hundred GB), and requires large resources and expertise in processing. This remains a challenge for labs and academic/research groups that do not dedicated resources or bioinformaticians. The authors have produced a collection of Galaxy tools for detecting somatic genetic alterations from cancer genome and exome data. They developed new methods for parallelization to accelerate runtime and demonstrated their usability on cloud-based infrastructure and commodity hardware.

My comments are below:

- Big data processing and storage is a real challenge in bioinformatics. Not many scientists have realized this, so I'm happy to see the authors trying to solve issues in this realm.

- In view of the challenges in Big data processing and storage, new initiatives like the Cancer Cloud Pilot have recently been introduced. Such programs offer cloud based platform with numerous tools for analysis and processing of large scale raw data. For popular datasets like TCGA, they provide pre-processed data, so that user does not have to spend time and money processing the raw data. Currently, there are 3 Cancer Cloud Pilot projects: one offered by Seven Bridges which is a Galaxy like GUI system, and other solutions offered by Broad and ISB (which are more command line based). How does the solution put forward by the authors compare to (or better than) initiatives like cancer cloud pilot ?

- The solutions proposed by the authors seems to involve use of Docker and launching Galaxy on AWS Elastic Cloud Compute (EC2) using CloudMan. So that means, implementation of these tools requires technical expertise and knowledge of Docker. I think the authors need to explain what the level of knowledge and expertise is expected from a user.

- How much expertise in Docker/Amazon cloud Ubuntu instance is required from a user to implement this solution ?

- Can this solution be used on public Galaxy servers (which are free to use) ?

- The authors mention they have used "custom Ubuntu installation", "custom workflows and scripts". Is there detailed documentation online on how to set up this customized Galaxy infrastructure ?

- For laboratories lacking dedicated technology resources, the solution put forward by the authors is to perform the analysis on the cloud. Any analysis or computing done on the cloud costs money. One challenge of working with the cloud is the difficulty in estimating set-up and run-time costs. If not estimated and set up correctly, the costs often become overwhelmingly large. The authors need to discuss/estimate how much one analysis will cost on the cloud infrastructure they set up.

- Its not clear intervals were used for variant calling (manuscript says "automatic interval selection"). Typically Variant calling algorithms are run per chromosome. Variant calling algorithm using interval smaller than a chromosome typically will not perform well because it will not have enough information about other regions of the genome for predicting insertions, deletions, etc.

Other minor comments:

- Planemo - It probably deserves to be defined in the paper ?

- "All repositories are stored on the public Galaxy test toolshed, which allows users to automatically install any tool" -- add link to the tool shed here.

- The authors should clearly list which tools they have created new (and what the tool does), and which tools they customized or parallelized. It was not clear from the paper. Table 1 only lists "Main tools currently comprising the cancer genomics toolkit."

Level of Interest

Please indicate how interesting you found the manuscript: An article of importance in its field

Quality of Written English

Please indicate the quality of language in the manuscript: Needs some language corrections before being published

Declaration of Competing Interests

Please complete a declaration of competing interests, considering the following questions:

- Have you in the past five years received reimbursements, fees, funding, or salary from an organisation that may in any way gain or lose financially from the publication of this manuscript, either now or in the future?
- Do you hold any stocks or shares in an organisation that may in any way gain or lose financially from the publication of this manuscript, either now or in the future?
- Do you hold or are you currently applying for any patents relating to the content of the manuscript?
- Have you received reimbursements, fees, funding, or salary from an organization that holds or has applied for patents relating to the content of the manuscript?
- Do you have any other financial competing interests?
- Do you have any non-financial competing interests in relation to this paper?

If you can answer no to all of the above, write 'I declare that I have no competing interests' below. If your reply is yes to any, please give details below.

I declare that I have no competing interests

I agree to the open peer review policy of the journal. I understand that my name will be included on my report to the authors and, if the manuscript is accepted for publication, my named report including any attachments I upload will be posted on the website along with the authors' responses. I agree for my report to be made available under an Open Access Creative Commons CC-BY license (http://creativecommons.org/licenses/by/4.0/). I understand that any comments which I do not wish to be included in my named report can be included as confidential comments to the editors, which will not be published.

I agree to the open peer review policy of the journal
