# Supplementary material for: Enhancing knowledge discovery from cancer genomics data with Galaxy
Source: Gigascience. 2017 Mar 9;6(5):1–13. doi: 10.1093/gigascience/gix015 (PMC5437943; doi:10.1093/gigascience/gix015)
Supplement: Reviewer_2_Report_(Revision_1).pdf [file gix015_Reviewer_2_Report_(Revision_1).pdf]

Reviewer Report

Title: "Enhancing Knowledge Discovery from Cancer Genomics Data with Galaxy"

Version: Revision 1 **Date:** 3/1/2017

Reviewer name: Krithika Bhuvaneshwar

Reviewer Comments to Author:

The authors have done a thorough job in responding to reviewer comments, and I approve this manuscript for publication in this journal.

Minor comments:

- I visited https://testtoolshed.g2.bx.psu.edu/, and wanted to try out a test run of your package to see if I could get those pretty pictures as shown in Figure 3. But it looks like its not possible to run any tools in the test tool shed.

Would be nice to be able to test and run this workflow on a small test sample on Galaxy.

- These days there is a lot of focus on reproducibility of results. I know the authors have a section "Towards reproducible and distributable workflows for cancer genome analysis" which talks about reproducible and distributable workflows. The authors have also demonstrated the utility of their Galaxy Cancer Genomics Toolkit by applying the included workflows to a large cohort of DLBCL patients. It would be nice if the output of these workflows applied to this dataset (a few samples from this dataset or any test dataset) could be make public either through Galaxy, or just shared through an FTP server , google drive, or something similar. This will help any user who wants to reproduce this workflow, can use those output files to determine whether they are on the right track or not. Even of the input BAM or BED files may be large, the output files are typically small.

- Include Link to the Galaxy tool shed in the "Availability" section

Level of Interest

Please indicate how interesting you found the manuscript: An article whose findings are important to those with closely related research interests

Quality of Written English

Please indicate the quality of language in the manuscript: Acceptable

Declaration of Competing Interests

Please complete a declaration of competing interests, considering the following questions:

- Have you in the past five years received reimbursements, fees, funding, or salary from an organisation that may in any way gain or lose financially from the publication of this manuscript, either now or in the future?
- Do you hold any stocks or shares in an organisation that may in any way gain or lose financially from the publication of this manuscript, either now or in the future?
- Do you hold or are you currently applying for any patents relating to the content of the manuscript?
- Have you received reimbursements, fees, funding, or salary from an organization that holds or has applied for patents relating to the content of the manuscript?
- Do you have any other financial competing interests?
- Do you have any non-financial competing interests in relation to this paper?

If you can answer no to all of the above, write 'I declare that I have no competing interests' below. If your reply is yes to any, please give details below.

I declare that I have no competing interests

I agree to the open peer review policy of the journal. I understand that my name will be included on my report to the authors and, if the manuscript is accepted for publication, my named report including any attachments I upload will be posted on the website along with the authors' responses. I agree for my report to be made available under an Open Access Creative Commons CC-BY license (http://creativecommons.org/licenses/by/4.0/). I understand that any comments which I do not wish to be included in my named report can be included as confidential comments to the editors, which will not be published.

I agree to the open peer review policy of the journal
